# Discovery and Fine-Mapping of Glycaemic and Obesity-Related Trait Loci Using High-Density Imputation

**DOI:** 10.1371/journal.pgen.1005230

**Published:** 2015-07-01

**Authors:** Momoko Horikoshi, Reedik Mӓgi, Martijn van de Bunt, Ida Surakka, Antti-Pekka Sarin, Anubha Mahajan, Letizia Marullo, Gudmar Thorleifsson, Sara Hӓgg, Jouke-Jan Hottenga, Claes Ladenvall, Janina S. Ried, Thomas W. Winkler, Sara M. Willems, Natalia Pervjakova, Tõnu Esko, Marian Beekman, Christopher P. Nelson, Christina Willenborg, Steven Wiltshire, Teresa Ferreira, Juan Fernandez, Kyle J. Gaulton, Valgerdur Steinthorsdottir, Anders Hamsten, Patrik K. E. Magnusson, Gonneke Willemsen, Yuri Milaneschi, Neil R. Robertson, Christopher J. Groves, Amanda J. Bennett, Terho Lehtimӓki, Jorma S. Viikari, Johan Rung, Valeriya Lyssenko, Markus Perola, Iris M. Heid, Christian Herder, Harald Grallert, Martina Müller-Nurasyid, Michael Roden, Elina Hypponen, Aaron Isaacs, Elisabeth M. van Leeuwen, Lennart C. Karssen, Evelin Mihailov, Jeanine J. Houwing-Duistermaat, Anton J. M. de Craen, Joris Deelen, Aki S. Havulinna, Matthew Blades, Christian Hengstenberg, Jeanette Erdmann, Heribert Schunkert, Jaakko Kaprio, Martin D. Tobin, Nilesh J. Samani, Lars Lind, Veikko Salomaa, Cecilia M. Lindgren, P. Eline Slagboom, Andres Metspalu, Cornelia M. van Duijn, Johan G. Eriksson, Annette Peters, Christian Gieger, Antti Jula, Leif Groop, Olli T. Raitakari, Chris Power, Brenda W. J. H. Penninx, Eco de Geus, Johannes H. Smit, Dorret I. Boomsma, Nancy L. Pedersen, Erik Ingelsson, Unnur Thorsteinsdottir, Kari Stefansson, Samuli Ripatti, Inga Prokopenko, Mark I. McCarthy, Andrew P. Morris, ENGAGE Consortium

**Affiliations:** 1 Wellcome Trust Centre for Human Genetics, University of Oxford, Oxford, United Kingdom; 2 Oxford Centre for Diabetes, Endocrinology and Metabolism, University of Oxford, Oxford, United Kingdom; 3 Estonian Genome Center, University of Tartu, Tartu, Estonia; 4 Institute for Molecular Medicine Finland FIMM, University of Helsinki, Helsinki, Finland; 5 National Institute for Health and Welfare, Helsinki, Finland; 6 Department of Life Sciences and Biotechnology, University of Ferrara, Ferrara, Italy; 7 deCode Genetic - Amgen Inc, Reykjavik, Iceland; 8 Department of Medical Epidemiology and Biostatistics, Karolinska Institutet, Stockholm, Sweden; 9 Department of Medical Sciences, Molecular Epidemiology, and Science for Life Laboratory, Uppsala University, Uppsala, Sweden; 10 Department of Biological Psychology, VU University Amsterdam, Amsterdam, The Netherlands; 11 Department of Clinical Sciences, Diabetes and Endocrinology, Lund University Diabetes Centre, Skåne University Hospital, Malmö, Sweden; 12 Institute of Genetic Epidemiology, Helmholtz Zentrum München - German Research Center for Environmental Health, Neuherberg, Germany; 13 Department of Genetic Epidemiology, Institute of Epidemiology and Preventive Medicine, University of Regensburg, Regensburg, Germany; 14 Genetic Epidemiology Unit, Department of Epidemiology, Erasmus University Medical Center, Rotterdam, The Netherlands; 15 Division of Endocrinology and Center for Basic and Translational Obesity Research, Children’s Hospital, Boston, Massachusetts, United States of America; 16 Program in Medical and Population Genetics, Broad Institute, Cambridge, Massachusetts, United States of America; 17 Department of Genetics, Harvard Medical School, Boston, Massachusetts, United States of America; 18 Department of Molecular Epidemiology, Leiden University Medical Center, Leiden, The Netherlands; 19 Netherlands Consortium for Healthy Ageing, Leiden, The Netherlands; 20 Department of Cardiovascular Sciences, University of Leicester, Leicester, United Kingdom; 21 National Institute for Health Research Leicester Cardiovascular Disease Biomedical Research Unit, Glenfield Hospital, Leicester, United Kingdom; 22 Institute for Integrative and Experimental Genomics, University of Lübeck, Lübeck, Germany; 23 DZHK German Center for Cardiovascular Research, Partner Site Hamburg/Kiel/Lübeck, Lübeck, Germany; 24 Cardiovascular Genetics and Genomics Group, Atherosclerosis Research Unit, Department of Medicine Solna, Karolinska Institutet, Stockholm, Sweden; 25 Department of Psychiatry, VU University Medical Center, Amsterdam, The Netherlands; 26 Department of Clinical Chemistry, Fimlab Laboratories and School of Medicine, University of Tampere, Tampere, Finland; 27 Department of Medicine, University of Turku and Division of Medicine, Turku University Hospital, Turku, Finland; 28 European Molecular Biology Laboratory, European Bioinformatics Institute (EMBL-EBI), Wellcome Trust Genome Campus, Hinxton, United Kingdom; 29 Steno Diabetes Center A/S, Gentofte, Denmark; 30 Institute for Clinical Diabetology, German Diabetes Center, Leibniz Institute for Diabetes Research at Heinrich Heine University Düsseldorf, Düsseldorf, Germany; 31 German Center for Diabetes Research (DZD e.V.), Partner Düsseldorf, Germany; 32 Research Unit of Molecular Epidemiology, Helmholtz Zentrum München - German Research Center for Environmental Health, Neuherberg, Germany; 33 Institute of Epidemiology II, Helmholtz Zentrum München - German Research Center for Environmental Health, Neuherberg, Germany; 34 Department of Medicine I, University Hospital Grosshadern, Ludwig-Maximilians-Universität, Munich, Germany; 35 Institute of Medical Informatics, Biometry and Epidemiology, Chair of Genetic Epidemiology, Ludwig-Maximilians-Universität, Munich, Germany; 36 Department of Endocrinology and Diabetology, University Hospital Düsseldorf, Düsseldorf, Germany; 37 School of Population Health, University of South Australia, Adelaide, Australia; 38 Centre for Paediatric Epidemiology and Biostatistics, University College London Institute of Child Health, London, United Kingdom; 39 Center for Medical Systems Biology, Leiden, The Netherlands; 40 Department of Medical Statistics and Bioinformatics, Leiden University Medical Center, Leiden, The Netherlands; 41 Department of Gerontology and Geriatrics, Leiden University Medical Center, Leiden, The Netherlands; 42 Unit of Chronic Disease Epidemiology and Prevention, National Institute for Health and Welfare, Helsinki, Finland; 43 Bioinformatics and Biostatistics Support Hub (B/BASH), University of Leicester, Leicester, United Kingdom; 44 Deutsches Herzzentrum München, Technische Universität München, Munich, Germany; 45 DZHK German Center for Cardiovascular Research, Partner Site Munich, Munich, Germany; 46 The Department of Public Health, University of Helsinki, Helsinki, Finland; 47 Genetic Epidemiology Group, Department of Health Sciences, University of Leicester, Leicester, United Kingdom; 48 National Institute for Health Research (NIHR) Leicester Respiratory Biomedical Research Unit, Glenfield Hospital, Leicester, United Kingdom; 49 Department of Medical Sciences, Uppsala University, Akademiska Sjukhuset, Uppsala, Sweden; 50 Broad Institute of Harvard and MIT, Cambridge, Massachusetts, United States of America; 51 Institute of Molecular and Cell Biology, University of Tartu, Tartu, Estonia; 52 Department of General Practice and Primary Health Care, University of Helsinki, Helsinki, Finland; 53 Folkhalsan Research Center, Helsinki, Finland; 54 Vasa Central Hospital, Vasa, Finland; 55 Department of Health Promotion and Chronic Disease Prevention, National Institute for Health and Welfare, Helsinki, Finland; 56 Department of Chronic Disease Prevention, National Institute for Health and Welfare, Turku, Finland; 57 Research Center of Applied and Preventive Cardiovascular Medicine, University of Turku, Turku, Finland; 58 Department of Clinical Physiology and Nuclear Medicine, University of Turku and Turku University Hospital, Turku, Finland; 59 EMGO Institute for Health and Care Research, VU University & VU University Medical Center, Amsterdam, The Netherlands; 60 Faculty of Medicine, University of Iceland, Reykjavik, Iceland; 61 Wellcome Trust Sanger Institute, Hinxton, Cambridge, United Kingdom; 62 Deparment of Genomics of Common Disease, School of Public Health, Imperial College London, London, United Kingdom; 63 Oxford National Institute for Health Research Biomedical Research Centre, Churchill Hospital, Oxford, United Kingdom; 64 Department of Biostatistics, University of Liverpool, Liverpool, United Kingdom; 65 Department of Molecular and Clinical Pharmacology, University of Liverpool, Liverpool, United Kingdom; Georgia Institute of Technology, UNITED STATES

## Abstract

Reference panels from the 1000 Genomes (1000G) Project Consortium provide near complete coverage of common and low-frequency genetic variation with minor allele frequency ≥0.5% across European ancestry populations. Within the European Network for Genetic and Genomic Epidemiology (ENGAGE) Consortium, we have undertaken the first large-scale meta-analysis of genome-wide association studies (GWAS), supplemented by 1000G imputation, for four quantitative glycaemic and obesity-related traits, in up to 87,048 individuals of European ancestry. We identified two loci for body mass index (BMI) at genome-wide significance, and two for fasting glucose (FG), none of which has been previously reported in larger meta-analysis efforts to combine GWAS of European ancestry. Through conditional analysis, we also detected multiple distinct signals of association mapping to established loci for waist-hip ratio adjusted for BMI (*RSPO3*) and FG (*GCK* and *G6PC2*). The index variant for one association signal at the *G6PC2* locus is a low-frequency coding allele, H177Y, which has recently been demonstrated to have a functional role in glucose regulation. Fine-mapping analyses revealed that the non-coding variants most likely to drive association signals at established and novel loci were enriched for overlap with enhancer elements, which for FG mapped to promoter and transcription factor binding sites in pancreatic islets, in particular. Our study demonstrates that 1000G imputation and genetic fine-mapping of common and low-frequency variant association signals at GWAS loci, integrated with genomic annotation in relevant tissues, can provide insight into the functional and regulatory mechanisms through which their effects on glycaemic and obesity-related traits are mediated.

## Author Summary

Human genetic studies have demonstrated that quantitative human anthropometric and metabolic traits, including body mass index, waist-hip ratio, and plasma concentrations of glucose and insulin, are highly heritable, and are established risk factors for type 2 diabetes and cardiovascular diseases. Although many regions of the genome have been associated with these traits, the specific genes responsible have not yet been identified. By making use of advanced statistical “imputation” techniques applied to more than 87,000 individuals of European ancestry, and publicly available “reference panels” of more than 37 million genetic variants, we have been able to identify novel regions of the genome associated with these glycaemic and obesity-related traits and localise genes within these regions that are most likely to be causal. This improved understanding of the biological mechanisms underlying glycaemic and obesity-related traits is extremely important because it may advance drug development for downstream disease endpoints, ultimately leading to public health benefits.

## Introduction

Quantitative human glycaemic and obesity-related traits, including fasting plasma glucose and insulin (FG and FI), body mass index (BMI), and waist-hip ratio (WHR) are highly heritable [1–5], and are well established risk factors for type 2 diabetes (T2D) and cardiovascular disease [6–10]. Large-scale genome-wide association studies (GWAS) have proved to be extremely successful in the identification of loci harbouring genetic variants contributing to these traits in multiple ethnic groups [11–27]. This process has been facilitated by technical advances in the development of imputation methods [28] that allow evaluation of association with genetic variants not directly assayed on genotyping arrays, but present instead in more dense phased reference panels, such as those made available through the International HapMap Consortium [29,30]. However, the detected loci are typically characterised by common variant association signals, defined by lead SNPs with minor allele frequency (MAF) of at least 5%, which extend over large genomic intervals because of linkage disequilibrium (LD). They also often map to non-coding sequence, making direct biological interpretation of their effect more difficult than for non-synonymous variants. The lead SNPs at GWAS loci are overwhelmingly of modest effect, and together account for only a small proportion (generally less than 5%) of the overall trait variance [17–19,26,27]. As a consequence, there has been limited progress in identifying the genes through which GWAS association signals are mediated, and characterisation of the downstream molecular mechanisms influencing glycaemic and obesity-related traits remains a considerable challenge.

There has been much recent debate as to the role that low frequency and rare variation (MAF<5%) might play in explaining the “missing heritability” of complex human traits [31–33]. It has been hypothesized that some of these variants will have larger effects on traits than common SNPs because they are likely to have arisen as a result of relatively recent mutation events, and thus will have been less subject to purifying selection [34]. Unfortunately, such variation is not well captured by traditional GWAS genotyping arrays, by design, even when supplemented by HapMap imputation [35–37]. However, more recent, higher density reference panels released by the 1000 Genomes (1000G) Project Consortium [38], constructed on the basis of low-pass whole-genome re-sequencing, provide haplotypes at more than 37 million variants for 1,094 individuals from multiple ethnic groups, and facilitate imputation of genetic variation with MAF as low as 0.5% across diverse populations [39–41].

Within the European Network for Genetic and Genomic Epidemiology (ENGAGE) Consortium, we sought to assess the advantages and limitations of high-density imputation for the discovery and fine-mapping of loci for glycaemic and obesity-related traits. We considered 22 European ancestry GWAS (S1 Table), each imputed up to the 1000G “all ancestries” reference panel (Phase 1 interim release, June 2011), in up to (after quality control): 87,048 individuals for BMI; 54,572 individuals for WHR; 46,694 individuals for FG; and 24,245 individuals for FI (S2 and S3 Tables). To account for the impact of overall obesity on central adiposity [18,27] and insulin sensitivity [19], we considered WHR and FI after adjustment for BMI (denoted WHR_adjBMI_ and FI_adjBMI_, respectively). With these high-density imputed data, we aimed to: (i) discover novel signals of association for glycaemic and obesity-related traits, including within established GWAS loci; (ii) evaluate the impact of low-frequency variation to common SNP GWAS signals; (iii) consider the contribution of genetic variants at GWAS loci in explaining trait variance; and (iv) refine the localisation of potential causal variants underlying GWAS association signals and assess the mechanisms through which they impact glycaemic and obesity-related traits.

## Results

### Imputation quality

Within each study, we performed stringent quality control of the genotype scaffold before imputation, minimally including sample and variant call rate and deviation from Hardy-Weinberg equilibrium (S1 Table). Each scaffold was imputed up to the 1000G multi-ethnic reference panel (Phase 1 interim release, June 2011), which includes 762 European ancestry haplotypes, using IMPUTEv2 [42], minimac [39] or specialist in-house software (S1 Table). Making use of the multi-ethnic reference panel, including haplotypes from all ancestry groups, has been demonstrated to reduce error rates and to improve imputation quality, particularly of lower frequency variants [28]. Imputed variants were retained for downstream evaluation and association testing if they passed traditional GWAS quality control thresholds (IMPUTEv2 info score ≥ 0.4; minimac *r* ^2^ ≥ 0.3) [43].

We considered the quality of imputation (as measured by the IMPUTEv2 info score) of variants from the 1000G reference panel in two contributing studies (S4 Table): the 1958 British Birth Cohort from the Wellcome Trust Case Control Consortium (58BC-WTCCC, 2,802 individuals from Great Britain); and the 1966 Northern Finnish Birth Cohort (NFBC1966, 5,276 individuals from Lapland and the Province of Oulu in Northern Finland). In 58BC-WTCCC, 98.8% of common SNPs (MAF≥5%, 6.3 million) and 97.0% of low-frequency variants (0.5%≤MAF<5%, 3.8 million) passed imputation quality control filters, of which 72.9% are not present in HapMap reference panels. However, imputation of rarer variants (0.1%≤MAF<0.5%, 3.4 million) proved less successful in 58BC-WTCCC, with only 80.5% passing quality control filters. The quality of imputation in NFBC1966 was comparable to that observed in 58BC-WTCCC: 99.7% of common SNPs (5.9 million) and 94.4% of low-frequency variants (3.7 million). However, amongst rarer variants, the quality of imputation was noticeably poorer in NFBC1966 (62.8%) than 58BC-WTCCC, presumably reflecting less representation of low-frequency haplotypes from the isolated Northern Finnish population in the 1000G reference panel.

We have demonstrated that high-density imputation provides >90% coverage of low-frequency variants present in the 1000G reference panel in two diverse European ancestry populations. Our study thus enables association testing with more than three million high-quality variants with 0.5%≤MAF<5% that would not have been directly interrogated in previous GWAS of glycaemic and obesity-related traits that have been supplemented by HapMap imputation alone. With the sample sizes available in this study, we have estimated that for any of these variants explaining at least 0.2% of the overall trait variance (i.e. effect size of 0.32 SD units for 1% MAF, and effect size of 0.15 SD units for 5% MAF), we have >99.9% power to detect their association with BMI, WHR, and FG, and >93.9% power to detect their association with FI.

### Discovery of novel loci and new lead SNPs

Within each study, we tested for association of each directly typed and well imputed variant with BMI, WHR_adjBMI_, FG and FI_adjBMI_, separately in males and females, in a linear regression modelling framework (Methods, S2 and S3 Tables). Association summary statistics were then combined across studies in sex-specific and sex-combined fixed-effects meta-analyses for each trait. Variants passing quality control in fewer than 50% of the contributing studies for each trait were excluded from the meta-analysis. Association signals at genome-wide significance (*p*<5x10^-8^) and with lead SNPs independent (*r* ^2^<0.05) and mapping more than 2Mb from those previously reported for the traits were considered novel. By convention, loci were labelled with the name(s) of the gene(s) located closest to the lead SNP, unless more compelling biological candidates mapped nearby (Table 1, S1, S2, S3 and S4 Figs).

**Table 1:** Novel loci for glycaemic and obesity-related traits achieving genome-wide significance (*p*<5x10^-8^).

Trait	Locus	Lead SNP	Chr	Position (b37)	Alleles		EAF	Male meta-analysis				Female-meta-analysis				Sex-combined meta-analysis			
					Effect	Other		Effect (SE)	*p*-value	Cochran’s Q p-value	*N*	Effect (SE)	*p*-value	Cochran’s Q p-value	*N*	Effect (SE)	*p*-value	Sex heterogeneity *p*-value	*N*
**Loci identified in sex-combined meta-analysis**																			
BMI	*ATP2B1*	rs1966714	12	90,671,038	A	G	0.46	0.032 (0.009)	0.00061	0.94	34,613	0.040 (0.009)	6.9x10^-6^	0.70	45,163	0.036 (0.006)	1.9x10^-8^	0.54	79,776
BMI	*AKAP6*	rs12885467	14	33,303,788	C	T	0.49	0.020 (0.008)	0.012	0.59	34,511	0.037 (0.007)	3.0x10^-7^	0.28	45,025	0.029 (0.005)	4.5x10^-8^	0.10	79,536
FG	*RMST*	rs17331697	12	97,868,906	T	C	0.90	0.062 (0.011)	2.9x10^-8^	0.22	17,731	0.036 (0.010)	0.00049	0.43	23,657	0.046 (0.007)	1.3x10^-11^	0.081	46,650
**Loci identified in sex-specific meta-analysis**																			
FG	*EMID2*	rs6947345	7	101,071,933	C	T	0.98	-0.023 (0.034)	0.50	0.30	16,336	0.162 (0.029)	3.8x10^-8^	0.98	22,074	0.082 (0.022)	0.00021	3.7x10^-5^	38,410

We identified two novel loci achieving genome-wide significance for BMI in the sex-combined meta-analysis: *ATP2B1* (rs1966714, MAF = 0.46, *p* = 1.9x10^-8^); and *AKAP6* (rs12885467, MAF = 0.49, *p* = 4.5x10^-8^). For FG, we detected one novel locus in the sex-combined meta-analysis at *RMST* (rs17331697, MAF = 0.10, *p* = 1.3x10^-11^) and a female-specific association at *EMID2* (rs6947345, MAF = 0.017, *p*_MALE_ = 0.50, *p*_FEMALE_ = 3.8x10^-8^). We did not identify any novel loci at genome-wide significance, in either sex-combined or sex-specific analyses, for WHR_adjBMI_ or FI_adjBMI_. We observed no evidence of heterogeneity in sex-specific allelic effects across studies at the lead SNPs at the novel loci (Table 1). With the exception of the sex-specific association signal at *EMID2*, the lead SNPs at all other novel loci were common.

At *AKAP6* and *RMST*, the common lead SNPs were present in HapMap (S5 Fig) but did not achieve genome-wide significance in large-scale European ancestry HapMap imputed meta-analyses conducted by the GIANT Consortium [17] (for BMI in up to 123,865 individuals) and the MAGIC Investigators [16] (for FG in up to 46,186 individuals), despite substantial overlap with cohorts contributing to our study. We have estimated that, amongst individuals contributing to our 1000G imputed meta-analyses for BMI/FG, a maximum of 59%/37% also participated in the previous GIANT and MAGIC studies (S5 Table). At *RMST*, our lead FG SNP approaches genome-wide significance in the MAGIC meta-analysis (*p* = 6.5x10^-6^), and this likely reflects stochastic variation. However, at *AKAP6*, our lead BMI SNP demonstrates only nominal evidence of association (*p* = 0.012) in the GIANT meta-analysis, suggesting that 1000G reference panels have enabled higher quality imputation at this locus. To investigate this assertion further, we compared the quality of imputation of the lead BMI SNP using HapMap and 1000G reference panels in two contributing studies of diverse European ancestry. In 58BC-WTCCC/NFBC1966, there was a marginal improvement in the IMPUTEv2 info score from 0.972/0.939 using reference haplotypes from CEU HapMap to 0.996/0.971 using those from 1000G.

At *ATP2B1*, the common lead SNP was not present in HapMap (S5 Fig). The lead SNP for BMI from the GIANT HapMap imputed meta-analysis [17] was rs2579106, achieving nominal evidence for association (*p* = 6.4x10^-5^) in a reported sample size of 123,864 individuals. This SNP reached near genome-wide significance in our 1000G imputed meta-analysis, despite the smaller sample size (*p* = 3.3x10^-7^, in 86,955 individuals). Furthermore, the HapMap and 1000G lead SNPs are in only modest LD with each other (EUR *r* ^2^ = 0.22). Taken together, these data suggest that the discovery of this novel locus has been due to improved coverage through 1000G imputation, despite the lead SNP being common.

We observed genome-wide significant evidence of association at 34 established loci for glycaemic and obesity-related traits, including *GCKR* with the same lead SNP for both FG and FI (S6 Table). At 29 of these loci, our meta-analysis identified lead SNPs that were different from previous reports in which they were first discovered, of which 23 were not present in HapMap (S7 Table). At 18 of these 29 loci, the new lead SNP was in strong LD (*r* ^2^≥0.8) with that previously reported, and consequently both variants had similar MAF and allelic effect size (S6 Fig). At a further nine of the 29 loci, the new and previously reported lead SNPs were in moderate LD (0.2≤*r* ^2^<0.8) with each other. For these, there was greater difference in MAF and allelic effect size for each pair of variants, but the new lead SNP was common and not consistently less frequent (S6 Fig). At the remaining two loci, the new lead SNPs were not present in HapMap and were in only weak LD with those previously reported (S7 Fig), mapping near *BDNF* for BMI (*r* ^2^ = 0.10) and *RSPO3* for WHR_adjBMI_ (*r* ^2^ = 0.04). At both loci, multiple distinct signals of association have been recently reported by the GIANT Consortium in the largest meta-analyses of BMI and WHR_adjBMI_ in European ancestry individuals genotyped with GWAS arrays, supplemented by imputation up to reference panels from the International HapMap Consortium [29,30], and the Metabochip, in up to 339,224 and 224,459 individuals, respectively [26,27]. At *BDNF*, our new lead SNP (rs4517468) was in moderate LD (*r* ^2^ = 0.31) with the index variant (rs10835210) for the GIANT secondary signal of association for BMI at this locus, suggesting that they represent the same underlying effect on obesity.

At established loci, amongst the 29 lead SNPs identified in our 1000G imputed meta-analysis that were different from the previous reports in which they were discovered, five of them are present on the Metabochip: *NRXN3* (BMI, rs7141420), *SH2B1* (BMI, rs2008514), *MC4R* (BMI, rs663129), *LY86* (WHR_adjBMI_, rs1294437), and *GCKR* (FG/FI_adjBMI_, rs1260326). These variants were thus directly interrogated in the largest European ancestry meta-analyses, to date, of glycaemic and obesity related traits from the GIANT Consortium [26,27] and MAGIC Investigators [19] that made use of this array. At all five of these loci, our new lead SNP is either the same or is in strong LD (EUR *r* ^2^>0.75) with that reported in the trait-equivalent Metabochip effort. Four of these loci (all except *NRXN3*) were densely typed as “fine-mapping” intervals on the array, providing evidence that 1000G imputation has been successful at predicting genotypes at untyped variants in these regions, even though the GWAS scaffolds used in our investigation were comparatively sparse.

### Multiple distinct association signals

**Fig 1:**
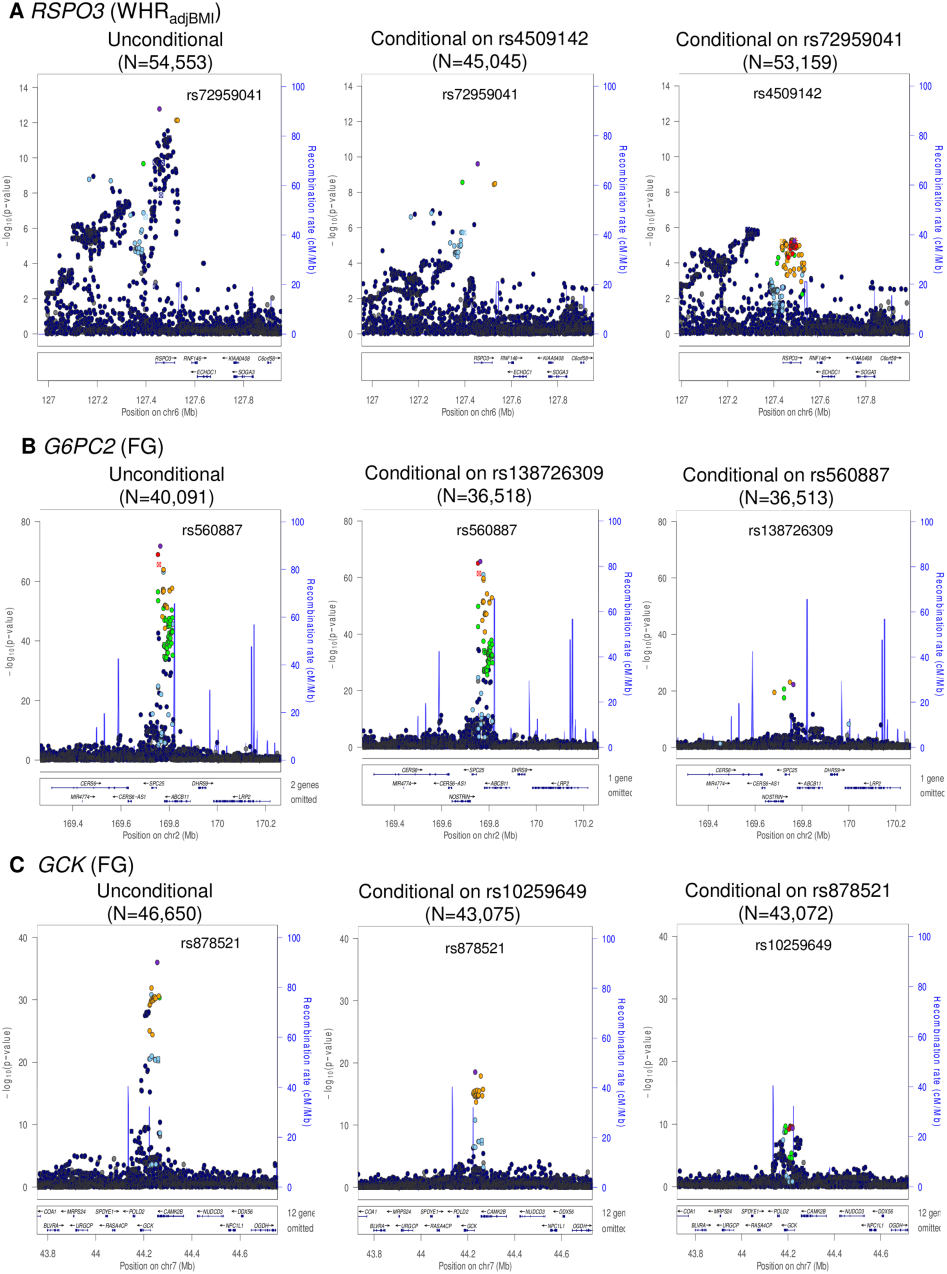
Regional plots of multiple distinct signals at WHR_adjBMI_ locus *RSPO3* (A), FG loci *G6PC2* (B) and *GCK* (C). Regional plots for each locus are displayed from: the unconditional meta-analysis (left); the exact conditional meta-analysis for the primary signal after adjustment for the index variant for the secondary signal (middle); and the exact conditional meta-analysis for the secondary signal after adjustment for the index variant for the primary signal (right). The sample sizes vary due to the availability of the well imputed index SNPs of the primary and secondary signals. Directly genotyped or imputed SNPs are plotted with their association *P* values (on a -log_10_ scale) as a function of genomic position (NCBI Build 37). Estimated recombination rates are plotted to reflect the local LD structure around the associated SNPs and their correlated proxies (according to a blue to red scale from *r* ^*2*^ = 0 to 1, based on pairwise EUR *r* ^*2*^ values from the 1000 Genomes June 2011 release). SNP annotations are as follows: circles, no annotation; downward triangles, nonsynonymous; squares, coding or 3′ UTR; asterisks, TFBScons (in a conserved region predicted to be a transcription factor binding site); squares with an X, MCS44 placental (in a region highly conserved in placental mammals).

We investigated the evidence for multiple distinct association signals in the glycaemic and obesity-related trait loci achieving genome-wide significance in our study (four novel and 34 established) (Table 1 and S6 Table). We undertook approximate conditional analyses, implemented in GCTA [44], to select index SNPs for distinct association signals achieving “locus-wide” significance (*p*_COND_<10^−5^) to reflect the number of uncorrelated variants in a 2Mb window flanking the lead SNP (Methods). We made use of summary statistics from the meta-analysis and genotypes from 58BC-WTCCC and NFBC1966 to approximate the LD between genetic variants (directly typed and well imputed) and hence the correlation in parameter estimates in the joint association model. Reassuringly, the index SNPs and association summary statistics (effect sizes and *p*-values) from the joint model were highly concordant for both reference studies (S8 Table). Finally, we confirmed these GCTA association signals through exact reciprocal conditional analyses by adjustment for genotypes at each index SNP as a covariate in the linear regression model (Methods, Fig 1, Table 2).

**Table 2:** Loci with multiple distinct signals of association with glycaemic and obesity-related traits achieving “locus-wide” significance in conditional analysis (*p*_COND_<10^−5^).

Trait	Locus	Index SNP	Chr	Position (b37)	Alleles		EAF	Unconditional meta-analysis		Conditional meta-analysis		
					Effect	Other		Effect (SE)	*p*-value	Conditioning SNP	Effect (SE)	*p*-value
WHR_adjBMI_	*RSPO3*	rs72959041	6	127,454,893	A	G	0.08	0.11 (0.010)	1.7x10^-13^	rs4509142	0.10 (0.020)	2.5x10^-10^
		rs4509142	6	127,489,001	T	C	0.49	0.04 (0.006)	2.9x10^-12^	rs72959041	0.03 (0.007)	5.8x10^-6^
FG	*G6PC2*	rs560887	2	169,763,148	C	T	0.69	0.09 (0.005)	1.5x10^-72^	rs138726309	0.09 (0.005)	2.2x10^-66^
		rs138726309	2	169,763,262	C	T	0.99	0.18 (0.020)	1.8x10^-18^	rs560887	0.21 (0.020)	5.7x10^-23^
FG	*GCK*	rs878521	7	44,255,643	A	G	0.21	0.06 (0.005)	1.0x10^-36^	rs10259649	0.05 (0.006)	1.3x10^-18^
		rs10259649	7	44,219,705	C	T	0.27	0.05 (0.005)	8.6x10^-29^	rs878521	0.03 (0.005)	4.6x10^-10^

We identified two distinct signals of association for WHR_adjBMI_ mapping to the *RSPO3* locus, indexed by rs72959041 (MAF = 0.079, *p*_COND_ = 2.5x10^-10^) and rs4509142 (MAF = 0.49, *p*_COND_ = 5.8x10^-6^), corresponding to our new lead SNP and that previously reported [18], respectively. More recently, both signals have also been reported by large-scale meta-analyses undertaken by the GIANT Consortium [27]. Our new lead SNP (rs72959041) was reported as the index variant for their secondary association signal at this locus, whilst the index variant for our secondary signal of association (rs4509142) was in strong LD with their lead SNP (rs1936805, *r* ^2^ = 0.67). The GIANT Consortium also identified a third distinct signal of association at this locus, stronger in females than in males, which was not detected in our conditional analyses, and presumably reflects reduced power due to our smaller sample size. We also identified two distinct signals of association for FG each mapping to *GCK* (rs878521, MAF = 0.21, *p*_COND_ = 1.3x10^-18^; rs10259649, MAF = 0.27, *p*_COND_ = 4.6x10^-10^) and *G6PC2* (rs560887, MAF = 0.31, *p*_COND_ = 2.2x10^-66^; rs138726309, MAF = 0.015, *p*_COND_ = 5.7x10^-23^). None of the index variants for these distinct association signals was present in HapMap (S8 Fig), and only rs10259649 in *GCK* was well represented by a tag in that reference panel (rs2908292, *r* ^2^ = 1.00).

### Trait variance explained by novel loci and new lead SNPs

We evaluated the additional heritability of glycaemic and obesity-related traits explained by lead SNPs at novel and established loci after 1000G imputation in 5,276 individuals from NFBC1966 (Methods). For each trait, we calculated the phenotypic variance accounted for by: (i) previously reported lead SNPs at established loci; and (ii) new lead SNPs and index variants for distinct association signals at novel and established loci from the present study. The greatest increment in variance explained was observed for FG, where the novel loci and new lead SNPs after 1000G imputation together account for an increase from 1.9% to 2.3%. We also observed noticeable increments in variance explained after 1000G imputation for WHR_adjBMI_ (from 1.1% to 1.3%) and BMI (3.2% to 3.5%). However, for FI_adjBMI_, only one new lead SNP at an established locus was identified after 1000G imputation, providing a negligible improvement in variance explained (from 0.46% to 0.47%).

### Fine-mapping of novel and established GWAS loci

We sought to take advantage of the improved coverage of common and low-frequency variation offered by 1000G imputation to localise potential causal variants (MAF≥0.5%) for the 42 distinct association signals achieving locus-wide significance in our conditional meta-analyses (two distinct signals of association each at *RSPO3*, *GCK*, and *G6PC2*, one signal of association for both FG and FI_adjBMI_ at the *GCKR* locus, and one signal of association at each of the other 34 novel and established loci). For each distinct signal, we constructed 99% credible sets of variants [45] that together account for 99% probability of driving the association on the basis of the (conditional) meta-analysis (Methods, S9 Table). At the 29 established loci where we identified a new lead SNP after 1000G imputation, the posterior probability of driving the association signal was consistently higher than that for the variant previously reported (S9 Fig). The greatest increases in posterior probability were observed at: *GCKR* (FG/FI_adjBMI_, increase from 2.6%/1.8% to 93.5%/89.6%); *RSPO3* (WHR_adjBMI_, increase from 0.4% to 78.6%); *PROX1* (FG, increase from 13.2% to 76.9%); and *NRXN3* (BMI, increase from 2.5% to 62.2%).

Credible sets are well calibrated for common and low-frequency variants provided that imputation and meta-analysis provides complete coverage of variation with MAF≥0.5% at each locus. Smaller credible sets, in terms of the number of variants they contain, thus correspond to fine-mapping at higher resolution. We considered 99% credible sets containing fewer than 20 variants to be “tractable”, and amenable to follow-up through additional analyses of functional and regulatory annotation (Table 3, S10 Table). The most precise localisation was observed for FG loci including: *MTNR1B* (rs10830963 accounts for more than 99.9% of the probability of driving the association); both distinct signals at *G6PC2* (two variants each, mapping to <15kb interval); and one signal at *GCK* (indexed by rs878521, mapping to <25kb interval). Of the 127 variants reported in these tractable credible sets, 74 (58.3%) were not present in HapMap, and accounted for 42.4% of the probability of driving the association signals. None of the HapMap variants in the tractable credible sets was of low-frequency, compared to 20.8% of those present only in 1000G (S11 Table).

**Table 3:** Association signals for glycaemic and obesity-related traits for which the 99% credible sets contain no more than 20 variants.

Trait	Locus	Index SNP	Chr	Position (b37)	99% credible set					
					Number of variants	Distance	Interval start	Interval stop	Number (%) of variants not in HapMap	Posterior probability of variants not in HapMap
BMI	*SEC16B*	rs539515	1	177,889,025	18	33,234	177,861,357	177,894,591	9 (50.0%)	44.6%
BMI	*GNPDA2*	rs12507026	4	45,181,334	5	10,448	45,175,691	45,186,139	2 (40.0%)	49.0%
BMI	*FAIM2*	rs7132908	12	50,263,148	17	64,525	50,215,905	50,280,430	12 (80.0%)	55.4%
BMI	*NRXN3*	rs7141420	14	79,899,454	17	54,706	79,890,456	79,945,162	5 (29.4%)	13.0%
WHR_adjBMI_	*VEGFA*	rs6905288	6	43,758,873	3	2,431	43,757,896	43,760,327	1 (33.3%)	12.2%
WHR_adjBMI_	*RSPO3*	rs72959041	6	127,454,893	4	140,679	127,389,101	127,529,780	4 (100.0%)	98.9%
FG	*PROX1*	rs340876	1	214,158,132	5	7,161	214,156,514	214,163,675	2 (40.0%)	83.3%
FG	*GCKR*	rs1260326	2	27,730,940	3	21,523	27,730,940	27,752,463	1 (33.3%)	2.6%
FG	*G6PC2*	rs560887	2	169,763,148	2	9,733	169,753,415	169,763,148	0 (0.0%)	0.0%
FG	*G6PC2*	rs138726309	2	169,763,262	2	14,571	169,748,691	169,763,262	2 (100.0%)	99.3%
FG	*GCK*	rs878521	7	44,255,643	2	23,865	44,231,778	44,255,643	1 (50.0%)	18.1%
FG	*GCK*	rs10259649	7	44,219,705	14	70,709	44,183,433	44,254,142	8 (57.1%)	40.5%
FG	*SLC30A8*	rs11558471	8	118,185,733	7	33,132	118,184,783	118,217,915	4 (57.1%)	41.8%
FG	*MTNR1B*	rs10830963	11	92,708,710	1	1	92,708,710	92,708,710	0 (0.0%)	0.0%
FG	*RMST*	rs17331697	12	97,868,906	14	22,285	97,846,621	97,868,906	11 (78.6%)	13.8%
FG (female)	*EMID2*	rs6947345	7	101,071,933	12	97,459	100,995,671	101,931,130	12 (100.0%)	99.0%
FI_adjBMI_	*GCKR*	rs1260326	2	27,730,940	3	21,523	27,730,940	27,752,463	1 (33.3%)	6.5%

The tractable credible sets included coding variants at just three loci implicated in FG: *GCKR*, *SLC30A8*, and the low-frequency association signal at *G6PC2*. The lead SNP mapping to *GCKR* (rs1260326) was the common coding variant L446P, which accounts for 93.5% of the probability of driving the FG association signal, and was present in HapMap. At the *SLC30A8* locus, the probability of driving the association for FG was shared between 7 SNPs, in strong LD with each other, and including the coding variant R325W. This variant was present in HapMap, and was sufficient to explain the association signal of the lead non-coding SNP for FG in conditional analysis (rs11558471, *p* = 3.2x10^-10^, *p*_COND_ = 0.052) at the locus. *SLC30A8* R325W is also the lead SNP for T2D susceptibility at this locus in published European ancestry meta-analyses from the DIAGRAM Consortium [46]. Finally, the low-frequency index SNP for the secondary association signal mapping to *G6PC2* (rs138726309, MAF = 0.015) was the coding variant H177Y, which accounts for 11.2% of the posterior probability of causality at this locus. For this association signal, none of the variants in the 99% credible set was present in HapMap, and thus would have been overlooked without 1000G imputation. This coding variant has recently been implicated in FG homeostasis in a meta-analysis of 33,407 non-diabetic individuals of European ancestry, genotyped with the Illumina exome array, and in agreement with our study, demonstrates a stronger signal of association in conditional analysis after accounting for the lead SNP at the *G6PC2* locus [47].

**Fig 2:**
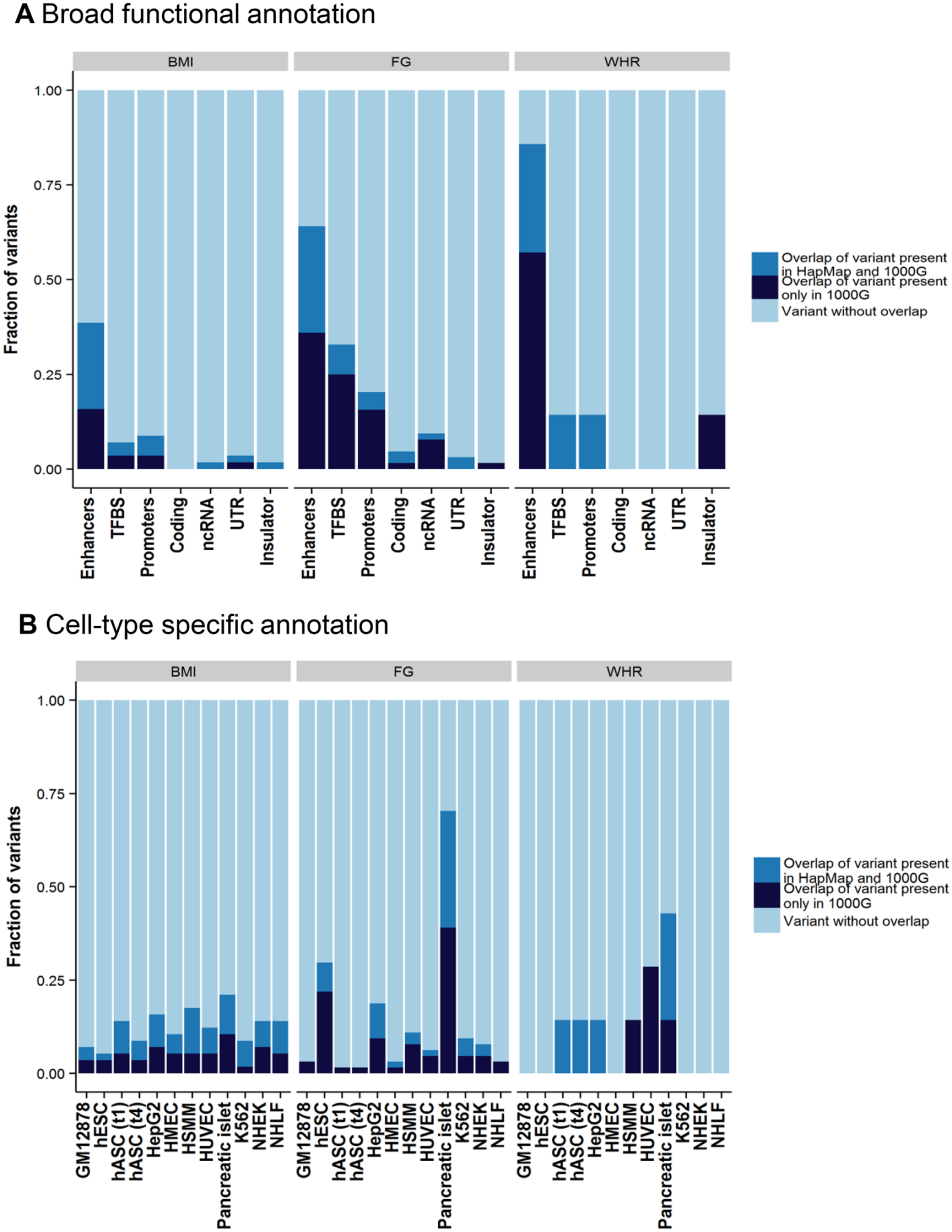
Broad category functional annotation (A) and cell-type specific annotation (B) of credible set variants. On the x-axis is each category of broad functional annotation (A) or cell-type specific annotation (B). The fraction of credible set variants that overlap with each category is shown on y-axis. The overlapping variants are further broken down into either variants that exist in both the 1000 Genomes and HapMap reference panel (green) or those that exist only in the 1000 Genomes reference panel (red). TFBS, transcription factor binding site; ncRNA, non-coding RNA; UTR, untranslated regions; GM12878, lymphoblastoid cell line from European ancestry female; hESC, H1 human embryonic stem cells; hASC(t1), human pre-adipocytes; hASC(t4), mature human adipocytes; HepG2, liver carcinoma cell-line; HMEC, human mammary epithelial cells; HSMM, human skeletal muscle myoblasts; HUVEC, human umbilical vein endothelial cells; K562, human myelogenous leukemia cell-line; NHEK, normal human epidermal keratinocytes; NHLF, normal human lung fibroblasts.

The remaining variants in the tractable credible sets mapped to non-coding sequence. To gain insight into potential regulatory mechanisms through which these variants might impact glycaemic and obesity-related traits, we overlaid each of these credible sets, in turn, with chromatin state calls from eleven cell lines and tissues (Methods). Across all traits, 99% credible set variants were enriched for overlap with enhancer elements (Fig 2). Focussing on FG, variants within the 99% credible set showed significant enrichment (*p*<2.4x10^-3^) for active promoter and transcription factor binding site annotations compared to all others (respectively: 3.8-fold, Fisher's combined *p* = 9.4x10^-5^; and 7.2-fold, Fisher’s combined *p* = 2.1x10^-13^). Over cell types, this enrichment was most prominent in pancreatic islets (Fig 2). More than half of islet-annotated variants are not present in HapMap, and this would not have been observed without 1000G imputation. For example, at the novel FG *RMST* locus, 11 of the 14 variants in the 99% credible set are not present in HapMap, but all overlap active islet chromatin marks (S10 Fig).

## Discussion

Through meta-analysis of 1000G imputed GWAS of glycaemic and obesity-related traits, we have identified two novel loci for BMI at genome-wide significance, and two for FG (including one low-frequency variant association signal that is specific to females). These loci were not reported in larger meta-analysis efforts of European ancestry undertaken by the GIANT Consortium (for BMI) and the MAGIC Investigators (for FG), despite the partial overlap of contributing studies [16–19,26,27]. Improved coverage and quality of imputation for common and low-frequency variation using 1000G reference panels has increased power. We also reported new lead SNPs at 29 established glycaemic and obesity-related trait loci achieving genome-wide significance in our meta-analyses, of which 23 were not present in HapMap, and identified multiple distinct signals of association for WHR_adjBMI_ at *RSPO3* and for FG at *GCK* and *G6PC2*. Taken together, these novel loci, distinct association signals, and new lead SNPs have increased the trait variance explained for glycaemic and obesity-related traits, although the majority of the heritability remains unaccounted for.

Despite more than 90% coverage of low-frequency variation after 1000G imputation, in diverse European ancestry populations, and equivalent power to detect association across the allele frequency spectrum for a fixed proportion of trait variance explained, the new lead SNPs at established and novel GWAS loci are predominantly common. These data argue strongly against the “synthetic association” hypothesis, which posits that common lead SNPs at GWAS loci will often reflect unobserved causal variants of lower frequency and greater effect size [32]. We recognise that our study has insufficient power to detect common or low-frequency association signals of more modest effect (S12 Table). For example, we estimated that the power to detect association in this study, at genome-wide significance, of a variant of 1% MAF, explaining 0.05% of the overall trait variance (effect size of 0.16 SD units), was 88.0% for BMI, but just 42.1% for WHR_adjBMI_, 27.7% for FG, and only 2.6% for FI_adjBMI_. Furthermore, the contribution of rare variants to glycaemic and obesity-related traits cannot be directly investigated with these data because of the low quality imputation for MAF<0.5%, but will require interrogation through deep whole-genome re-sequencing studies in large sample sizes.

We have demonstrated that integration of 1000G imputation, genetic fine-mapping, and genomic annotation, facilitates fine-mapping of GWAS loci for glycaemic and obesity-related traits, and has provided insight into potential functional and regulatory mechanisms through which the effects of these association signals are mediated. In particular, variants in the 99% credible set for the low-frequency association signal mapping to *G6PC2* are completely absent from HapMap, but include H177Y. The glucose lowering allele at this variant has been demonstrated to result in a significant decrease in protein expression mediated through proteasomal degradation, leading to a loss of G6PC2 function [47]. We also demonstrated enrichment for overlap of functional elements with variants in the tractable credible sets mapping to non-coding sequence, in particular enhancers. For FG, additional enrichment was observed across credible set variants mapping to promoter and transcription factor binding sites in pancreatic islets, in particular. Uncovering these types of enrichment is essential for prioritisation of variants for functional follow-up, and can be incorporated in statistical models to elucidate causal alleles. Also, at the level of an individual locus, functional annotation can help point to the underlying molecular mechanism through which the GWAS signal is mediated. At *G6PC2*, for example, the lead SNP, rs560887, in the 99% credible set for the second distinct (non-coding) association signal at this locus (79.5% posterior probability) maps to an enhancer region that is active in pancreatic islets and embryonic stem cells, but repressed in most other cell types. These observations are in agreement with recent reports of clustering of T2D-associated risk variants in islet enhancers [48] and highlights a potential mechanism through which GWAS loci impact glucose homeostasis and disease risk.

Despite the success of traditional GWAS genotyping arrays for the discovery of common variant association signals for complex human traits, because of the structure of LD for variation with MAF>5%, the gold standard approach to directly interrogating lower frequency variation is through re-sequencing studies. However, in agreement with recently published investigations of the contribution of low-frequency variants to a range of phenotypes [47,49–51], our study highlights that effect sizes are modest, and require sample sizes for detection that are financially infeasible through re-sequencing on the scale of the whole genome (or exome). We have demonstrated, in this study, that imputation of existing GWAS scaffolds up to reference panels from the 1000 Genomes Project Consortium [38] enables imputation of more than 90% of low-frequency variants in diverse European populations, at no additional cost other than computation and analyst time. Future GWAS of complex traits in European ancestry populations will be further enhanced by the Haplotype Reference Consortium (www.haplotype-reference-consortium.org). This effort will create a reference panel of more than 60,000 haplotypes from re-sequencing of multiple cohorts, predominantly of European ancestry, enabling high-quality imputation to lower allele frequencies. Phase 3 of the 1000 Genomes Project includes haplotypes from diverse populations from each the five major global ethnicities, and thus would be expected to improve imputation quality over Phase 1 for low-frequency variants in East Asian, South Asian, African and American ancestry groups. The viability of imputation as an approach to recover genotypes at low-frequency variants in GWAS undertaken in populations that are not well represented by the 1000 Genomes Project might require whole-genome re-sequencing of some individuals from the study, in combination with haplotypes from the existing reference panel.

Irrespective of the population under investigation, our study suggests that imputation is unlikely to provide sufficient coverage of variation with MAF<0.5% to enable gene-based testing of rare variants [52]. Imputation is restricted to those rare variants that are present in the reference panel, which are much more likely to be population specific. Furthermore, imputation of rare variants that are present in the reference panel is generally poor, although it is not clear how well calibrated the traditional metrics of quality (such as IMPUTEv2 info score) will be. Thorough investigation of the impact of rare variation on phenotype will thus require re-sequencing, although some success in discovering rare coding variants associated with complex human traits has been achieved through exome array genotyping [47,53–55]. For the time being, arrays that combine an imputation scaffold with direct interrogation of rare coding variation likely offer the most cost-effective approach to assaying variants across the frequency spectrum.

In conclusion, our study has enabled discovery and fine-mapping of novel and established association signals for glycaemic and obesity-related traits, and through integration with genomic data from relevant tissues, has highlighted functional and regulatory processes through which these effects are mediated. Improved understanding of the biological basis of the quantitative human anthropometric and metabolic traits may advance our appreciation of the mechanisms underlying downstream disease endpoints, including T2D and cardiovascular diseases, ultimately leading to personalised treatment approaches, therapeutic development and public health benefits.

## Methods

### Ethics statement

All human research was approved by the relevant institutional review boards, and conducted according to the Declaration of Helsinki. All participants provided written informed consent.

### Studies and samples

We considered 22 population-based and case-control GWAS of European ancestry in up to (after quality control): 87,048 individuals for BMI; 54,572 individuals for WHR_adjBMI_; 46,694 individuals for FG; and 24,245 individuals for FI_adjBMI_. Samples were limited to individuals of at least 18 years of age. Case-control studies were stratified by disease status, with each stratum analysed separately. Full details of study and sample characteristics are provided in S1 Table.

### Genotyping and quality control

Samples were genotyped with a variety of GWAS arrays. Sample and SNP quality control was undertaken within each study. Sample quality control included exclusions on the basis of genome-wide call rate, extreme heterozygosity, sex discordance, cryptic relatedness, and outlying ethnicity. SNP quality control included exclusions on the basis of call rate across samples and extreme deviation from Hardy-Weinberg equilibrium. Non-autosomal SNPs were excluded from imputation and association analysis. SNPs with MAF<1% were also excluded from the genotype scaffold prior to imputation. Full details of the genotyping arrays and quality control protocols employed by each study are summarised in S1 Table.

### Imputation

Within each study, the autosomal GWAS genotype scaffold was imputed up to the 1000 Genomes Project multi-ethnic reference panel (Phase I interim release, June 2011), which was the most up to date available at the time analyses were undertaken. Imputation was performed using IMPUTEv2 [42], minimac [39] or specialist in-house software. Poorly imputed variants (IMPUTE info<0.4; minimac ${\hat{r}}^{2}<0.3$) [43], and those with minor allele count of less than three (under a dosage model) were excluded from downstream association analyses.

### Trait transformations and study-level association analyses

We utilised protocols for obesity-related and glycaemic trait transformations developed by the GIANT Consortium [17,18] and MAGIC Investigators [19]. Full details of trait transformations, trait summary statistics and study-specific covariates are presented in S2 and S3 Tables.

BMI was calculated as the ratio of weight (kg) to squared height (m^2^). BMI was inverse normal transformed separately in males and females. Association of the transformed trait with each variant passing quality control was tested in a linear regression framework under an additive model in the dosage of the minor allele after adjustment for age, age^2^ and study-specific covariates, separately in males and females.

WHR was calculated as the ratio of waist circumference (m) to hip circumference (m). Residuals were obtained after adjustment for age, age^2^, BMI, and study-specific covariates, separately in males and females, and were subsequently inverse-rank normalised. Association of the transformed trait with each variant passing quality control was tested in a linear regression framework under an additive model in the dosage of the minor allele, separately in males and females.

FG was measured in mmol/L. Individuals with a diagnosis of diabetes (type 1 or type 2), diabetes treatment, and/or FG≥7mmol/L, non-fasting state, or pregnancy were excluded. Individuals from case cohorts (with diseases such as stroke and cardiovascular disease) were also excluded if they had undergone hospitalization or blood transfusion in the 2–3 months before measurements were taken. Association of the untransformed trait with each variant passing quality control was tested in a linear regression framework under an additive model in the dosage of the minor allele after adjustment for age, age^2^ and study-specific covariates, separately in males and females.

FI was measured in pmol/L with subsequent natural log transformation. Individuals with a diagnosis of diabetes (type 1 or type 2), diabetes treatment, and/or FG≥7mmol/L, non-fasting state, or pregnancy were excluded. Individuals from case cohorts (with diseases such as stroke and cardiovascular disease) were also excluded if they had undergone hospitalization or blood transfusion in the 2–3 months before measurements were taken. Association of the transformed trait with each variant passing quality control was tested in a linear regression framework under an additive model in the dosage of the minor allele after adjustment for age, age^2^, BMI and study-specific covariates, separately in males and females.

### Meta-analysis

Summary statistics from association testing of variants passing quality control, separately in males and females, were corrected in each study for residual population structure through genomic control [56] where necessary (S2 and S3 Tables). Subsequently, association summary statistics were combined across studies in sex-specific and sex-combined fixed-effects meta-analyses (inverse-variance weighting) for each trait, as implemented in GWAMA [57]. Heterogeneity in allelic effects between males and females for each trait at each variant was assessed by means of an implementation of Cochran’s *Q*-statistic [58] in GWAMA [57]. Variants passing quality control in fewer than 50% of the contributing studies for each trait were excluded from the meta-analysis. After filtering, the total numbers of variants reported for each trait were: 9,953,165 for BMI; 9,954,794 for WHR_adjBMI_; 9,967,162 for FG; and 9,837,044 for FI_adjBMI_. Sex-specific or sex-combined *p*<5x10^-8^ was considered genome-wide significant for each trait. Associated loci are referred to by the name(s) of the nearest gene(s) to lead SNP, unless there are more biologically plausible candidates mapping nearby.

### Approximate conditional analysis

We performed approximate conditioning in established and novel glycaemic and obesity-related trait loci in GCTA [44] on the basis of association summary statistics from the sex-combined meta-analyses after variant filtering. We utilised genotype data from two reference studies to approximate LD between variants in diverse European populations, and hence correlation between parameter estimates in the GCTA-COJO joint regression model: 58BC-WTCCC (2,802 individuals from Great Britain); and NFBC1966 (5,276 individuals from Lapland and the Province of Oulu in Northern Finland). We identified “index” variants to represent each distinct association signal achieving genome-wide significance (*p*<5x10^-8^) in the GCTA-COJO joint regression model for further validation.

### Exact conditional analysis

We performed exact conditional analysis for each locus identified with multiple distinct association signals in GCTA using imputed data from all contributing studies except Rotterdam Study 1 (5,745 individuals). Within each study, we tested for association in the same linear regression framework utilised for unconditional analysis, separately in males and females, but included genotypes at each GCTA index SNP identified at the locus, in turn, as an additional covariate in the model. At each established glycaemic and obesity-related trait locus, we also performed conditioning on the previously reported lead SNP if it differed from that reported in our unconditional meta-analysis. Subsequently, association summary statistics for each signal were combined across studies in sex-specific and sex-combined fixed-effects meta-analyses (inverse-variance weighting) for each trait, as implemented in GWAMA [57].

### Trait variance explained

We estimated the variance explained for each trait using genotype data from NFBC1966 (5,276 individuals) in a multiple linear regression framework. For each trait, we considered two sets of variants: (i) previously reported lead SNPs for established loci; and (ii) new lead SNPs and index variants for multiple distinct association signals in established and novel loci. We tested for association of the trait: (i) with covariates only; and (ii) with covariates and the dosage of the minor allele at each variant. For each set of variants, the trait variance explained was given by the difference in the coefficient of determination (*r* ^2^) between these two regression models.

### Credible set construction

For each distinct signal for each trait, we calculated the posterior probability of driving the association for the *j*th variant, *π*_Cj_, given by

$$\pi_{\text{C}j}=\frac{\Lambda_{j}}{\sum_{k}\Lambda_{k}},$$

where the summation is over all variants reported in the (conditional) meta-analysis across the locus. In this expression, *Λ*_*j*_ is the approximate Bayes’ factor [59] for the *j*th variant, given by

$$\Lambda_{j}=\left[\sqrt{\frac{V_{j}}{V_{j}+\omega }}\right]\text{exp}\left[\frac{\omega \beta_{j}^{2}}{2V_{j}\left(V_{j}+\omega \right)}\right],$$

where *β*_*j*_ and *V*_*j*_ denote the allelic effect and corresponding variance from the (conditional) meta-analysis for the association signal. The parameter *ω* denotes the prior variance in allelic effects, taken here to be 0.04 [59]. A 99% credible set was then constructed by: (i) ranking all variants in the locus according to their Bayes’ factor, *Λ*_*j*_; and (ii) including ranked variants until their cumulative posterior probability exceeds 0.99.

### Functional and regulatory annotation

We interrogated coding variants in the 99% credible set for each association signal using Ensembl and HaploReg [60]. Their likely functional consequences were predicted by SIFT [61], PROVEAN [62] and PolyPhen2 [63].

We collected genomic annotation data from several sources. For regulatory state information, we collected sequence reads generated for six assays (H3K4me1, H3K4me3, H3K27ac, H3K27me3, H3K36me3, and CTCF) from 9 ENCODE cell types (GM12878, K562, HepG2, HSMM, HUVEC, NHEK, NHLF, hESC, HMEC) [64], pancreatic islets [65], and adipose stem cells (hASC t1, t4) [66]. Reads were mapped to the human genome reference sequence (hg19) using BWA [67]. Regulatory states for all cell types were called from the aligned reads using ChromHMM [68], assuming 10 states. We then assigned names to the resulting state definitions as follows: active promoter (High H3K4me3, H3K27ac); strong enhancer 1 (H3K4me3, H3K27ac, H3K4me1); strong enhancer 2 (H3K27ac, H3K4me1); weak enhancer (H3K4me1); poised promoter (H3K27me3, H3K4me3, H3K4me1); repressed (H3K27me3); low/no signal; insulator (CTCF); low/no signal; and transcription (H3K36me3). We also obtained transcription factor binding sites (TFBS) established using chromatin immunoprecipitation sequencing. This consisted of data on 147 proteins [64–66].

Finally, we used transcript information from GENCODEv14 [69] to define protein-coding genes, 5’ and 3’ UTR regions, and non-coding genes. For transcripts to be classified as protein-coding, the ‘protein-coding’ tag needed to be set and further filtering for either presence in the conserved coding DNA sequence (CCDS) database or experimentally confirmed mRNA start and end was applied. From this set of transcripts, 5’ UTR, exon, and 3’ UTR regions were defined. For non-coding genes, transcripts labelled as ‘lncRNA‘, ‘miRNA’, ‘snoRNA’ or ‘snRNA’ were used as non-coding genes.

Overlap between the annotations described above and variants in tractable credible sets was determined using bedtools v2.17.0. We defined seven broad functional classes from these annotation data: coding (protein-coding transcripts); ncRNA (non-coding RNA transcripts); UTR (3’ and 5’ UTR regions of coding transcripts); enhancers (strong and weak enhancer elements); promoters (active and poised promoter elements); insulators; and TFBS (sites pooled across all factors). We further used each of the cell line annotations as a distinct category. Each variant was allowed to overlap multiple annotation categories.

For each broad functional class, Fisher’s exact test as implemented in R v3.0.1 (with alternative = “greater”) was used to compare whether the set of credible variants showed a higher fold overlap of this annotation versus all of the others independently. The six resulting *p*-values for each class were then combined using Fisher’s method. With 21 different functional class and trait combinations, a Bonferroni adjusted significance threshold (*p*<2.4x10^-3^) was used.

## Supporting Information

S1 Fig Quantile-quantile plot of up to 9,967,162 single nucleotide polymorphisms (SNPs) from the meta-analysis for (A) BMI, (B) WHR_adjBMI_, (C) FG and (D) FI_adjBMI_. The black dots represent observed *P* values and the grey line represents the expected *P* values under the null distribution. The red dots represent observed *P* values after excluding the previously identified signals described in S7 Table.
(TIFF)

S2 Fig Genome-wide association results from the sex-combined (Manhattan) and sex-specific (Miami) meta-analysis for (A) BMI, (B) WHR_adjBMI_, (C) FG and (D) FI_adjBMI_. The association *P* value (on -log_10_ scale) for each of up to 9,967,162 SNPs (y-axis) is plotted against the genomic position (NCBI Build 37; x-axis). Association signals that reached genome-wide significance (*P* < 5x10^-8^) are shown in green if novel and pink if previously reported.
(PDF)

S3 Fig Regional plots for novel loci associated with BMI or FG identified through (A) sex-combined and (B) sex-specific meta-analyses. Directly genotyped or imputed SNPs are plotted with their meta-analysis *P* values (as -log_10_ values) as a function of genomic position (NCBI Build 37). In each panel, the lead SNP from the meta-analysis is represented by a purple circle. Estimated recombination rates are plotted to reflect the local LD structure around the associated SNPs and their correlated proxies (according to a blue to red scale from *r* ^*2*^ = 0 to 1, based on pairwise EUR *r* ^*2*^ values from the 1000 Genomes June 2011 release). Gene annotations were taken from the UCSC genome browser. SNP annotations are as follows: circles, no annotation; downward triangles, nonsynonymous; squares, coding or 3′ UTR; asterisks, TFBScons (in a conserved region predicted to be a transcription factor binding site); squares with an X, MCS44 placental (in a region highly conserved in placental mammals).
(TIFF)

S4 Fig Forest plots of the associations at novel loci for BMI (A, B) and FG (C, D). For each study, sex (m, f) and sample size are displayed after the study name. Box size is proportionate to the sample size.
(PDF)

S5 Fig Regional plots for novel loci at or near (A) *ATP2B1*, (B) *AKAP6*, (C) *RMST* and (D) *EMID2* comparing the SNP coverage between 1000 Genomes imputed and HapMap imputed SNPs. For each of the novel signals, all the SNPs imputed up to the 1000 Genomes reference panel (left) or only those present in the HapMap panel (right) are plotted with their meta-analysis *P* values (as -log_10_ values) as a function of genomic position (NCBI Build 37). In both plots, the lead SNP in HapMap panel is represented by a purple circle. Estimated recombination rates are plotted to reflect the local LD structure around the associated SNPs and their proxies (according to a blue to red scale from *r* ^2^ = 0 to 1, based on pairwise *r* ^2^ values from the 1000 Genomes June 2011 release EUR). SNP annotations are as follows: circles, no annotation; downward triangles, nonsynonymous; squares, coding or 3′ UTR; asterisks, TFBScons (in a conserved region predicted to be a transcription factor binding site); squares with an X, MCS44 placental (in a region highly conserved in placental mammals).
(PDF)

S6 Fig Comparison of characteristics of previously reported and new lead SNPs at established loci for glycaemic and obesity-related traits. Minor allele frequency (MAF) (A) and effect size (B) of the previously reported lead SNP on the x-axis and the new lead SNP on the y-axis. Details of the SNPs are presented in S7 Table.
(TIFF)

S7 Fig Regional plots for known signals at (A) *BDNF* and (B) *RSPO3* comparing the SNP coverage between 1000 Genomes imputed and HapMap imputed SNPs. For each association signal, all the SNPs imputed up to the 1000 Genomes reference panel (left) or only those present in the HapMap panel (right) are plotted with their conditional meta-analysis *P* values (as -log_10_ values) as a function of genomic position (NCBI Build 37) after adjustment for the other index SNP at the locus. In each plot, the previously reported lead SNP is highlighted by the purple circle. Estimated recombination rates are plotted to reflect the local LD structure around the associated SNPs and their proxies (according to a blue to red scale from *r* ^2^ = 0 to 1, based on pairwise *r* ^2^ values from the 1000 Genomes June 2011 release EUR).
(TIFF)

S8 Fig Regional plots for multiple distinct association signals at *RSPO3* (A, B), *G6PC2* (C, D) and *GCK* (E, F) comparing the SNP coverage between 1000 Genomes imputed SNPs and HapMap imputed SNPs. For each association signal, all the SNPs imputed up to the 1000 Genomes reference panel (left) or only those present in the HapMap panel (right) are plotted with their conditional meta-analysis *P* values (as -log_10_ values) as a function of genomic position (NCBI Build 37) after adjustment for the other index SNP at the locus. In each plot, the lead SNP present in HapMap is represented by a purple circle. Estimated recombination rates are plotted to reflect the local LD structure around the associated SNPs and their proxies (according to a blue to red scale from *r* ^2^ = 0 to 1, based on pairwise *r* ^2^ values from the 1000 Genomes June 2011 release EUR). SNP annotations are as follows: circles, no annotation; downward triangles, nonsynonymous; squares, coding or 3′ UTR; asterisks, TFBScons (in a conserved region predicted to be a transcription factor binding site); squares with an X, MCS44 placental (in a region highly conserved in placental mammals).
(PDF)

S9 Fig Comparison of the posterior probability between previously reported and new lead SNPs at established loci for glycaemic and obesity-related traits. Posterior probability (PP) of the previously reported lead SNP on the x-axis and the new lead SNP on the y-axis. Details of the SNPs are presented in S7 Table.
(TIFF)

S10 Fig Expression and chromatin status of a novel FG-associated locus, *RMST*, in human tissue. (A) Expression data of *RMST* are extracted from the Human Illumina BodyMap 2.0 and reads per kilobase of exon per million reads (RPKMs) are plotted across 17 human tissues. (B) Annotation of *RMST* in islet cells. Transcription factor binding ChIP sites (TFBS) and chromatin states in islet cell lines from various resources are presented (see Methods).
(TIFF)

S1 Table Cohort summary information on sample quality control, genotyping, imputation and statistical method. (PDF)

S2 Table Summary of obesity-related traits in each study. (PDF)

S3 Table Summary of fasting glycaemic traits in each study. (PDF)

S4 Table Comparison of the SNP coverage between 1000 Genomes imputation and HapMap imputation in 1958 British Birth Cohort (a) and 1966 Northern Finnish Birth Cohort (b). (PDF)

S5 Table Overlap of study samples between GIANT, MAGIC and current ENGAGE consortium. (PDF)

S6 Table Established loci for glycaemic and obesity-related traits achieving genome-wide significance (*p*<5x10^-8^). (PDF)

S7 Table Summary of lead SNPs achieving genome-wide significance in established loci for BMI, WHR_adjBMI_, FG and FI_adjBMI_. (PDF)

S8 Table Approximate conditional analysis results for BMI, WHR_adjBMI_, FG and FI_adjBMI_. (PDF)

S9 Table Summary of 99% credible sets at 42 distinct association signals. (PDF)

S10 Table Variants of 99% credible sets containing less than 20 variants driving distinct association signals for BMI, WHR_adjBMI,_ FG and FI _adjBMI_. (PDF)

S11 Table Allele frequency distribution of 99% credible sets with less than 20 variants. (PDF)

S12 Table Power to detect association, at genome-wide significance (*p*<5x10^-8^), with a variant of MAF 1% in the current study. (PDF)
