# Supplementary figures and images for: Discovery and Fine-Mapping of Glycaemic and Obesity-Related Trait Loci Using High-Density Imputation
Source: PLoS Genet. 2015 Jul 1;11(7):e1005230. doi: 10.1371/journal.pgen.1005230 (PMC4488845; doi:10.1371/journal.pgen.1005230)

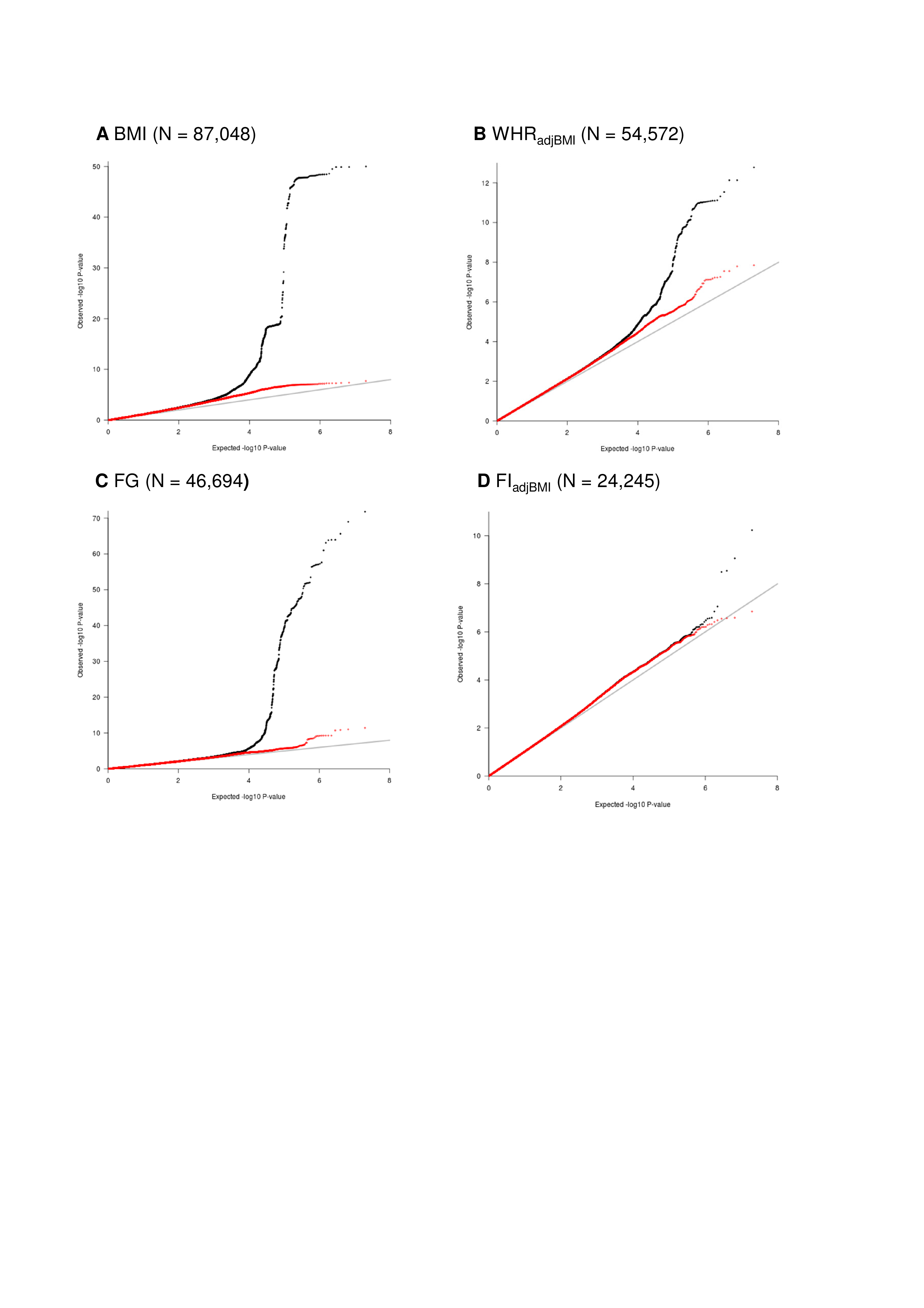

Supplement: S1 Fig — The black dots represent observed P values and the grey line represents the expected P values under the null distribution. The red dots represent observed P values after excluding the previously identified signals described in S7 Table. (TIFF) [file pgen.1005230.s001.tiff]

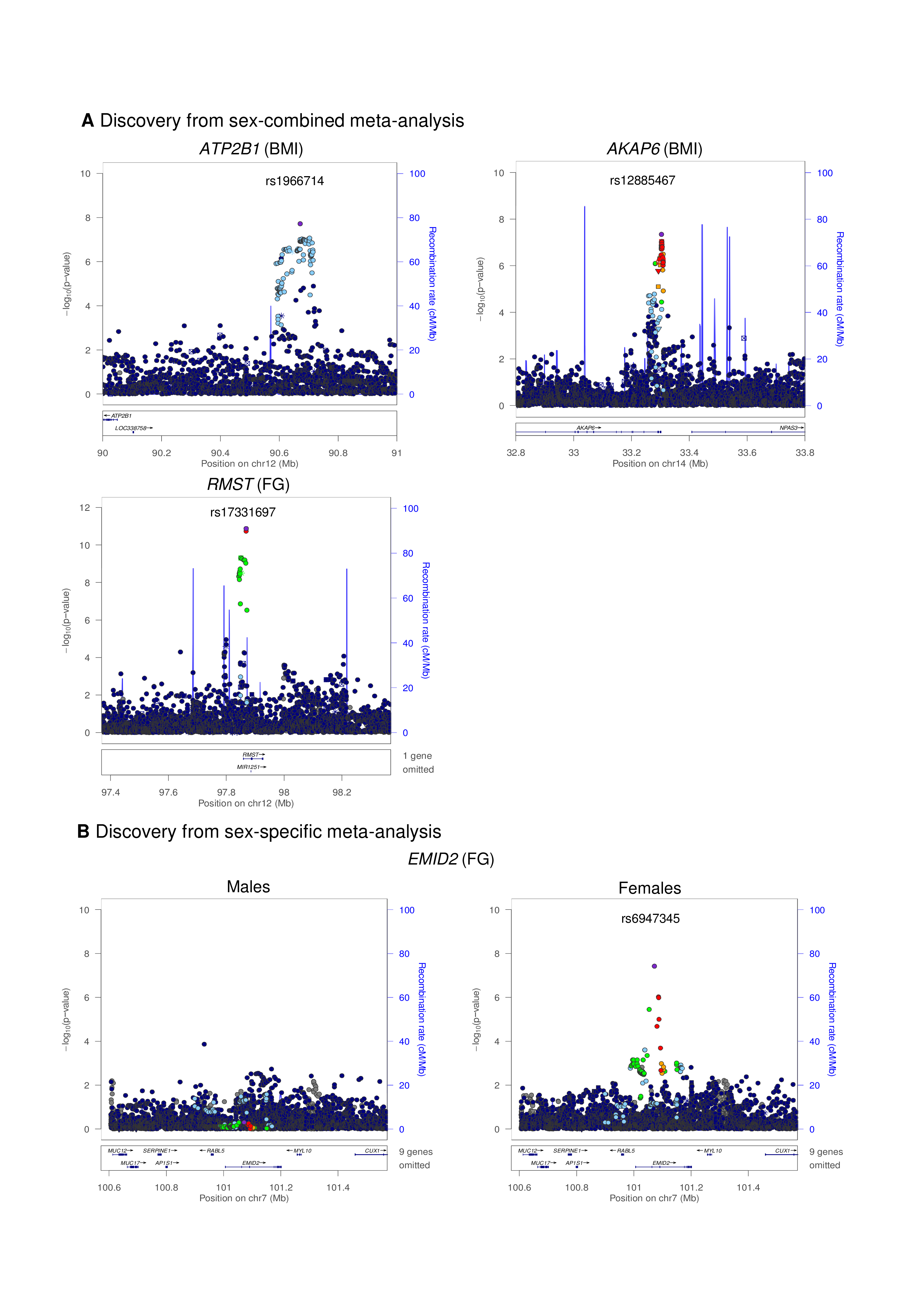

Supplement: S3 Fig — Directly genotyped or imputed SNPs are plotted with their meta-analysis P values (as -log10 values) as a function of genomic position (NCBI Build 37). In each panel, the lead SNP from the meta-analysis is represented by a purple circle. Estimated recombination rates are plotted to reflect the local LD structure around the associated SNPs and their correlated proxies (according to a blue to red scale from r 2 = 0 to 1, based on pairwise EUR r 2 values from the 1000 Genomes June 2011 release). Gene annotations were taken from the UCSC genome browser. SNP annotations are as follows: circles, no annotation; downward triangles, nonsynonymous; squares, coding or 3′ UTR; asterisks, TFBScons (in a conserved region predicted to be a transcription factor binding site); squares with an X, MCS44 placental (in a region highly conserved in placental mammals). (TIFF) [file pgen.1005230.s003.tiff]

## A BMI near *ATP2B1*

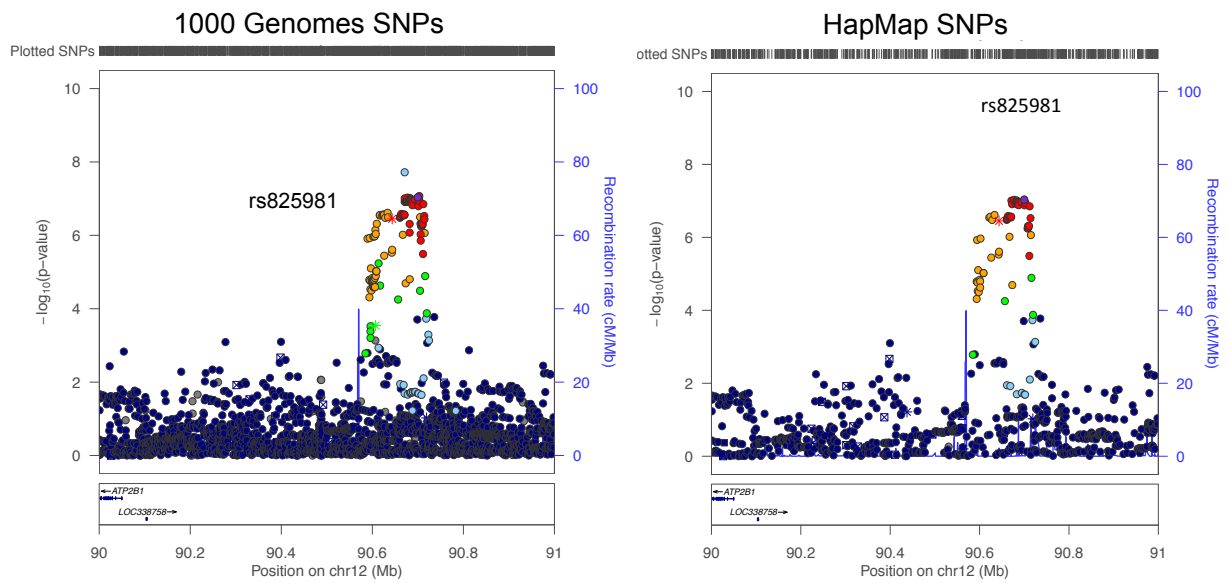

## B BMI *AKAP6*

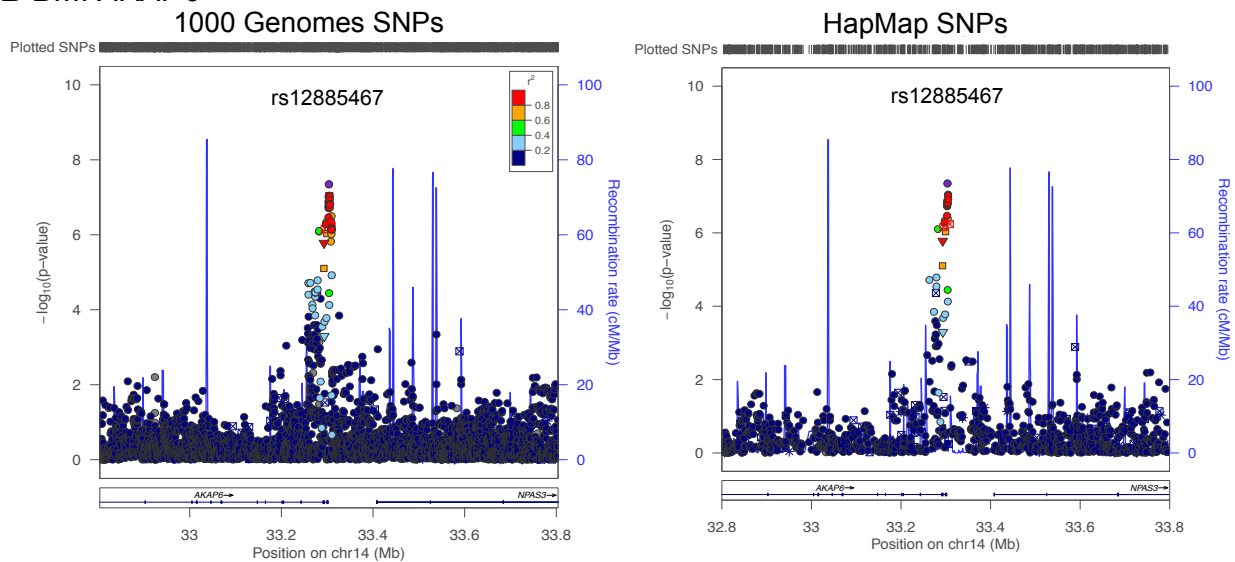

## C FG *RMST*

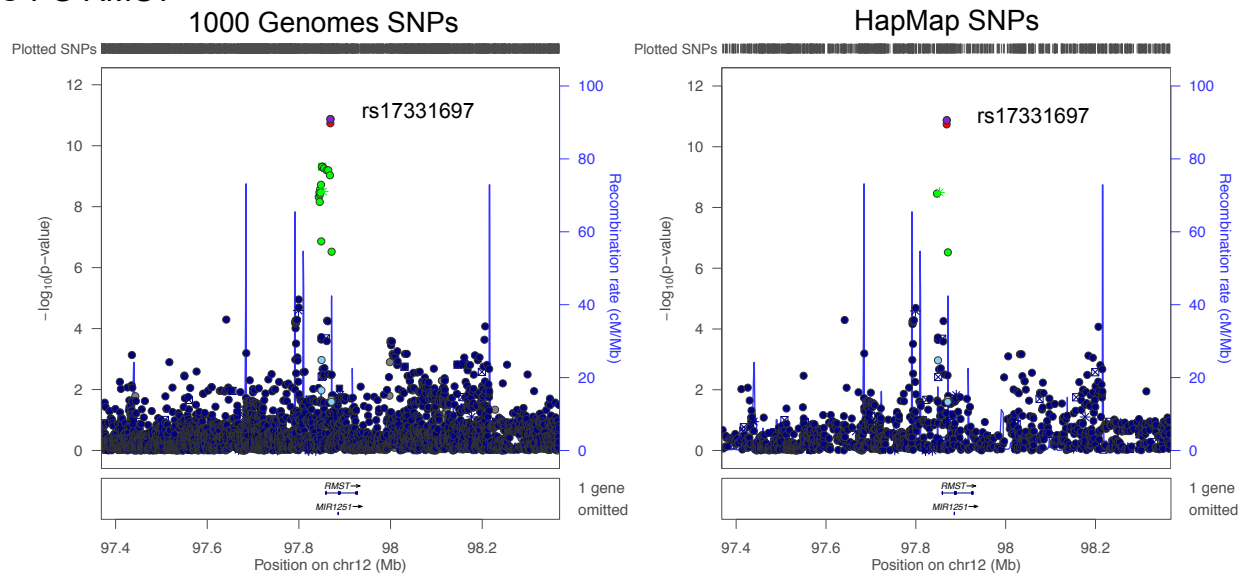

## D FG *EMID2* (females only)

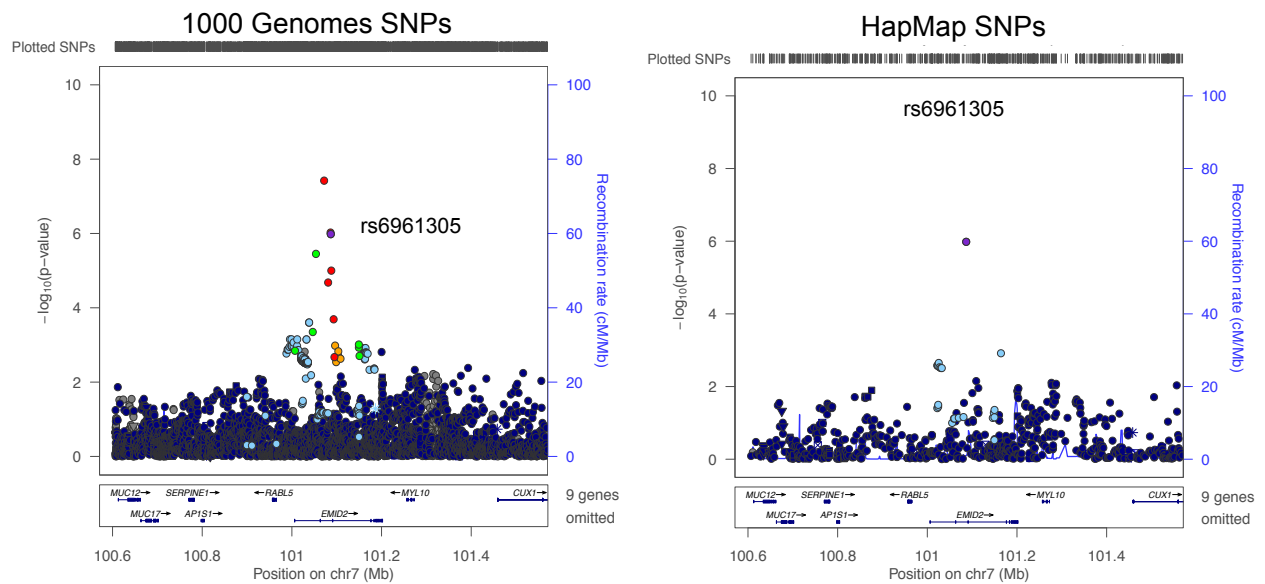

Supplement: S5 Fig — For each of the novel signals, all the SNPs imputed up to the 1000 Genomes reference panel (left) or only those present in the HapMap panel (right) are plotted with their meta-analysis P values (as -log10 values) as a function of genomic position (NCBI Build 37). In both plots, the lead SNP in HapMap panel is represented by a purple circle. Estimated recombination rates are plotted to reflect the local LD structure around the associated SNPs and their proxies (according to a blue to red scale from r 2 = 0 to 1, based on pairwise r 2 values from the 1000 Genomes June 2011 release EUR). SNP annotations are as follows: circles, no annotation; downward triangles, nonsynonymous; squares, coding or 3′ UTR; asterisks, TFBScons (in a conserved region predicted to be a transcription factor binding site); squares with an X, MCS44 placental (in a region highly conserved in placental mammals). (PDF) [file pgen.1005230.s005.pdf]

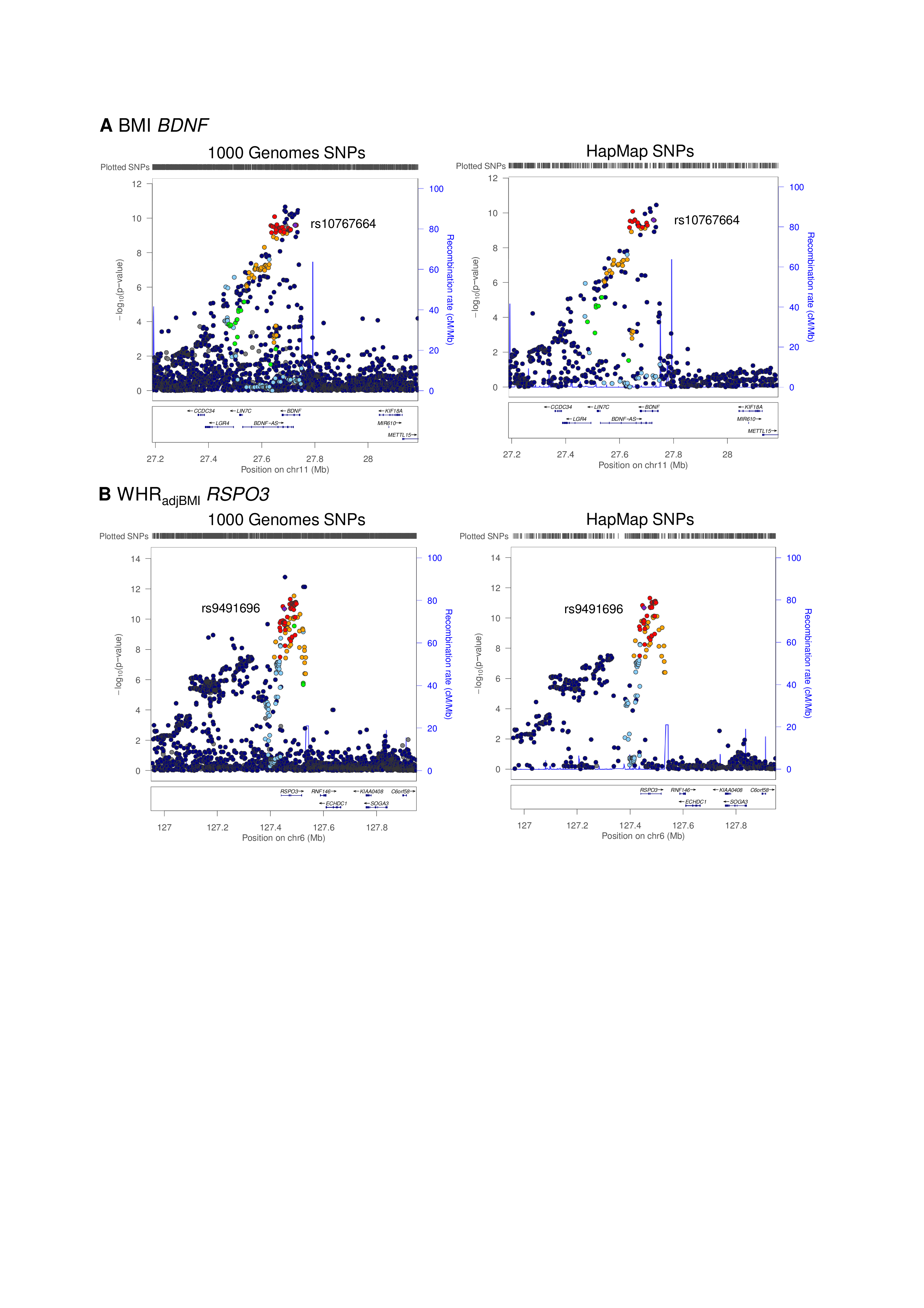

Supplement: S7 Fig — For each association signal, all the SNPs imputed up to the 1000 Genomes reference panel (left) or only those present in the HapMap panel (right) are plotted with their conditional meta-analysis P values (as -log10 values) as a function of genomic position (NCBI Build 37) after adjustment for the other index SNP at the locus. In each plot, the previously reported lead SNP is highlighted by the purple circle. Estimated recombination rates are plotted to reflect the local LD structure around the associated SNPs and their proxies (according to a blue to red scale from r 2 = 0 to 1, based on pairwise r 2 values from the 1000 Genomes June 2011 release EUR). (TIFF) [file pgen.1005230.s007.tiff]

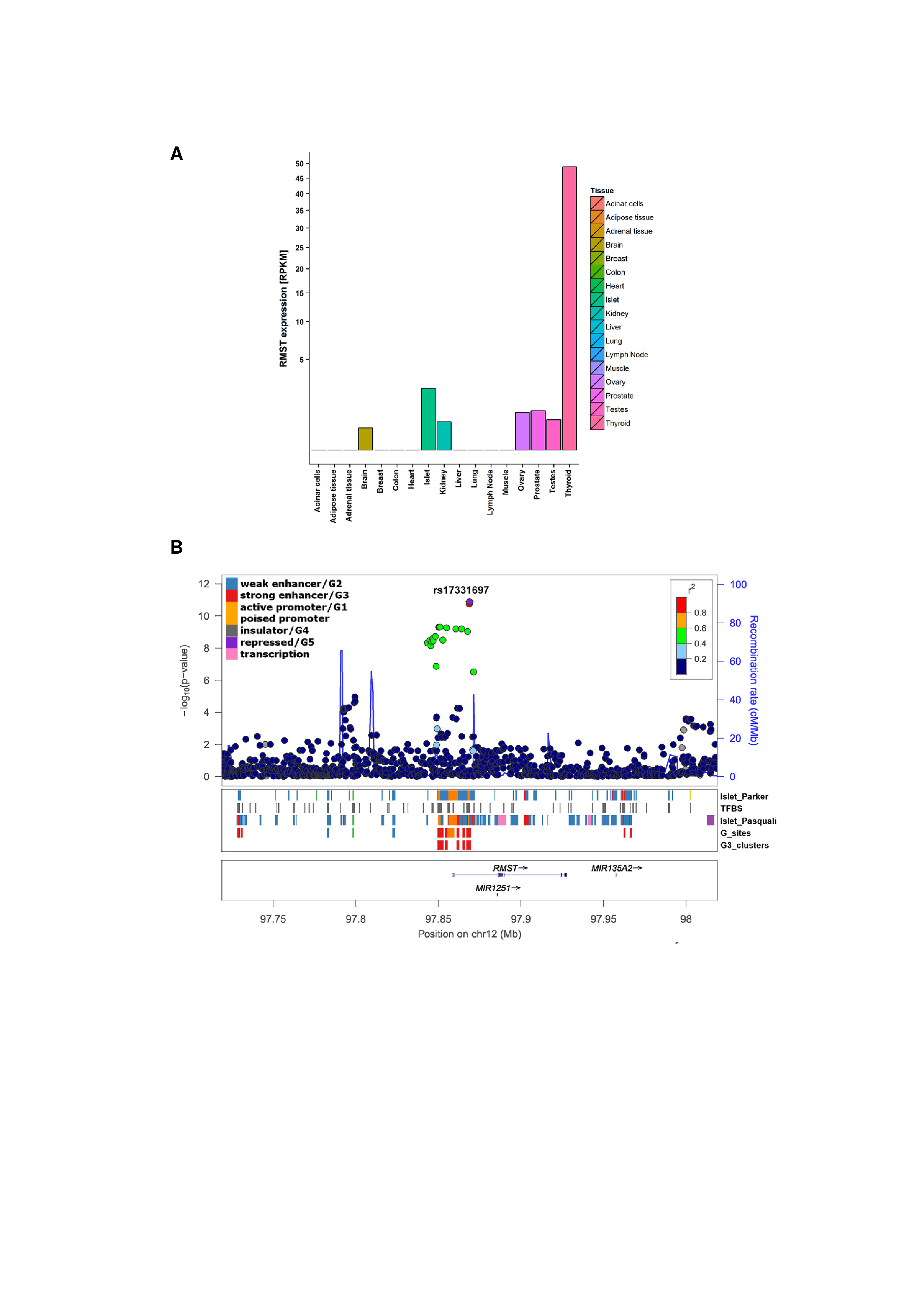

Supplement: S10 Fig — (A) Expression data of RMST are extracted from the Human Illumina BodyMap 2.0 and reads per kilobase of exon per million reads (RPKMs) are plotted across 17 human tissues. (B) Annotation of RMST in islet cells. Transcription factor binding ChIP sites (TFBS) and chromatin states in islet cell lines from various resources are presented (see Methods). (TIFF) [file pgen.1005230.s010.tiff]
