# Supplementary material for: Discovery and Fine-Mapping of Glycaemic and Obesity-Related Trait Loci Using High-Density Imputation
Source: PLoS Genet. 2015 Jul 1;11(7):e1005230. doi: 10.1371/journal.pgen.1005230 (PMC4488845; doi:10.1371/journal.pgen.1005230)

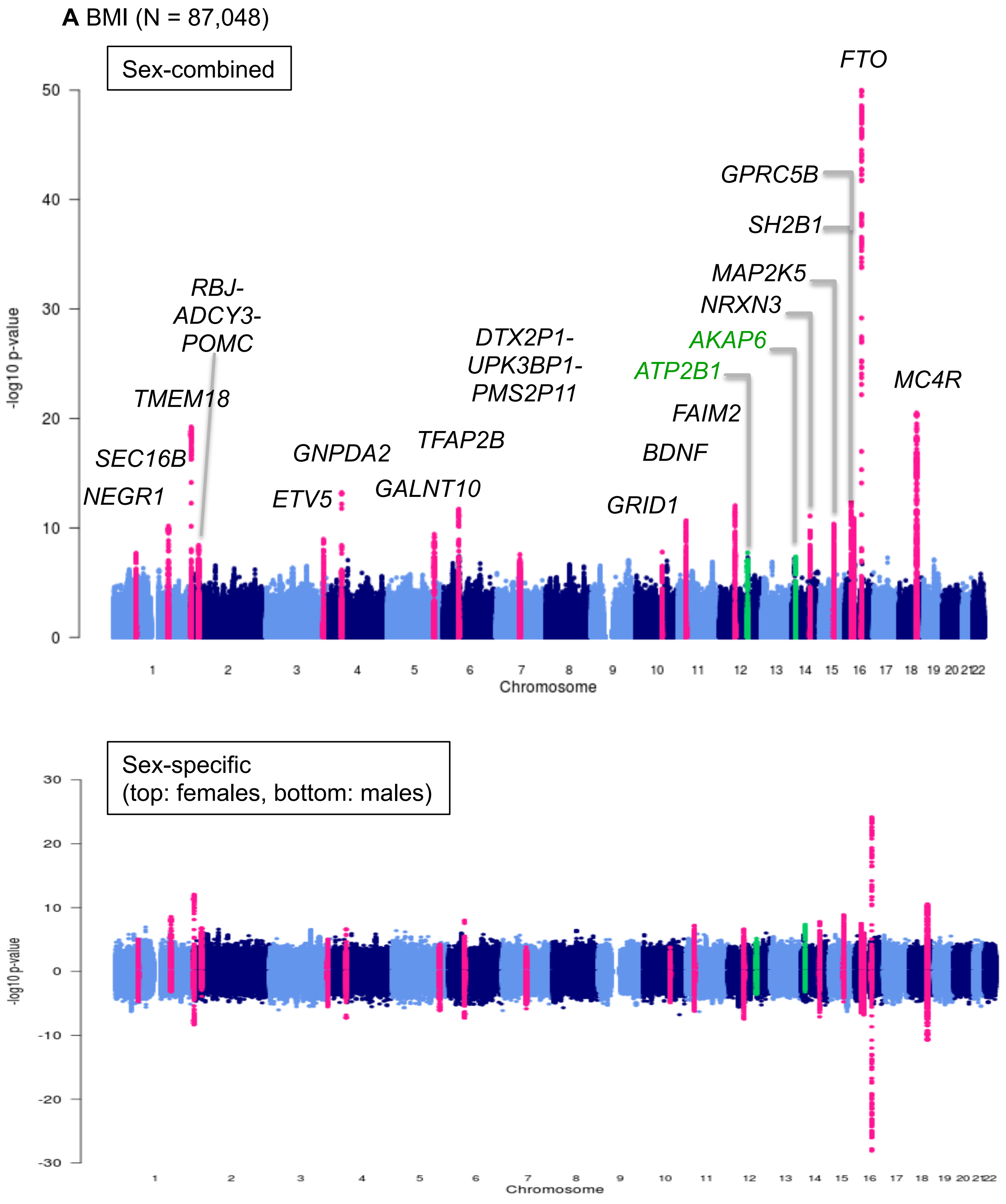
A BMI (N = 87,048)
Sex-combined
FTO
GPRC5B
SH2B1
MAP2K5
NRXN3
AKAP6
ATP2B1
MC4R
RBJ-
ADCY3-
POMC
DTX2P1-
UPK3BP1-
PMS2P11
TMEM18
FAIM2
SEC16B
GNPDA2
TFAP2B
BDNF
NEGR1
ETV5
GALNT10
GRID1
-log10 p-value
Chromosome
Sex-specific
(top: females, bottom: males)
-log10 p-value
Chromosome

B WHR_{adjBMI} (N = 54,572)

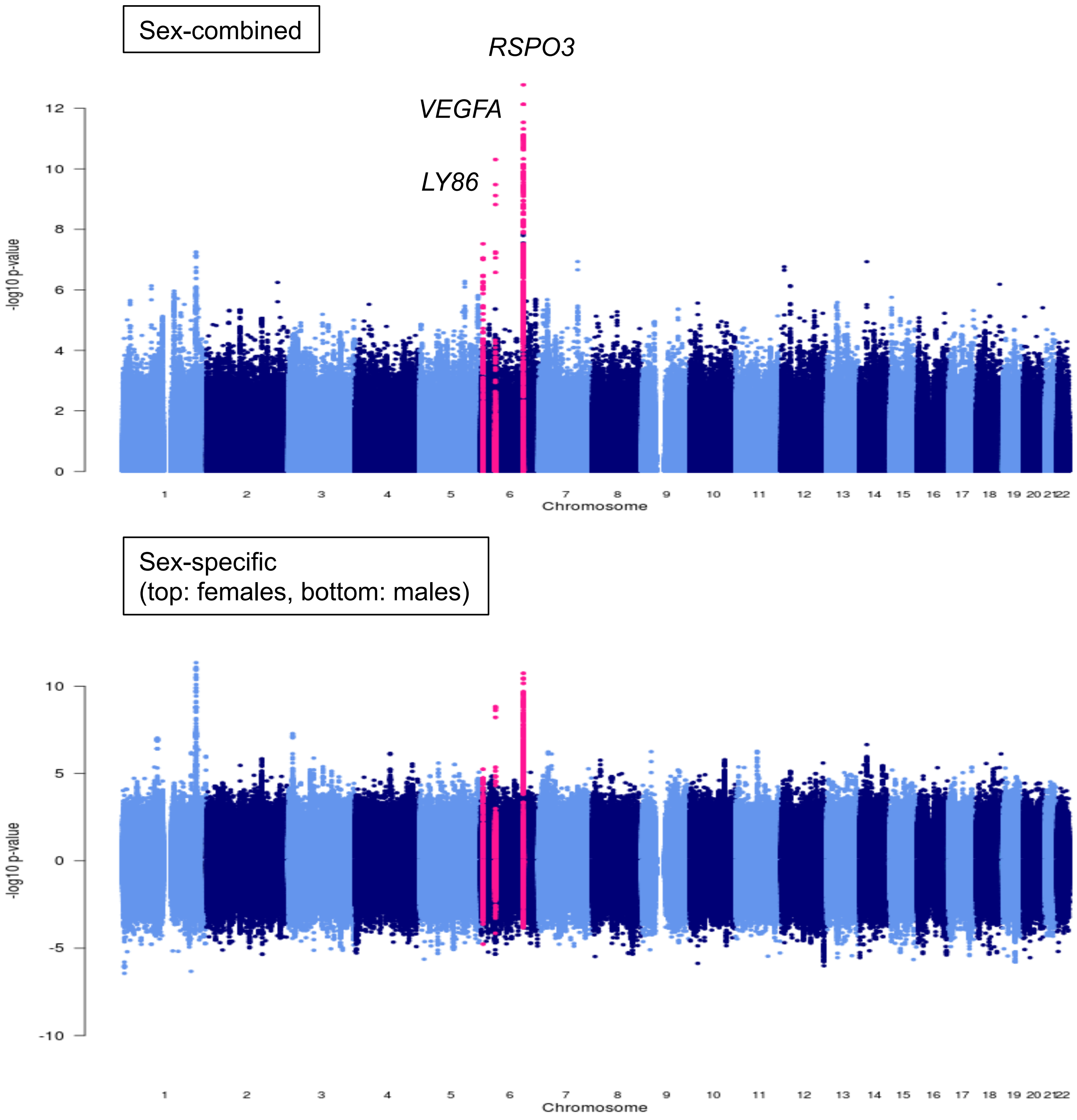

C FG (N = 46,694)

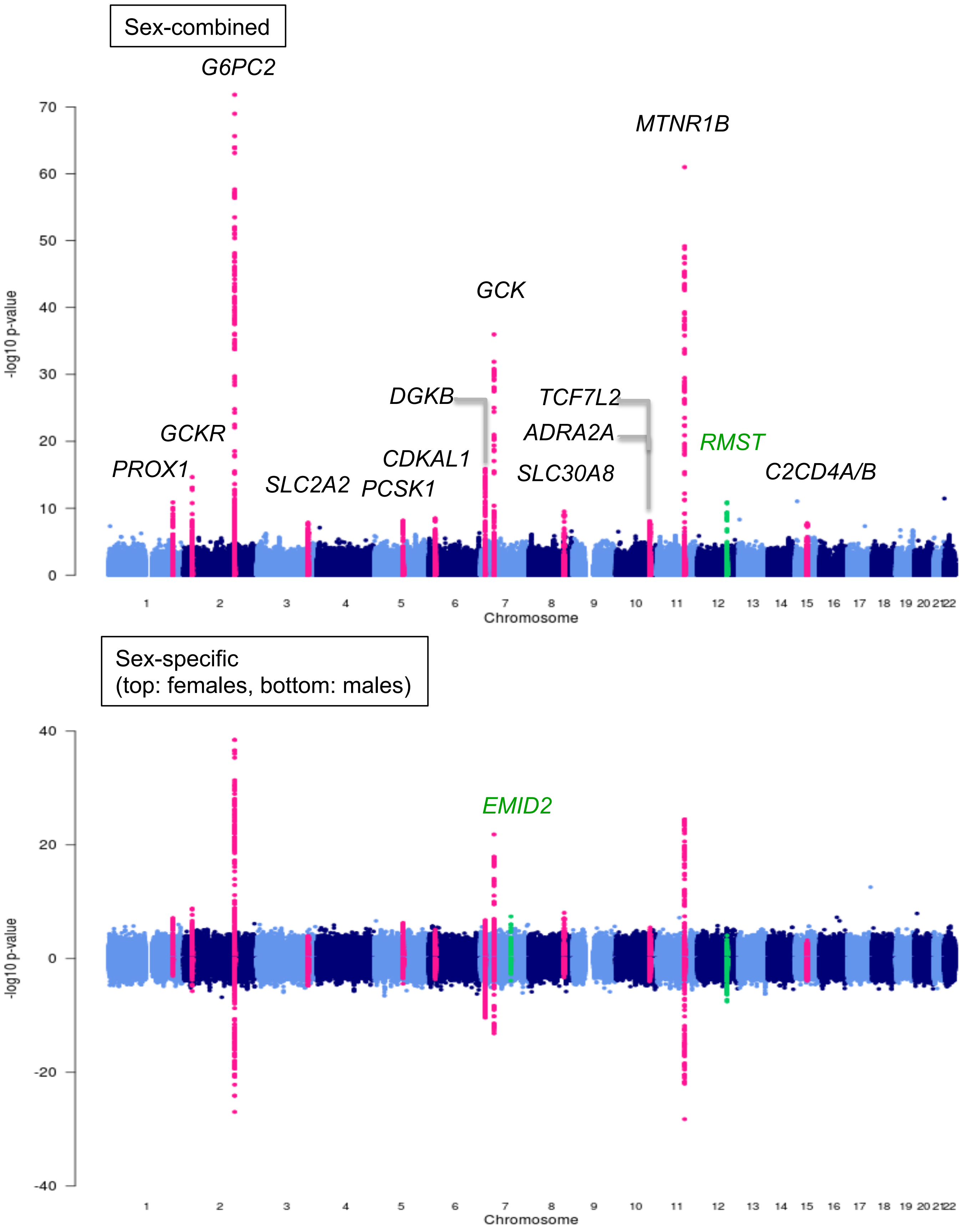

Sex-combined
G6PC2
MTNR1B
GCK
DGKB
TCF7L2
ADRA2A
RMST
GCKR
PROX1
CDKAL1
SLC30A8
C2CD4A/B
SLC2A2
PCSK1
-log10 p-value
0
10
20
30
40
50
60
70
1
2
3
4
5
6
7
8
9
10
11
12
13
14
15
16
17
18
19
20
21
22
Chromosome
Sex-specific
(top: females, bottom: males)
EMID2
-log10 p-value
40
20
0
-20
-40
Chromosome

D FI_{adjBMI} (N = 24,245)

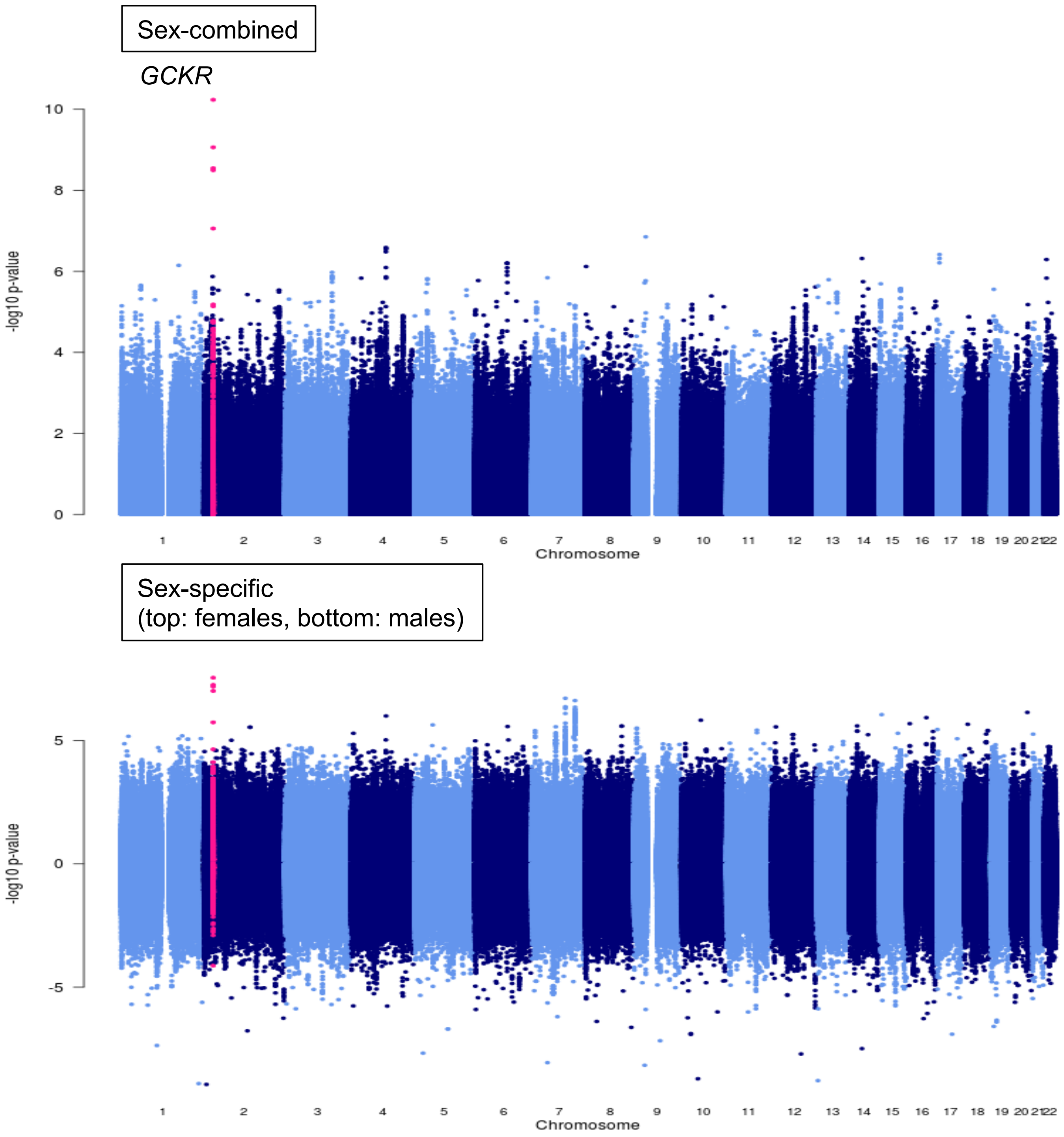

Supplement: S2 Fig — The association P value (on -log10 scale) for each of up to 9,967,162 SNPs (y-axis) is plotted against the genomic position (NCBI Build 37; x-axis). Association signals that reached genome-wide significance (P < 5x10^-8^) are shown in green if novel and pink if previously reported. (PDF) [file pgen.1005230.s002.pdf]
