# Supplementary material for: Discovery and Fine-Mapping of Glycaemic and Obesity-Related Trait Loci Using High-Density Imputation
Source: PLoS Genet. 2015 Jul 1;11(7):e1005230. doi: 10.1371/journal.pgen.1005230 (PMC4488845; doi:10.1371/journal.pgen.1005230)

A BMI: near *ATP2B1* (rs1966714; N=79,776; *P* = 1.9 x 10^{-8}; Cochran's Q *P* = 0.91)

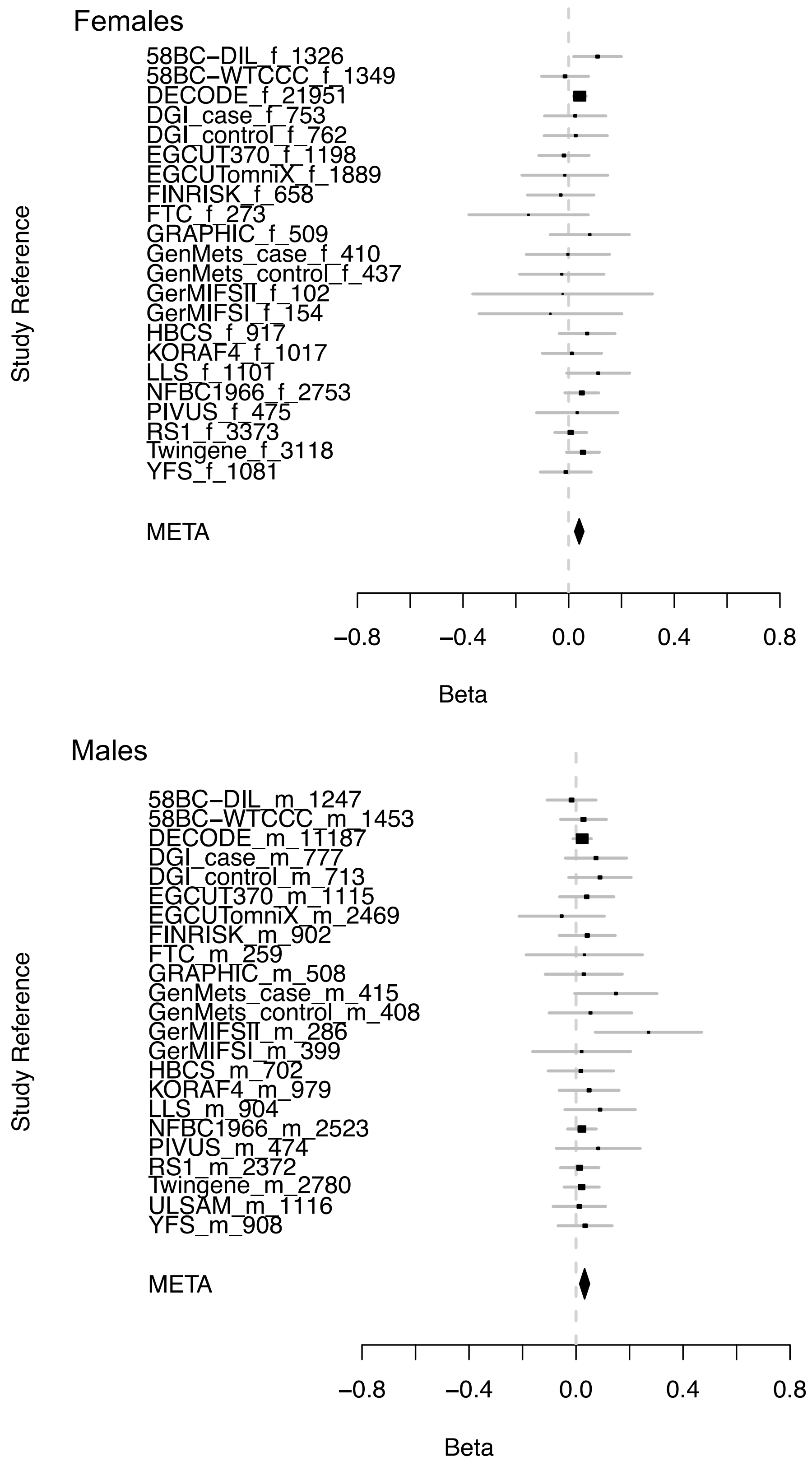

B BMI: *AKAP6* (rs12885467; N=79,536; *P* = 4.5 x 10^{-8}; Cochran's Q *P* = 0.48)

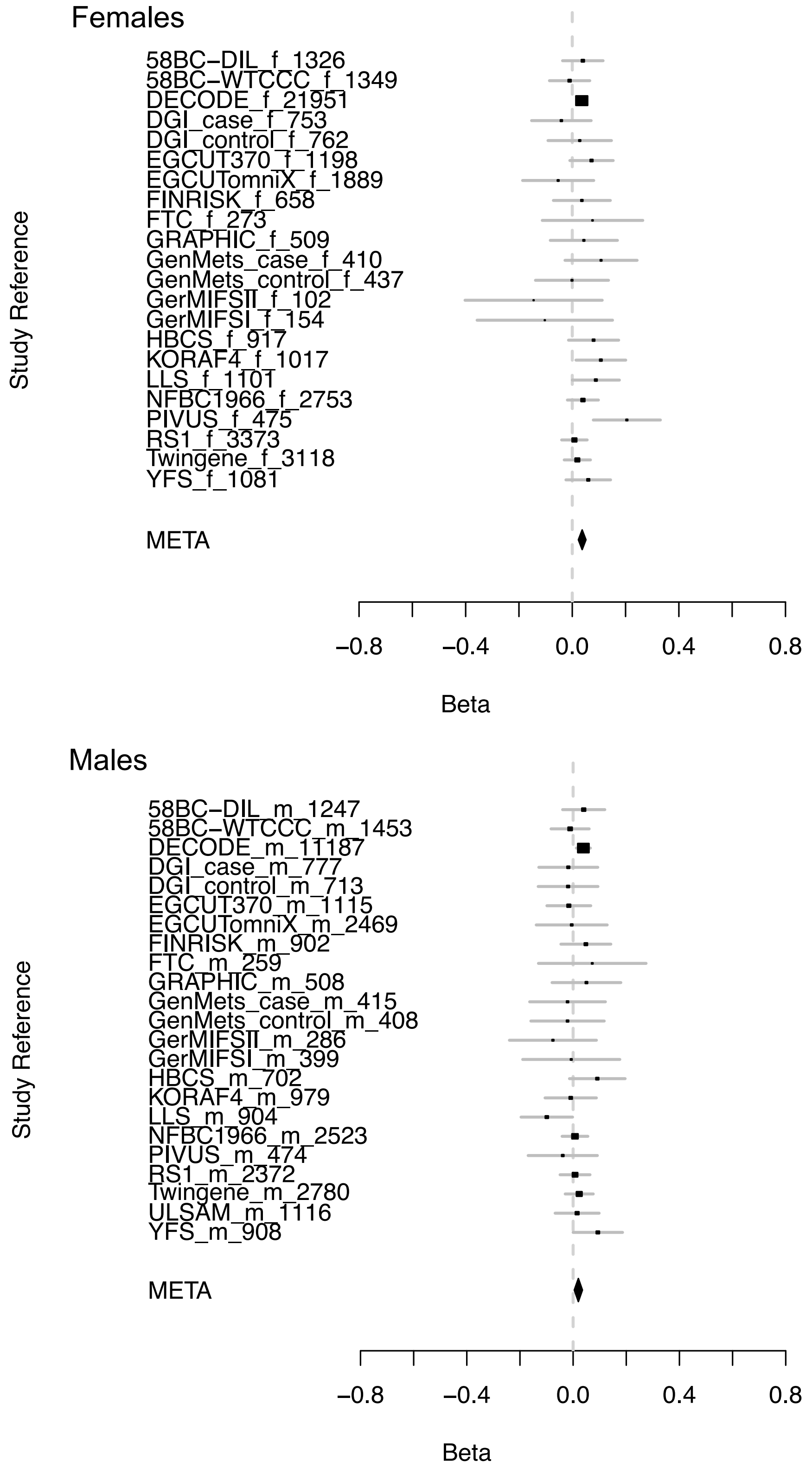

C FG: *RMST* (rs17331697; N=46,650; P = 1.3 x 10^{-11}; Cochran's Q P = 0.23)

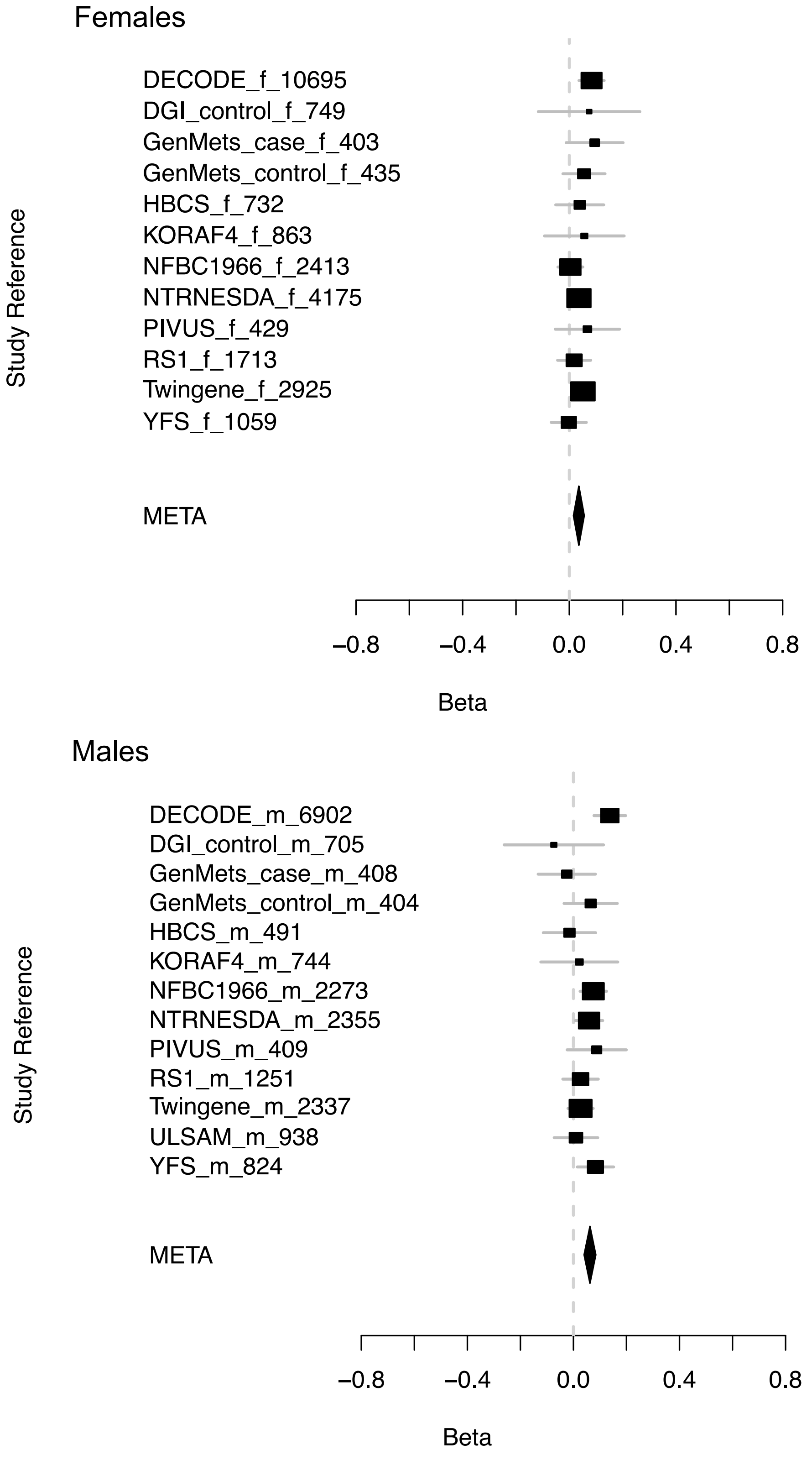

D FG: *EMID2* (rs6947345; N=16,336, P = 0.50 in males; N=22,074, $P = 3.8 \times 10^{-8}$ in females; Cochran's Q P = 0.066; sex-heterogeneity $P = 3.7 \times 10^{-5}$)

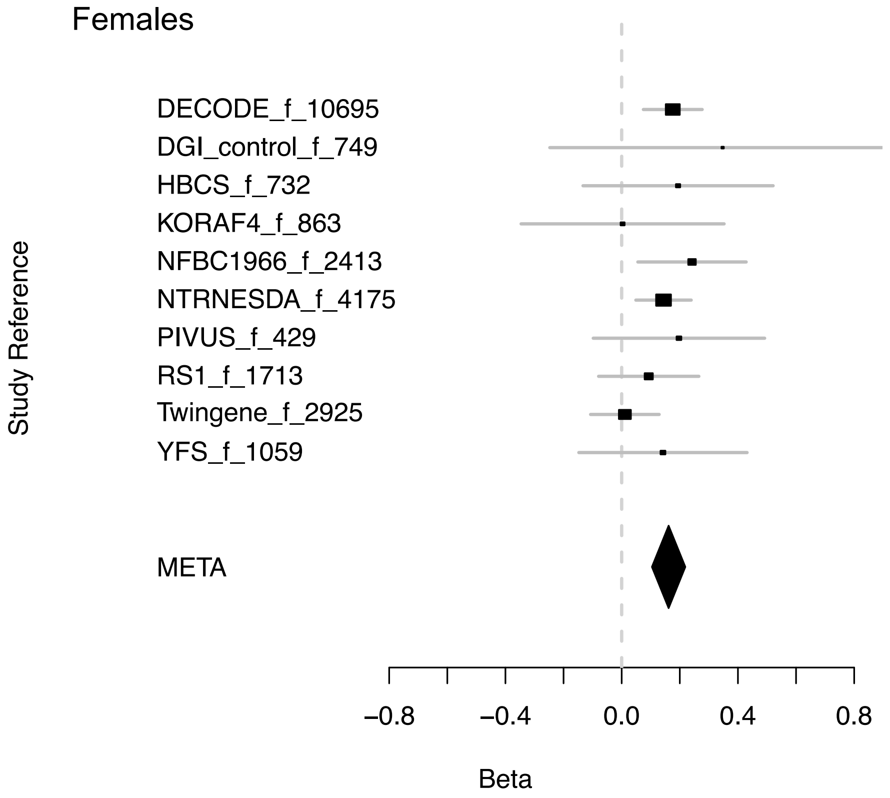

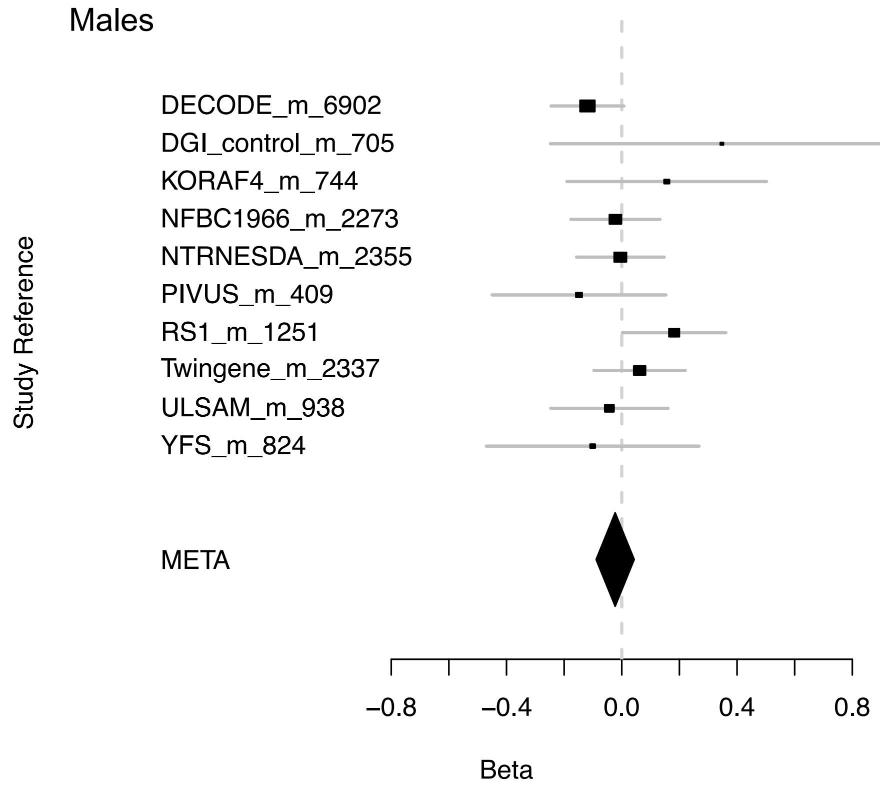

Supplement: S4 Fig — For each study, sex (m, f) and sample size are displayed after the study name. Box size is proportionate to the sample size. (PDF) [file pgen.1005230.s004.pdf]
