# Supplementary material for: Discovery and Fine-Mapping of Glycaemic and Obesity-Related Trait Loci Using High-Density Imputation
Source: PLoS Genet. 2015 Jul 1;11(7):e1005230. doi: 10.1371/journal.pgen.1005230 (PMC4488845; doi:10.1371/journal.pgen.1005230)

A WHR$_{adjBMI}$ *RSPO3* primary signal

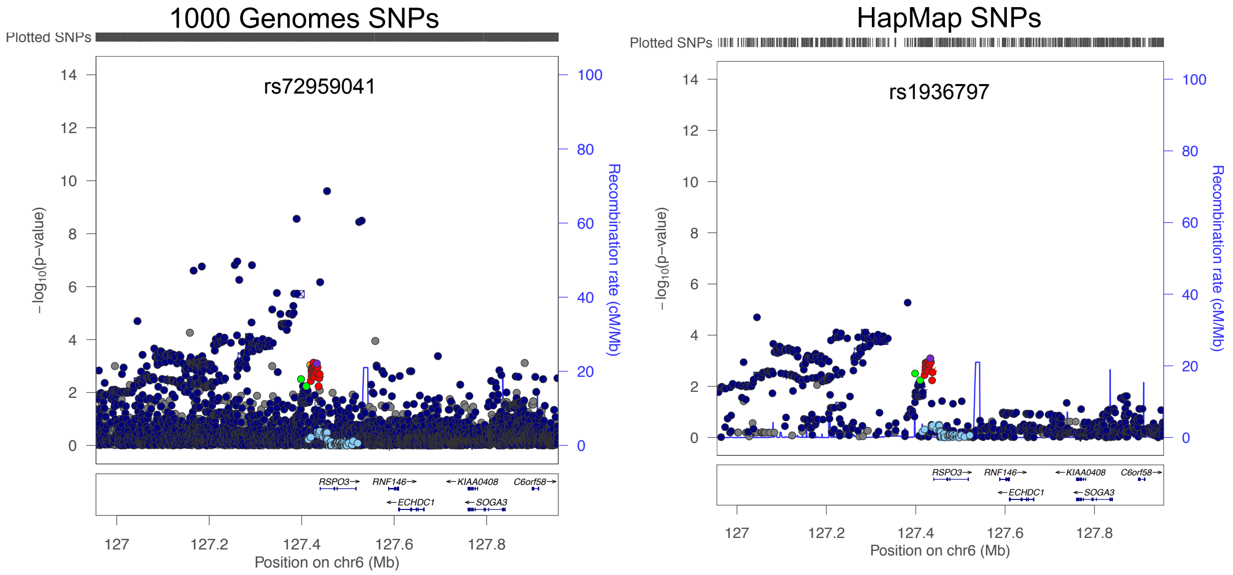

B WHR$_{adjBMI}$ *RSPO3* secondary signal

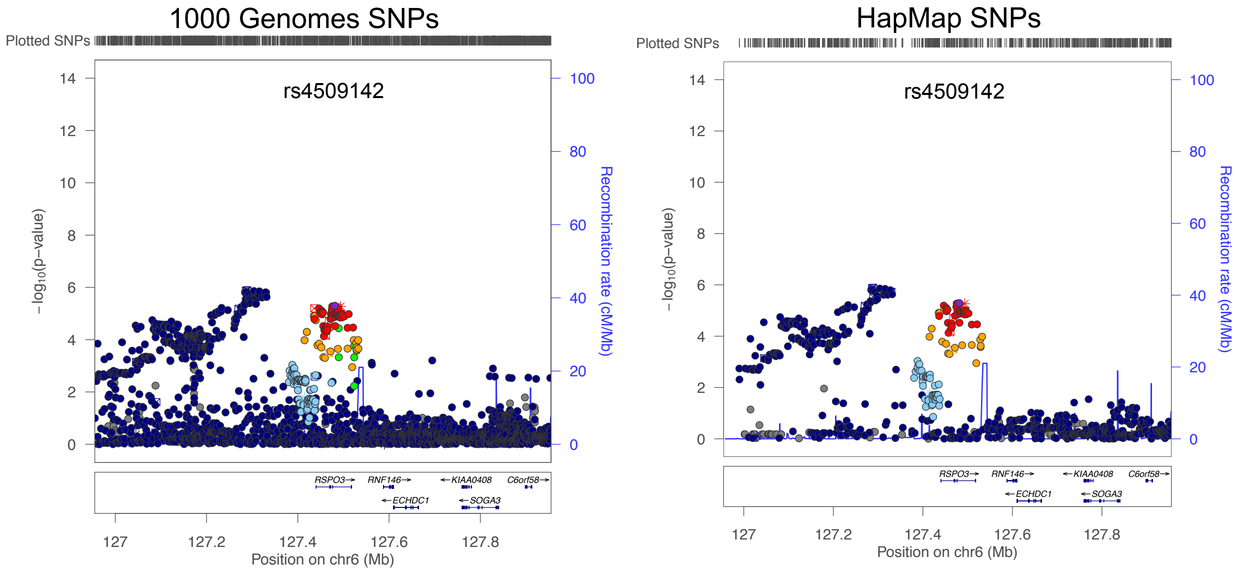

C FG *G6PC2* primary signal

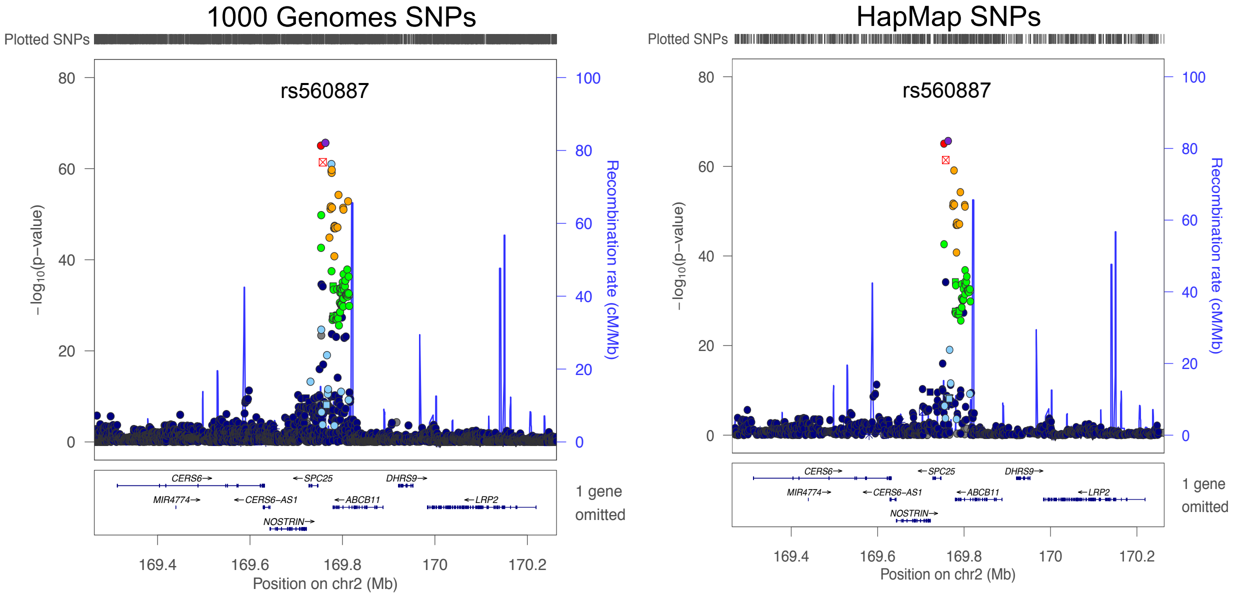

D FG *G6PC2* secondary signal

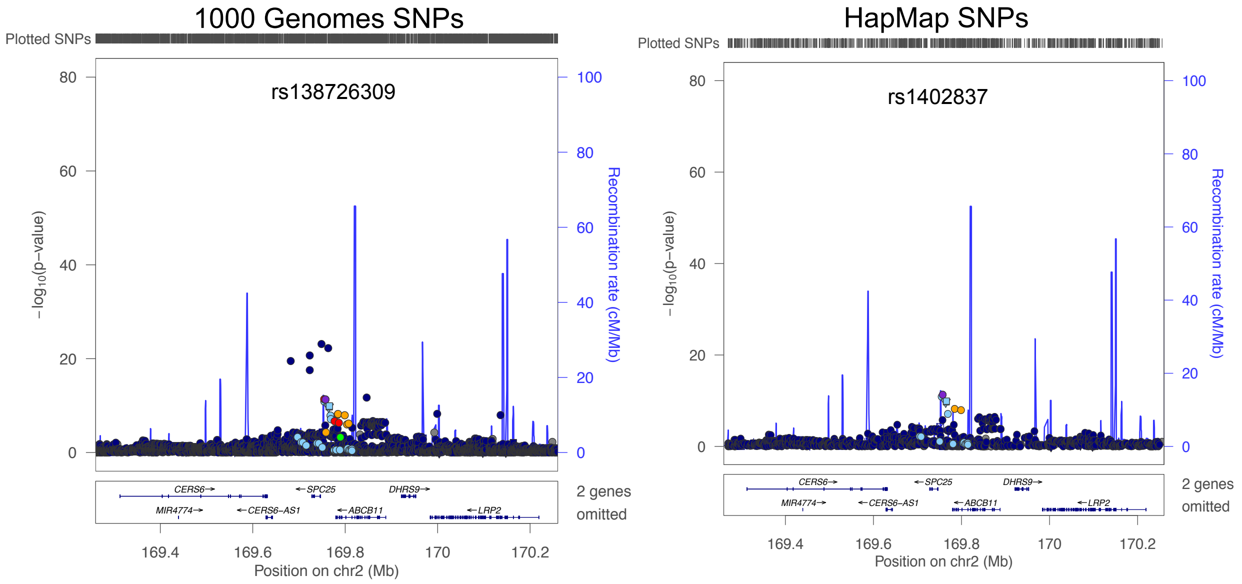

E FG *GCK* primary signal

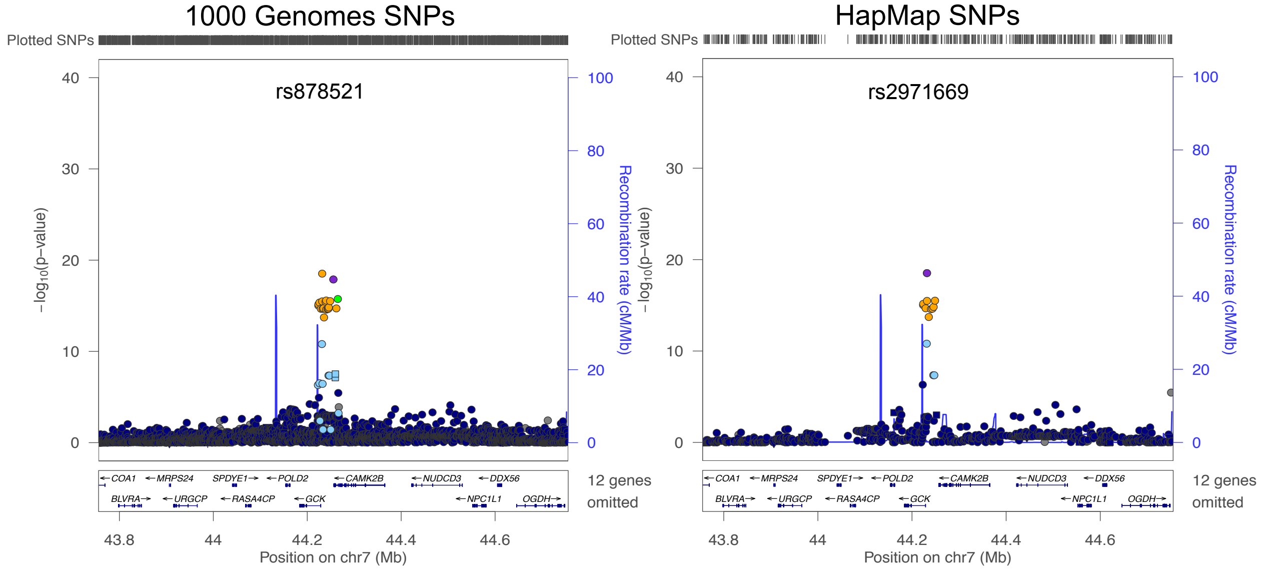

F FG *GCK* secondary signal

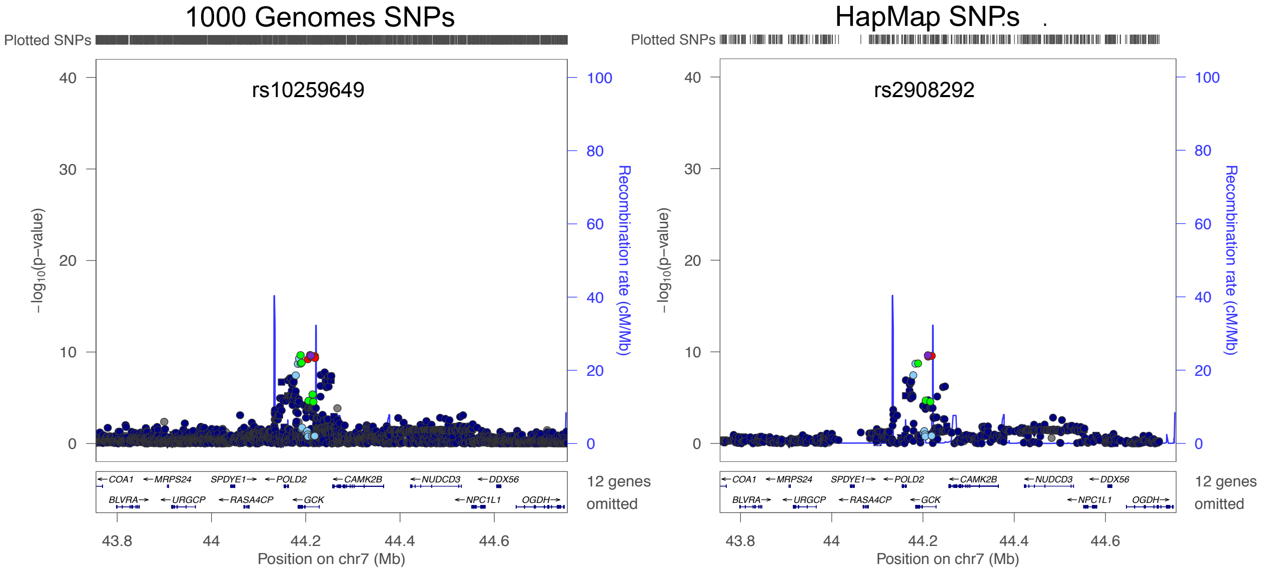

Supplement: S8 Fig — For each association signal, all the SNPs imputed up to the 1000 Genomes reference panel (left) or only those present in the HapMap panel (right) are plotted with their conditional meta-analysis P values (as -log10 values) as a function of genomic position (NCBI Build 37) after adjustment for the other index SNP at the locus. In each plot, the lead SNP present in HapMap is represented by a purple circle. Estimated recombination rates are plotted to reflect the local LD structure around the associated SNPs and their proxies (according to a blue to red scale from r 2 = 0 to 1, based on pairwise r 2 values from the 1000 Genomes June 2011 release EUR). SNP annotations are as follows: circles, no annotation; downward triangles, nonsynonymous; squares, coding or 3′ UTR; asterisks, TFBScons (in a conserved region predicted to be a transcription factor binding site); squares with an X, MCS44 placental (in a region highly conserved in placental mammals). (PDF) [file pgen.1005230.s008.pdf]
