# Supplementary material for: Discovery and Fine-Mapping of Glycaemic and Obesity-Related Trait Loci Using High-Density Imputation
Source: PLoS Genet. 2015 Jul 1;11(7):e1005230. doi: 10.1371/journal.pgen.1005230 (PMC4488845; doi:10.1371/journal.pgen.1005230)
Supplement: S1 Table — (PDF) [file pgen.1005230.s011.pdf]

S1 Table. Cohort summary information on sample quality control, genotyping, imputation and statistical analysis method.

1. Study information			2. Sample				3. Genotyping				4. Imputation	5. Association analysis		
Study	Study type	Ethnic group (country of origin)	Sample size (% males / % females)	Age (mean (SD); males /females)	Sample QC		Genotyping array	SNP QC			Software	Population structure adjustment	Analysis of related individuals	Software
					Call rate	Exclusion criteria		HWE-*p*	Call rate	MAF				
58BC-WTCCC	Population based	European (UK)	2802 (52%/48%)	45.0 (0.0)/ 45.0 (0.0)	95%	Heterozygosity, gender check and relatedness	Affy6.0 & Illumina 1M	1x10^{-6}	95% (99% if MAF<5%)	1%	IMPUTE2	None	No related individuals	SNPTest
58BC-DIL	Population based	European (UK)	2573 (49%/51%)	45.0 (0.0)/ 45.0 (0.0)	95%	Heterozygosity, gender check and relatedness	Illumina HumanHap550	1x10^{-6}	95% (99% if MAF<5%)	1%	MPUTE2	None	No related individuals	SNPTest
deCODE	Population based	European (Iceland)	33138 (34%/66%)	62.7 (16.5)/ 57.1 (18.3)	98%	NA	Illumina Human Hap and Omni chips	NA	NA	NA	deCODEs own software	None	Data has relatives but relatedness was not taken into account	SNPTest
DGI	T2D case control	European (Sweden and Finland)	3005 (50%/50%)	61.0 (10.6)/ 62.5 (10.7)	95%	Sequenom fingerprint profile, gender check and relatedness	Affymetrix GeneChip® 500K	1x10^{-6}	95%	1%	MPUTE2	Birth place	Data has relatives but relatedness was not taken into account	SNPTest
EGCUT_370	Population based	European (Estonia)	2313 (48%/52%)	38.8 (16.3)/ 40.5 (15.9)	95%	Gender check, relatedness and MDS outlier	Illumina HumanHap 300	1x10^{-6}	95%	1%	IMPUTE2	None	Data has relatives but relatedness was not taken into account	SNPTest
EGCUT_omniX	Population based	European (Estonia)	4358 (57%/43%)	50.9 (19.7)/ 48.7 (19.3)	95%	Gender check, relatedness and MDS outlier	Illumina Human OmniExpress	1x10^{-6}	95%	1%	IMPUTE2	None	Data has relatives but relatedness was not taken into account	SNPTest
FINRISK	Other	European (Finland)	1560 (58%/42%)	55.5 (11.5)/ 60.3 (9.7)	95%	Heterozygosity, gender check and relatedness	Illumina Human610-Quad	1x10^{-6}	95% (99% if MAF<5%)	1%	IMPUTE2	PCA	No related individuals	SNPTest
FTC	Population based	European (Finland)	532 (51%/49%)	23.0 (1.5)/ 22.7 (1.2)	95%	Heterozygosity, gender check and relatedness	Illumina Human670-QuadCustom	1x10^{-6}	95% (99% if MAF<5%)	1 %	IMPUTE2	PCA	No related individuals	SNPTest
GenMets	Other case control	European (Finland)	1670 (49% 51%)	49.2 (10.5)/ 42.4 (11.6) , 51.8 (11.5)	95%	Heterozygosity, gender check and relatedness	Illumina Human610-Quad	1x10^{-6}	95% (99% if MAF<5%)	1%	IMPUTE2	PCA	No related individuals	SNPTest
GerMIFSI	CAD case control	European (Germany)	553 (72%/28%)	48.5 (7.5)/ 52.4 (8.8)	97%	NA	Affymetrix GeneChip® 500K	1x10^{-4} (in controls)	98%	1%	IMPUTE2	None	No related individuals	SNPTest
GerMIFSII	CAD case control	European (Germany)	388 (72%/28%)	51.4 (8.5)/ 53.8 (8.6)	98%	NA	Affymetrix Human SNP Array 6.0	1x10^{-4} (in controls)	98%	1%	IMPUTE2	None	No related individuals	SNPTest
GRAPHIC	Population based	European (England)	1017 (50%/50%)	53.8 (4.3)/ 51.9 (4.4)	95%	Gender check	Illumina Human OmniExpress	1x10^{-6}	95% (99% if MAF<5%)	1%	IMPUTE2	None	No related individuals	SNPTest
HBCS	Population based	European (Finland)	1619 (43%/57%)	61.4 (2.8)/ 61.6 (3.0)	95%	Heterozygosity, gender check and relatedness	Illumina Human670-QuadCustom	1x10^{-6}	95% (99% if MAF<5%)	1%	IMPUTE2	PCA	No related individuals	SNPTest
KORA F4	population based	European (Germany)	1996 (49%/51%)	55.8 (9.9)/ 55.0 (9.8)	93%	Gender check	Affymetrix Human SNP Array 6.0	5.7x10^{-7}	95% (99% if MAF<5%)	1%	IMPUTE2	None	No related individuals	SNPTest, ProbABEL
LLS	Other	European (The Netherlands)	2005 (45%/55%)	60.0 (6.7)/ 58.6 (6.6)	95%	Gender check and relatedness	Illumina Human660W-Quad and Illumina OmniExpress	1x10^{-4}	95%	1%	IMPUTE 2	None	Relatedness modelled	QT Assoc
NFBC1966	Population based	European (Finland)	5276 (48%/52%)	31.0 (0.0)/ 31.0 (0.0)	95%	Duplicates, consent withdrawal, heterozygosity, gender check and relatedness, MDS outlier	Illumina HumanCNV-370DUO	5.7x10^{-7}	95% (99% if MAF<5%)	1%	IMPUTE2	PCA	No related individuals	SNPTest
NTR/NESDA	Population based	European (The Netherlands)	7178 (37%/63%)	43.9 (15.3)/ 41.8 (14.0)	90%	Heterozygosity, gender check and relatedness	Illumina 370K, Quad 6.0, Omni 1M, Affymetrix 6.0 and Perlegen-Affymetrix 5.0	1x10^{-5}	95%	1%	IMPUTE2	None	Relatedness modelled	PLINK
PIVUS	Population based	European (Sweden)	949 (50%/50%)	70.1 (0.17)/ 70.3 (0.17)	95%	Heterozygosity, gender check and relatedness	Merged Metabochip and Illumina OmniExpress	1x10^{-6}	95% (99% if MAF<5%)	1%	IMPUTE2	PCA	No related individuals	SNPTest
RS1	Population based	European (The Netherlands)	5745 (41%/59%)	68.1 (8.2)/ 70.3 (9.6)	98%	Heterozygosity, gender check, relatedness and ethnic outliers	Illumina 550 and 610K	1x10^{-6}	98%	1%	Minimac	None	No related individuals	ProbABEL
Twingene	Population based	European (Sweden)	5898 (48%/52%)	65.7 (8.2)/ 64.6 (8.4)	97%	Heterozygosity, gender check and relatedness	Illumina Human OmniExpress	1x10^{-7}	97%	1%	IMPUTE2	PCA	No related individuals	SNPTest
ULSAM	Population based	European (Sweden)	1116 (100% /0%)	71.0 (0.63)/	95%	Heterozygosity, gender check and relatedness	Merged Metabochip and Illumina Omni 2.5M	1x10^{-6}	95% (99% if MAF<5%)	1%	IMPUTE2	PCA	No related individuals	SNPTest
YFS	Population based	European (Finland)	1989 (46%/54%)	37.6 (5.1)/ 37.6 (5.1)	95%	Heterozygosity, gender check and relatedness	Illumina Human670-QuadCustom	1x10^{-6}	95% (99% if MAF<5%)	1%	IMPUTE2	PCA	No related individuals	SNPTest
