# Supplementary material for: Discovery and Fine-Mapping of Glycaemic and Obesity-Related Trait Loci Using High-Density Imputation
Source: PLoS Genet. 2015 Jul 1;11(7):e1005230. doi: 10.1371/journal.pgen.1005230 (PMC4488845; doi:10.1371/journal.pgen.1005230)
Supplement: S2 Table — (PDF) [file pgen.1005230.s012.pdf]

S2 Table. Summary of obesity-related traits in each study.

Study	BMI				WHR_{adjBMI}			
	Sample size (males/females)	mean (SD) (males/females)	Adjustments	Genomic control lambda (males/females)	Sample size (males/females)	mean (SD) (males/females)	Adjustments	Genomic control lambda (males/females)
58BC-WTCCC	1453/1349	27.8 (4.3) / 26.8 (5.4)	none	1.00/0.99	1449/1344	0.93 (0.058) / 0.81 (0.061)	BMI	0.98/1.00
58BC-DIL	1247/1326	28.0 (4.2) / 27.0 (5.6)	none	0.99/0.99	1243/1318	0.93 (0.061) / 0.81 (0.063)	BMI	0.99/1.00
deCODE	11187/21951	27.8 (4.8) / 26.8 (5.5)	age + age²	1.19/1.32	3125/4009	0.96 (0.090) / 0.89 (0.009)	age + age² + BMI	1.05/1.09
DGI cases	777/753	28.2 (3.9) / 28.8 (4.9)	age + age² + center	1.00/1.01	581/557	0.99 (0.063) / 0.89 (0.072)	age + age² + BMI + center	1.00/1.01
DGI controls	713/762	26.6 (3.2) / 26.7 (4.2)	age + age² + center	1.00/1.01	385/435	0.96 (0.063) / 0.84 (0.066)	age + age² + BMI + center	1.00/1.01
EGCUT_370	1115/1198	25.9 (4.4) / 25.5 (5.6)	age + age²	0.98/1.00	608/699	0.88 (0.080) / 0.79 (0.075)	age + age² + BMI	1.00/1.00
EGCUT_omniX	2469/1889	26.8 (4.8) / 26.6 (5.7)	age + age²	0.99/0.99	2095/1569	0.93 (0.120) / 0.82 (0.082)	age + age² + BMI	1.03/1.00
FINRISK	902/658	27.3 (4.0) / 27.0 (5.0)	age + age²	0.99/1.01	912/659	0.96 (0.070) / 0.84 (0.007)	age + age² + BMI	0.99/1.00
FTC	259/273	23.8 (3.4) / 22.6 (3.8)	age + age²	1.02/1.00	NA	NA	NA	NA
GenMets Cases	415/410	29.5 (3.6) / 29.8 (4.9)	age + age² + 3 PCs	0.99/1.00	415/410	1.0 (0.0043) / 0.89 (0.052)	age + age² + BMI + 3 PCs	1.01/100
GenMets Controls	408/437	25.3 (3.1) / 25.1 (4.1)	age + age² + 3 PCs	1.02/1.01	407/436	0.95 (0.054) / 0.84 (0.055)	age + age² + BMI + 3 PCs	1.01/1.01
GerMIFSI	399/154	27.4 (3.4) / 27.1 (4.2)	age + age²	1.13/1.12	NA	NA	NA	NA
GerMIFSII	286/102	27.4 (3.3) / 28.5 (4.3)	age + age²	1.09/1.06	NA	NA	NA	NA
GRAPHIC	508/509	27.8 (3.9) / 27.1 (4.6)	age + age²	1.01/0.98	503/501	0.93 (0.070) / 0.81 (0.060)	age + age² + BMI	1.00/0.99
HBCS	702/917	27.6 (4.3) / 27.8 (5.1)	age + age²	1.01/0.99	701/915	0.99 (0.065) / 0.87 (0.078)	age + age² + BMI	1.00/1.00
KORA F4	979/1017	28.0 (3.9) / 27.5 (5.0)	age + age²	0.99/0.98	979/1017	0.95 (0.055) / 0.82 (0.063)	age + age² + BMI	1.02/1.00
LLS	904/1101	25.8 (3.0) / 25.1 (3.9)	age + age²	1.01/1.03	327/328	0.97 (0.065) / 0.91 (0.066)	age + age² + BMI	1.00/1.03
NFBC1966	2523/2753	25.2 (3.6) / 24.2 (4.7)	3 PCs	1.00/1.01	2243/2297	0.91 (0.058) / 0.81 (0.078)	BMI + 3 PCs	1.01/0.99
NTR/NESDA	2665/4513	25.5 (3.8) / 25.0 (4.7)	age + age² + platform + study	1.01/1.01	2665/4513	0.89 (0.081) / 0.80 (0.079)	age + age² + BMI + platform + study	1.04/1.02
PIVUS	474/475	27.0 (3.7) / 27.1 (4.9)	age + age² + PC1 + PC2	1.00/1.00	469/469	0.94 (0.064) / 0.86 (0.061)	age + age² + BMI + 2 PCs	0.99/1.01
RS1	2372/3373	25.7 (3.0) / 26.7 (4.1)	age + age²	1.02/1.02	2266/3202	0.96 (0.071) / 0.87 (0.088)	age + age² + BMI	1.00/1.02
Twingene	2780/3118	25.6 (3.3) / 24.7 (4.0)	age + age²	0.99/1.00	2763/3102	0.94 (0.15) / 0.83 (0.078)	age + age² + BMI	0.99/1.00
ULSAM	1116/-	26.3 (3.4) / NA	age + age² + 2 PCs	1.01/NA	1097/NA	0.95 (0.053) / NA	age + age² + BMI + 2 PCs	1.00/NA
YFS	908/1081	26.8 (4.3) / 25.3 (5.0)	age + age² + 3 PCs	1.01/1.00	913/1086	0.94 (0.070) / 0.83 (0.071)	age + age² + BMI + 3 PCs	1.00/1.00

PC, principal component.
