# Supplementary material for: Discovery and Fine-Mapping of Glycaemic and Obesity-Related Trait Loci Using High-Density Imputation
Source: PLoS Genet. 2015 Jul 1;11(7):e1005230. doi: 10.1371/journal.pgen.1005230 (PMC4488845; doi:10.1371/journal.pgen.1005230)
Supplement: S3 Table — (PDF) [file pgen.1005230.s013.pdf]

S3 Table. Summary of fasting glycaemic traits in each study.

Study	Samples with glucose phenotype					Samples with insulin phenotype					Sample exclusion criteria
	Measurements	Adjustments	*N* (M/F)	mean (SD), mmol/l (M/F)	Genomic control lambda (M/F)	Measurements	Adjustments	*N* (M/F)	mean (SD), pmol/l	Genomic control lambda (M/F)	
deCODE	Fasting plasma	age + age²	6902/10695	5.3 (0.55) / 5.1 (0.55)	1.26/1.16	Fasting serum	age + age² + BMI	682/1149	77.9 (68.6) / 70.8 (53.6)	1.17/1.21	Individuals with Diabetes (T2D, T1D), and/or FPG >=7 mmol/l, diabetic treatment
DGI controls	Fasting plasma	age + center	705/749	5.3 (0.56) / 5.3 (0.53)	1.02/1.00	Fasting serum	age + center + BMI	663/712	38.3 (41.2) / 33.3 (22.7)	1.00/1.00	Individuals with Diabetes
GenMets Cases	Fasting plasma	age + age² + 3 PCs	408/403	5.5 (0.50) / 5.5 (0.50)	1.00/1.00	Fasting plasma	age + age² + BMI + 3 PCs	400/396	85.8 (62.8) / 75.2 (41.8)	1.00/1.00	FG >= 7 mmol/l
GenMets Controls	Fasting plasma	age + age² + 3 PCs	404/435	5.3 (0.46) / 5.1 (0.40)	0.98/0.99	Fasting plasma	age + age² + BMI + 3 PCs	386/426	44.5 (23.0) / 42.7 (23.6)	1.00/1.00	FG >= 7 mmol/l
HBCS	Fasting plasma	age + age²	491/732	5.7 (0.54) / 5.4 (0.57)	1.00/0.99	Fasting plasma	age + age² + BMI	703/917	82.0 (62.3) / 70.6 (8.43)	1.00/1.00	FG >= 7 mmol/l
KORA F4	Fasting blood	age + age²	744/863	6.1 (0.47) / 5.8 (0.51)	1.00/1.00	Fasting blood	age + BMI	867/925	65.0 (135.7) / 58.1 (204.9)	0.89/0.88	Individuals with Diabetes (T2D, T1D), diabetes treatment, and/or FPG >=7 mmol/l, non-fasting, pregnant
NFBC1966	Fasting blood	3 PCs	2273/2413	5.8 (0.45) / 5.5 (0.45)	1.00/0.99	Fasting Serum	BMI + 3PCs	2255/2398	48.4 (14.9) / 44.0 (14.8)	0.99/0.99	Individuals with Diabetes (T2D, T1D), and/or FPG >=7 mmol/l, diabetic treatment
NTR/NESDA	Fasting plasma	age + age² + platform + study	2355/4175	5.4 (0.54) / 5.2 (0.55)	0.99/1.04	Fasting plasma	age + age² + platform	1836/3023	65.9 (42.4) / 62.3 (37.8)	1.05/1.01	Diabetes, FG> 7mmol/l
PIVUS	Fasting plasma	age + age² + 2 PCs	409/429	5.0 (0.50) / 4.9 (0.49)	0.99/1.01	Fasting serum	age + age² + BMI + 2PCs	409/429	59.6 (34.3) / 56.7 (33.8)	0.99/1.01	Individuals with Diabetes (T2D, T1D), and/or FPG >=7 mmol/l, non-fasting, diabetic treatment, pregnant
RS1	Fasting serum	age	1251/1713	5.6 (0.52) / 5.5 (0.56)	0.99/1.05	Fasting serum	age + BMI	1216/1697	72.6 (48.5) / 80.9 (61.2)	1.05/1.02	Individuals with diabetes
Twingene	Fasting serum	age + age²	2337/2925	6.0 (0.52) / 5.8 (0.50)	0.96/0.97	NA	NA	NA	NA	NA	Individuals with Diabetes (T2D, T1D), FG > 7 mmol/l
ULSAM	Fasting plasma	age + age² + PC1 + PC2	938/NA	5.3 (0.52) / NA	0.99/NA	Fasting serum	age + age² + BMI + 2PCs	927/NA	83.3 (45.0) / NA	1.01/NA	Individuals with Diabetes (T2D, T1D), and/or FPG >=7 mmol/l, non-fasting
YFS	Fasting plasma	age + age² + 3 PCs	824/1059	6.1 (0.45) / 5.8 (0.47)	0.99/1.00	Fasting plasma	age + age² + BMI + 3 PCs	883/1062	65.5 (53.6) / 59.6 (46.2)	0.98/0.99	FG >= 7 mmol/l

M, males; F, females. PC, principal component. FPG, fasting plasma glucose. T2D, type 2 diabetes; T1D, type 1 diabetes.
