# Supplementary material for: Discovery and Fine-Mapping of Glycaemic and Obesity-Related Trait Loci Using High-Density Imputation
Source: PLoS Genet. 2015 Jul 1;11(7):e1005230. doi: 10.1371/journal.pgen.1005230 (PMC4488845; doi:10.1371/journal.pgen.1005230)
Supplement: S4 Table — (PDF) [file pgen.1005230.s014.pdf]

S4 Table. Comparison of the SNP coverage between 1000 Genomes imputation and HapMap imputation in 1958 British Birth Cohort from the Wellcome Trust Case Control Consortium (a) and 1966 Northern Finnish Birth Cohort (b).

a. 1958 British Birth Cohort from the Wellcome Trust Case Control Consortium (n=2,802)

Minor allele frequency (MAF)[a]	1000 Genomes imputed			HapMap imputed		
	No. of total SNPs	No. of well-imputed SNPs (proportion)[b]	Mean INFO score	No. of total SNPs	No. of well-imputed SNPs (proportion)[b]	Mean INFO score
0.001≤MAF<0.005	3,440,835	2,771,080 (0.81)	0.73	120,551	110,687 (0.03)	0.87
0.005≤MAF<0.01	1,163,531	1,100,625 (0.95)	0.79	176,151	54,156 (0.05)	0.90
0.01≤MAF<0.02	1,120,045	1,092,438 (0.98)	0.84	93,920	93,452 (0.08)	0.95
0.02≤MAF<0.03	655,242	644,194 (0.98)	0.88	83,032	82,921 (0.13)	0.97
0.03≤MAF<0.04	472,084	464,280 (0.98)	0.91	77,228	77,186 (0.16)	0.98
0.04≤MAF<0.05	380,952	375,469 (0.99)	0.92	75,728	75,691 (0.20)	0.98
MAF≥0.05	6,308,553	6,229,729 (0.99)	0.96	2,300,971	2,300,523 (0.36)	0.99
Total	37,421,795	13,880,317		3,557,113	2,878,356	

b. 1966 Northern Finish Birth Cohort (n=5,276)

Minor allele frequency (MAF)[a]	1000 Genomes imputed			HapMap imputed		
	No. of total SNPs	No. of well-imputed SNPs (proportion)[b]	Mean INFO score	No. of total SNPs	No. of well-imputed SNPs (proportion)[b]	Mean INFO score
0.001≤MAF<0.005	3,148,071	1,975,647 (0.63)	0.69	131,254	90,404 (0.03)	0.73
0.005≤MAF<0.01	1,103,729	966,056 (0.88)	0.77	65,946	60,145 (0.05)	0.82
0.01≤MAF<0.02	1,112,612	1,063,907 (0.96)	0.83	99,959	97,628 (0.09)	0.88
0.02≤MAF<0.03	666,994	654,475 (0.98)	0.87	86,380	85,812 (0.13)	0.91
0.03≤MAF<0.04	476,400	470,302 (0.99)	0.90	79,728	79,498 (0.17)	0.93
0.04≤MAF<0.05	375,166	371,714 (0.99)	0.91	75,948	75,796 (0.20)	0.95
MAF≥0.05	5,938,415	5,918,942 (1.00)	0.96	2,284,446	2,283,784 (0.38)	0.98
Total	36,649,906	12,366,741		3,424,576	2,815,081	

[a] MAF is based on MAF calculated in each study.
[b] Proportion is calculated as the N (SNPs with INFO score >= 0.4) divided by the N (SNPs present in the 1000 Genomes EUR samples) in each MAF bin.
INFO, imputation quality score derived by IMPUTE2.
