# Supplementary material for: Discovery and Fine-Mapping of Glycaemic and Obesity-Related Trait Loci Using High-Density Imputation
Source: PLoS Genet. 2015 Jul 1;11(7):e1005230. doi: 10.1371/journal.pgen.1005230 (PMC4488845; doi:10.1371/journal.pgen.1005230)
Supplement: S5 Table — (PDF) [file pgen.1005230.s015.pdf]

S5 Table. Overlap of study samples between GIANT, MAGIC and current ENGAGE consortium.

	BMI			WHR_{adjBMI}			FG			FI_{adjBMI}		
	GIANT[a] Speliotes et al. (ref 17)	GIANT[b] Locke et al. (ref 26)	ENGAGE[c]	GIANT[a] Heid et al. (ref 18)	GIANT[b] Shungin et al. (ref 27)	ENGAGE[c]	MAGIC[a] Dupuis et al. (ref 16)	MAGIC[b] Scott et al. (ref 19)	ENGAGE[c]	MAGIC[a] Dupuis et al. (ref 16)	MAGIC[b] Scott et al. (ref 19)	ENGAGE[c]
**Study**	**Sample size (overlap)**	**Sample size (overlap)**	**Sample size**	**Sample size (overlap)**	**Sample size (overlap)**	**Sample size**	**Sample size (overlap)**	**Sample size (overlap)**	**Sample size**	**Sample size (overlap)**	**Sample size (overlap)**	**Sample size**
58BC-WTCCC	1,479 (1,479)	1,479 (1,479)	2,802	1,422 (1,422)	1,422 (1,422)	2,793						
58BC-DIL	2,587 (2,573)	2,587 (2,587)	2,573	2,490 (2,490)	2,592 (2,561)	2,561						
deCODE	26,799 (26,799)	26,799 (26,799)	33,138	5,883 (5,883)	5,513 (5,513)	7,134	6,059 (6,059)	6,059 (6,059)	17,597	1574 (1574)	1574 (1574)	1831
DGI cases	1,317 (1,317)	1,317 (1,317)	1,530	958 (958)	958 (958)	1,138						
DGI controls	1,088 (1,088)	1,088 (1,088)	1,475	469 (469)	469 (469)	820	1,467 (1,454)	1,467 (1,454)	1,454	1388 (1375)	1388 (1375)	1375
EGCUT_370		866 (866)	2,313		866 (866)	1,307		671 (0)				
EGCUT_omniX		1,356 (1,356)	4,358		1,164 (1,164)	3,664						
FINRISK			1,560			1,571						
FTC	138 (138)	125 (125)	532	138 (0)	138 (0)							
GenMets Cases	1,092 (825)	857 (825)	825	857 (825)	857 (825)	825			811			796
GenMets Controls	1,171 (845)	824 (824)	845	869 (843)	869 (843)	843			839			812
GerMIFSI	600 (553)	600 (553)	553									
GerMIFSII	1,124 (388)	1,124 (388)	388									
GRAPHIC			1,017			1,004						
HBCS		1,726 (1,619)	1,619		1,723 (1,616)	1,616			1,223			1620
KORA F4	1,814 (1,814)	1,811 (1,811)	1,996	1,814 (1,814)	1,814 (1,814)	1,996	458 (458)	2,634 (1,607)	1,607	435 (435)	871 (871)	1792
LLS		1,903 (1,903)	2,005		630 (630)	655						
NFBC1966	4,773 (4,773)	4,497 (4,497)	5,276	4,773 (4,540)	4,773 (4,540)	4,540	4,255 (4,255)	4,255 (4,255)	4,686	4234 (4234)	4234 (4234)	4653
NTR/NESDA	3,516 (3,516)	3,516 (3,516)	7,178	3,509 (3,509)	3,516 (3,516)	7,178	3,122 (3,122)	3,122 (3,122)	6,530	1268 (1268)	1268 (1268)	4859
PIVUS		978 (949)	949		968 (938)	938		929 (838)	838		923 (838)	838
RS1	5,744 (5,744)	5,744 (5,744)	5,745	5,468 (5,468)	5,373 (5,373)	5,468	2,058 (2,058)	2,058 (2,058)	2,964	2781 (2781)	2781 (2781)	2913
Twingene		9,176 (5,898)	5,898		9,123 (5,865)	5,865			5,262			
ULSAM		1,112 (1,116)	1,116		1,094 (1,094)	1,097		1,098 (938)	938		1085 (927)	927
YFS		1,989 (1,989)	1,989		1,999 (1,999)	1,999			1,883			1895
**Total**	**53,242 (51,852)**	**71,474 (67,249)**	**87,680**	**28,650 (28,221)**	**45,861 (42,006)**	**55,012**	**17,419 (17,406)**	**22,293 (20,331)**	**46,632**	**11,680 (11,667)**	**14,124 (14,026)**	**24,311**

[a] Combines GWAS data imputed up to HapMap reference panel.

[b] Combines previous GWAS data (HapMap imputed), new GWAS data (HapMap imputed) and Metabochip data.

[c] Combines GWAS data imputed up to 1000 Genomes reference panel.
