# Supplementary material for: Discovery and Fine-Mapping of Glycaemic and Obesity-Related Trait Loci Using High-Density Imputation
Source: PLoS Genet. 2015 Jul 1;11(7):e1005230. doi: 10.1371/journal.pgen.1005230 (PMC4488845; doi:10.1371/journal.pgen.1005230)
Supplement: S6 Table — (PDF) [file pgen.1005230.s016.pdf]

S6 Table. Established loci for glycaemic and obesity-related traits achieving genome-wide significance ($p<5x10^{-8}$).

Trait	Locus	Lead SNP	Chr	Position (b37)	Alleles		Male meta-analysis				Female-meta-analysis				Sex-combined meta-analysis			
					Effect/ Other	EAF	Effect (SE)	*p*-value	*Q p*-value	*N*	Effect (SE)	*p*-value	*Q p*-value	*N*	Effect (SE)	*p*-value	Sex heterogeneity *p*-value	*N*
BMI	*NEGR1*	rs11209943	1	72,750,500	G/A	0.59	0.027 (0.008)	3.9E-04	7.8E-01	37,287	0.030 (0.007)	1.7E-05	1.2E-01	49,709	0.029 (0.005)	2.2E-08	7.8E-01	86,996
BMI	*SEC16B*	rs539515	1	177,889,025	C/A	0.20	0.030 (0.009)	1.3E-03	8.9E-01	37,283	0.052 (0.009)	2.5E-09	1.8E-01	49,679	0.042 (0.006)	7.0E-11	1.0E-01	86,962
BMI	*TMEM18*	rs66553418	2	630,902	T/A	0.82	0.058 (0.010)	5.8E-09	8.2E-01	37,040	0.064 (0.009)	2.0E-12	2.5E-01	49,340	0.062 (0.007)	6.0E-20	6.7E-01	86,380
BMI	*RBJ-ADCY3-POMC*	rs6749422	2	25,150,011	G/C	0.45	0.022 (0.007)	3.0E-03	3.2E-01	37,235	0.036 (0.007)	1.4E-07	1.9E-01	49,610	0.030 (0.005)	4.0E-09	1.6E-01	86,845
BMI	*ETV5*	rs9816226	3	185,834,499	A/T	0.81	0.046 (0.010)	3.0E-06	9.3E-01	37,249	0.036 (0.009)	5.2E-05	2.0E-02	49,614	0.040 (0.007)	1.1E-09	4.7E-01	86,863
BMI	*GNPDA2*	rs12507026	4	45,181,334	T/A	0.42	0.044 (0.008)	4.8E-08	5.5E-01	34,736	0.038 (0.007)	2.4E-07	8.0E-02	46,894	0.041 (0.005)	6.2E-14	5.5E-01	81,630
BMI	*GALNT10*	rs11958496	5	153,546,602	G/A	0.42	0.043 (0.009)	6.2E-07	5.2E-01	31382	0.040 (0.008)	1.5E-04	8.7E-01	41,989	0.034 (0.005)	3.9E-10	3.4E-01	79,787
BMI	*TFAP2B*	rs3798519	6	50,788,778	C/A	0.20	0.051 (0.009)	5.0E-08	4.0E-01	37,269	0.040 (0.009)	5.1E-06	4.0E-02	49,681	0.045 (0.006)	2.0E-12	3.8E-01	86,950
BMI	*DTX2P1-UPK3BP1-PMS2P11*	rs7804790	7	76,568,075	C/T	0.20	0.050 (0.013)	8.1E-05	2.6E-01	30,964	0.044 (0.012)	2.6E-04	3.6E-01	41,956	0.045 (0.008)	$2.8x10^{-8}$	4.3E-01	79,335
BMI	*GRID1*	rs7903554	10	87,355,751	G/C	0.07	0.065 (0.017)	8.3E-05	1.7E-01	34,054	0.053 (0.015)	3.0E-04	6.7E-01	46,479	0.059 (0.011)	$1.7x10^{-8}$	4.5E-01	86,947
BMI	*BDNF*	rs4517468	11	27,688,286	A/T	0.34	0.034 (0.008)	1.6E-05	1.6E-01	37,262	0.037 (0.007)	4.6E-07	3.8E-01	49,660	0.036 (0.005)	2.2E-11	8.2E-01	86,922
BMI	*FAIM2*	rs7132908	12	50,263,148	A/G	0.38	0.040 (0.008)	2.2E-07	4.8E-01	37,262	0.035 (0.007)	7.4E-07	3.5E-01	49,643	0.037 (0.005)	9.6E-13	6.5E-01	86,905
BMI	*NRXN3*	rs7141420	14	79,899,454	T/C	0.52	0.035 (0.007)	3.3E-06	5.0E-02	37,271	0.034 (0.007)	5.2E-07	5.4E-01	49,674	0.035 (0.005)	8.3E-12	9.6E-01	86,945
BMI	*MAP2K5*	rs4776972	15	68,083,436	A/C	0.81	0.038 (0.010)	1.2E-04	8.2E-01	37,228	0.049 (0.009)	4.3E-08	9.4E-01	49,591	0.044 (0.007)	4.6E-11	4.2E-01	86,819
BMI	*GPRC5B*	rs7190603	16	19,928,662	T/C	0.87	0.053 (0.011)	2.3E-06	6.0E-02	37,204	0.056 (0.010)	5.4E-08	6.2E-01	49,457	0.055 (0.008)	4.7E-13	8.4E-01	86,661
BMI	*SH2B1*	rs2008514	16	28,825,605	A/G	0.43	0.041 (0.008)	2.7E-07	2.9E-01	34,720	0.033 (0.007)	5.7E-06	1.6E-01	46,822	0.036 (0.005)	1.4E-11	4.2E-01	81,542
BMI	*FTO*	rs55872725	16	53,809,123	T/C	0.42	0.088 (0.008)	7.8E-29	6.9E-01	34,727	0.072 (0.007)	3.4E-24	2.0E-01	46,824	0.079 (0.005)	1.0E-50	1.5E-01	81,551
BMI	*MC4R*	rs663129	18	57,838,401	A/G	0.24	0.059 (0.009)	2.8E-11	8.4E-01	37,258	0.054 (0.008)	2.6E-11	2.8E-03	49,632	0.057 (0.006)	3.2E-21	6.5E-01	86,890
WHR_{adjBMI}	*LY86*	rs1294437	6	6,749,789	C/T	0.66	0.031 (0.010)	1.2E-03	3.3E-01	25,918	0.041 (0.009)	5.7E-06	7.4E-01	28,595	0.036 (0.007)	3.0E-08	4.3E-01	54,513
WHR_{adjBMI}	*VEGFA*	rs6905288	6	43,758,873	A/G	0.56	0.031 (0.010)	1.3E-03	5.9E-01	25,927	0.055 (0.009)	1.5E-09	1.8E-01	28,612	0.043 (0.007)	4.9E-11	6.4E-02	54,539

WHR$_{adjBMI}$	*RSPO3*	rs72959041	6	127,454,893	A/G	0.08	0.077 (0.021)	2.1E-04	5.7E-01	23,266	0.140 (0.021)	1.8E-11	1.7E-01	24,107	0.108 (0.015)	1.7E-13	3.4E-02	47,373
FG	*PROX1*	rs340876	1	214,158,132	T/C	0.55	0.022 (0.007)	8.7E-04	3.2E-01	17,710	0.031 (0.006)	3.4E-07	5.5E-01	23,645	0.028 (0.004)	1.3E-11	3.3E-01	46,617
FG	*GCKR*	rs1260326	2	27,730,940	C/T	0.64	0.032 (0.007)	1.9E-06	1.3E-01	17,704	0.037 (0.006)	1.6E-09	1.2E-01	23,617	0.033 (0.004)	2.2E-15	5.8E-01	46,583
FG	*G6PC2*	rs560887	2	169,763,148	C/T	0.69	0.084 (0.008)	1.1E-27	2.8E-06	15,363	0.097 (0.007)	3.4E-39	4.9E-05	19,466	0.087 (0.005)	1.5E-72	2.3E-01	40,091
FG	*SLC2A2*	rs7356034	3	170,732,599	G/A	0.73	0.031 (0.007)	2.4E-05	2.7E-01	17,725	0.025 (0.007)	1.3E-04	4.8E-01	23,674	0.025 (0.004)	1.5E-08	5.7E-01	46,661
FG	*PCSK1*	rs144489757	5	95,694,609	C/G	0.70	0.024 (0.007)	7.7E-04	5.6E-01	17,716	0.031 (0.006)	1.1E-06	7.2E-01	23,616	0.025 (0.004)	7.3E-09	4.3E-01	46,594
FG	*CDKAL1*	rs7747724	6	20,751,315	T/C	0.58	0.018 (0.007)	1.6E-02	7.8E-01	15,357	0.029 (0.007)	2.8E-05	1.9E-01	19,449	0.027 (0.005)	3.5E-09	2.4E-01	40,068
FG	*DGKB-TMEM195*	rs13220985	7	15,062,694	A/G	0.51	0.042 (0.007)	8.1E-11	4.5E-01	17,696	0.029 (0.006)	1.0E-06	8.2E-01	23,628	0.033 (0.004)	1.4E-16	1.2E-01	46,586
FG	*GCK*	rs878521	7	44,255,643	A/G	0.23	0.059 (0.008)	7.3E-14	9.9E-01	17,726	0.070 (0.007)	1.4E-22	2.0E-02	23,662	0.062 (0.005)	1.0E-36	2.9E-01	46,650
FG	*SLC30A8*	rs11558471	8	118,185,733	A/G	0.65	0.023 (0.008)	2.4E-03	3.4E-02	15,348	0.038 (0.007)	1.0E-07	5.5E-01	19,494	0.029 (0.005)	3.2E-10	1.5E-01	40,104
FG	*ADRA2A*	rs35964103	10	113,002,526	C/T	0.91	0.045 (0.012)	1.1E-04	4.8E-01	17,712	0.043 (0.010)	2.9E-05	1.2E-01	23,636	0.041 (0.007)	8.5E-09	9.0E-01	46,610
FG	*TCF7L2*	rs34872471	10	114,754,071	C/T	0.26	0.027 (0.008)	5.4E-04	8.5E-01	17,720	0.026 (0.007)	2.3E-04	5.0E-02	23,641	0.027 (0.005)	2.1E-08	9.2E-01	46,623
FG	*MTNR1B*	rs10830963	11	92,708,710	G/C	0.27	0.092 (0.008)	5.9E-29	1.3E-04	15,355	0.082 (0.008)	5.7E-25	1.0E-03	19,461	0.084 (0.005)	1.0E-61	4.0E-01	40,077
FG	*VPS13C-C2CD4A/B-FAM148B*	rs1881415	15	62,388,530	T/C	0.52	0.025 (0.007)	1.5E-04	4.7E-01	17,705	0.019 (0.006)	1.4E-03	5.3E-01	23,623	0.023 (0.004)	1.7E-08	5.0E-01	46,590
FI$_{adjBMI}$	*GCKR*	rs1260326	2	27,730,940	C/T	0.64	0.026 (0.007)	7.4E-05	3.0E-01	11,164	0.034 (0.006)	6.4E-08	2.8E-03	13,075	0.030 (0.005)	5.8E-11	4.0E-01	24,239
