# Supplementary material for: Discovery and Fine-Mapping of Glycaemic and Obesity-Related Trait Loci Using High-Density Imputation
Source: PLoS Genet. 2015 Jul 1;11(7):e1005230. doi: 10.1371/journal.pgen.1005230 (PMC4488845; doi:10.1371/journal.pgen.1005230)
Supplement: S7 Table — (PDF) [file pgen.1005230.s017.pdf]

S7 Table. Summary of lead SNPs achieving genome-wide significance in established loci for BMI, WHR_{adjBMI}, FG and FI_{adjBMI}.

Locus	Chr	Position (b37)	Index SNP	Trait	Alleles (Effect/ Other)	Effect allele freq.	Meta-analysis Effect (SE)	Meta-analysis *p*-value	Meta-analysis *N*	New lead SNP?	Present in HapMap?	Rationale for inclusion in this table	r^2 with previously reported lead SNP	New lead SNP *p*-value conditional on previous lead SNP
	1	72,750,500	rs11209943	BMI	G/A	0.59	0.029 (0.005)	2.2E-08	86,996	Y	N	Top SNP from global meta-analysis	1	NA
NEGR1	1	72,812,440	rs2815752	BMI	A/G	0.60	0.028 (0.005)	5.8E-08	87,002			Previously reported lead SNP from Speliotes et al (Nature Genetics 2010)	-	
	1	177,889,025	rs539515	BMI	C/A	0.20	0.042 (0.006)	7.0E-11	86,962	Y	N	Top SNP from global meta-analysis	1	NA
SEC16B	1	177,889,480	rs543874	BMI	G/A	0.20	0.041 (0.006)	1.1E-10	86,964			Previously reported lead SNP from Speliotes et al (Nature Genetics 2010)	-	
	2	630,902	rs66553418	BMI	T/A	0.82	0.062 (0.007)	6.0E-20	86,380	Y	N	Top SNP from global meta-analysis	0.94	0.91
TMEM18	2	622,827	rs2867125	BMI	C/T	0.83	0.060 (0.007)	2.2E-17	79,216			Previously reported lead SNP from Speliotes et al (Nature Genetics 2010)	-	
	2	25,150,011	rs6749422	BMI	G/C	0.45	0.030 (0.005)	4.0E-09	86,845	Y	N	Top SNP from global meta-analysis	0.96	0.079
RBJ-ADCY3-POMC	2	25,158,008	rs713586	BMI	C/T	0.46	0.029 (0.005)	1.7E-08	86,856			Previously reported lead SNP from Speliotes et al (Nature Genetics 2010)	-	
ETV5	3	185,834,499	rs9816226	BMI	A/T	0.81	0.040 (0.007)	1.1E-09	86,863	N	Y	Top SNP from global meta-analysis	1	NA
	4	45,181,334	rs12507026	BMI	T/A	0.42	0.041 (0.005)	6.2E-14	81,630	Y	N	Top SNP from global meta-analysis	1	NA
GNPDA2	4	45,182,527	rs10938397	BMI	G/A	0.42	0.041 (0.005)	6.8E-14	81,537			Previously reported lead SNP from Speliotes et al (Nature Genetics 2010)	-	
	5	153,546,602	rs11958496	BMI	G/A	0.42	0.034 (0.005)	3.9E-10	79,787	Y	N	Top SNP from global meta-analysis	0.75	0.045
GALNT10	5	153,543,466	rs7708584	BMI	A/G	0.41	0.032 (0.005)	2.9E-09	79,791			Previously reported lead SNP from Monda et al (Nature Genetics 2013)	-	
	6	50,788,778	rs3798519	BMI	C/A	0.20	0.045 (0.006)	2.0E-12	86,950	Y	N	Top SNP from global meta-analysis	0.90	0.022
TFAP2B	6	50,803,050	rs987237	BMI	G/A	0.20	0.045 (0.007)	2.9E-11	79,777			Previously reported lead SNP from Speliotes et al (Nature Genetics 2010)	-	
DTX2P1-UPK3BP1-	7	76,568,075	rs7804790	BMI	C/T	0.20	0.045 (0.008)	2.8E-08	79,335	Y	N	Top SNP from global meta-analysis	1	0.92
PMS2P11	7	76,608,143	rs2245368	BMI	C/T	0.23	0.037 (0.008)	2.1E-06	73,564			Previously reported lead SNP from Locke et al (Nature 2014)	-	
	10	87,355,751	rs7903554	BMI	G/C	0.07	0.059 (0.011)	1.7E-08	86,947	Y	N	Top SNP from global meta-analysis	0.86	0.94
GRID1	10	87,410,904	rs7899106	BMI	G/A	0.07	0.054 (0.011)	8.4E-07	86,944			Previously reported lead SNP from Locke et al (Nature 2014)	-	
	11	27,688,286	rs4517468	BMI	A/T	0.34	0.036 (0.005)	2.2E-11	86,922	Y	N	Top SNP from global meta-analysis	0.10	1.9E-06
BDNF	11	27,725,986	rs10767664	BMI	A/T	0.81	0.041 (0.006)	2.6E-10	86,916			Previously reported lead SNP from Speliotes et al (Nature Genetics 2010)	-	
	12	50,263,148	rs7132908	BMI	A/G	0.38	0.037 (0.005)	9.6E-13	86,905	Y	Y	Top SNP from global meta-analysis	0.89	0.60
FAIM2	12	50,247,468	rs7138803	BMI	A/G	0.38	0.034 (0.005)	3.6E-10	86,923			Previously reported lead SNP from Speliotes et al (Nature Genetics 2010)	-	
	14	79,899,454	rs7141420	BMI	T/C	0.52	0.035 (0.005)	8.3E-12	86,945	Y	Y	Top SNP from global meta-analysis	0.29	2.2E-05
NRXN3	14	79,936,964	rs10150332	BMI	C/T	0.22	0.039 (0.006)	2.6E-10	86,941			Previously reported lead SNP from Speliotes et al (Nature Genetics 2010)	-	
	15	68,083,436	rs4776972	BMI	A/C	0.81	0.044 (0.007)	4.6E-11	86,819	Y	N	Top SNP from global meta-analysis	0.84	0.035
MAP2K5	15	68,086,838	rs2241423	BMI	G/A	0.79	0.042 (0.007)	2.9E-10	79,649			Previously reported lead SNP from Speliotes et al (Nature Genetics 2010)	-	
	16	19,928,662	rs7190603	BMI	T/C	0.87	0.055 (0.008)	4.7E-13	86,661	Y	N	Top SNP from global meta-analysis	1	NA
GPRC5B	16	19,933,600	rs12444979	BMI	C/T	0.87	0.055 (0.008)	7.5E-13	86,659			Previously reported lead SNP from Speliotes et al (Nature Genetics 2010)	-	
	16	28,825,605	rs2008514	BMI	A/G	0.43	0.036 (0.005)	1.4E-11	81,542	Y	Y	Top SNP from global meta-analysis	0.97	0.038
SH2B1	16	28,885,659	rs7359397	BMI	T/C	0.42	0.036 (0.006)	1.2E-10	74,356			Previously reported lead SNP from Speliotes et al (Nature Genetics 2010)	-	
	16	53,809,123	rs55872725	BMI	T/C	0.42	0.079 (0.005)	1.0E-50	81,551	Y	N	Top SNP from global meta-analysis	1	NA
FTO	16	53,803,574	rs1558902	BMI	A/T	0.42	0.079 (0.005)	1.3E-50	81,550			Previously reported lead SNP from Speliotes et al (Nature Genetics 2010)	-	
	18	57,838,401	rs663129	BMI	A/G	0.24	0.057 (0.006)	3.2E-21	86,890	Y	Y	Top SNP from global meta-analysis	1	NA
MC4R	18	57,839,769	rs571312	BMI	A/C	0.24	0.054 (0.006)	6.3E-18	79,714			Previously reported lead SNP from Speliotes et al (Nature Genetics 2010)	-	
	6	6,749,789	rs1294437	WHR_{adjBMI}	C/T	0.66	0.036 (0.007)	3.0E-08	54,513	Y	N	Top SNP from global meta-analysis	0.75	0.095
LY86	6	6,743,149	rs1294421	WHR_{adjBMI}	G/T	0.62	0.034 (0.006)	9.4E-08	54,524			Previously reported lead SNP from Heid et al (Nature Genetics 2010)	-	
VEGFA	6	43,758,873	rs6905288	WHR_{adjBMI}	A/G	0.56	0.043 (0.007)	4.9E-11	54,539	N	Y	Top SNP from global meta-analysis	1	NA
	6	127,454,893	rs72959041	WHR_{adjBMI}	A/G	0.08	0.108 (0.015)	1.7E-13	47,373	Y	N	Top SNP from global meta-analysis	0.04	8.4E-11
RSPO3	6	127,452,639	rs9491696	WHR_{adjBMI}	G/C	0.49	0.041 (0.006)	2.1E-11	54,555			Previously reported lead SNP from Heid et al (Nature Genetics 2010)	-	
	1	214,158,132	rs340876	FG	T/C	0.55	0.028 (0.004)	1.3E-11	46,617	Y	N	Top SNP from global meta-analysis	0.82	0.020
PROX1	1	214,159,256	rs340874	FG	C/T	0.53	0.029 (0.004)	8.8E-11	40,091			Previously reported lead SNP from Dupuis et al. (Nature Genetics, 2010)	-	
	2	27,730,940	rs1260326	FG	C/T	0.64	0.033 (0.004)	2.2E-15	46,583	Y	Y	Top SNP from global meta-analysis	0.93	0.0068
GCKR	2	27,741,237	rs780094	FG	C/T	0.65	0.031 (0.004)	8.5E-14	46,588			Previously reported lead SNP from Dupuis et al. (Nature Genetics, 2010)	-	
G6PC2	2	169,763,148	rs560887	FG	C/T	0.69	0.087 (0.005)	1.5E-72	40,091	N	Y	Top SNP from global meta-analysis	1	NA
	3	170,732,599	rs7356034	FG	G/A	0.73	0.025 (0.004)	1.5E-08	46,661	Y	N	Top SNP from global meta-analysis	0.42	8.6E-05
SLC2A2	3	170,717,521	rs11920090	FG	T/A	0.88	0.028 (0.007)	1.8E-05	40,119			Previously reported lead SNP from Dupuis et al. (Nature Genetics, 2010)	-	
	5	95,694,609	rs144489757	FG	C/G	0.70	0.025 (0.004)	7.3E-09	46,594	Y	N	Top SNP from global meta-analysis	0.62	6.9E-04
PCSK1	5	95,539,448	rs4869272	FG	T/C	0.68	0.022 (0.005)	2.6E-06	40,106			Previously reported lead SNP from Scott et al. (Nat Gen 2012)	-	
	6	20,751,315	rs7747724	FG	T/C	0.58	0.027 (0.005)	3.5E-09	40,068	Y	Y	Top SNP from global meta-analysis	0.21	1.7E-06
CDKAL1	6	20,686,996	rs9368222	FG	A/C	0.27	0.017 (0.005)	3.8E-04	40,058			Previously reported lead SNP from Scott et al. (Nat Gen 2012)	-	
	7	15,062,694	rs13220985	FG	A/G	0.51	0.033 (0.004)	1.4E-16	46,586	Y	N	Top SNP from global meta-analysis	1	NA
DGKB-TMEM195	7	15,064,309	rs2191349	FG	T/G	0.50	0.034 (0.004)	7.9E-15	40,053			Previously reported lead SNP from Dupuis et al. (Nature Genetics, 2010)	-	
	7	44,255,643	rs878521	FG	A/G	0.23	0.062 (0.005)	1.0E-36	46,650	Y	N	Top SNP from global meta-analysis	0.55	4.7E-14
GCK	7	44,235,668	rs4607517	FG	A/G	0.13	0.068 (0.007)	3.9E-25	40,112			Previously reported lead SNP from Dupuis et al. (Nature Genetics, 2010)	-	

SLC30A8	8	118,185,733	rs11558471	FG	A/G	0.65	0.029 (0.005)	3.2E-10	40,104	N	Y	Top SNP from global meta-analysis	1	NA
ADRA2A	10	113,002,526	rs35964103	FG	C/T	0.91	0.041 (0.007)	8.5E-09	46,610	Y	N	Top SNP from global meta-analysis	0.50	0.0013
	10	113,042,093	rs10885122	FG	G/T	0.88	0.032 (0.007)	1.3E-06	40,082			Previously reported lead SNP from Dupuis et al. (Nature Genetics, 2010)	-	
TCF7L2	10	114,754,071	rs34872471	FG	C/T	0.26	0.027 (0.005)	2.1E-08	46,623	Y	N	Top SNP from global meta-analysis	0.89	0.015
	10	114,756,041	rs4506565	FG	T/A	0.27	0.026 (0.005)	4.1E-07	40,098			Previously reported lead SNP from Dupuis et al. (Nature Genetics, 2010)	-	
MTNR1B	11	92,708,710	rs10830963	FG	G/C	0.27	0.084 (0.005)	1.0E-61	40,077	N	Y	Top SNP from global meta-analysis	1	NA
VPS13C-C2CD4AB-FAM148B	15	62,388,530	rs1881415	FG	T/C	0.52	0.023 (0.004)	1.7E-08	46,590	Y	N	Top SNP from global meta-analysis	0.20	1.8E-08
	15	62,433,962	rs11071657	FG	A/G	0.62	0.006 (0.005)	1.9E-01	40,060			Previously reported lead SNP from Dupuis et al. (Nature Genetics, 2010)	-	
GCKR	2	27,730,940	rs1260326	FI_{adjBMI}	C/T	0.64	0.030 (0.005)	5.8E-11	24,239	Y	Y	Top SNP from global meta-analysis	0.93	0.0068
	2	27,741,237	rs780094	FI_{adjBMI}	C/T	0.64	0.027 (0.005)	3.2E-09	24,241			Previously reported lead SNP from Dupuis et al. (Nature Genetics, 2010)	-	
