# Supplementary material for: Discovery and Fine-Mapping of Glycaemic and Obesity-Related Trait Loci Using High-Density Imputation
Source: PLoS Genet. 2015 Jul 1;11(7):e1005230. doi: 10.1371/journal.pgen.1005230 (PMC4488845; doi:10.1371/journal.pgen.1005230)
Supplement: S8 Table — (PDF) [file pgen.1005230.s018.pdf]

S8 Table. Approximate conditional analysis results for BMI, WHR_{adjBMI}, FG and FI_{adjBMI}.

a. BMI

Index SNP	Chr	Position	Locus	Effect allele	Meta-analysis			Joint analysis with LD estimated from 58BC-WTCCC		Joint analysis with LD estimated from NFBC1966	
					EAF	β (SE)	*P*	B (SE)	*P*	B (SE)	*P*
rs11209943	1	72,750,500	*NEGR1*	A	0.41	-0.029 (0.005)	2.2E-08	-0.029 (0.005)	2.1E-08	-0.029 (0.005)	2.1E-08
rs539515	1	177,889,025	*SEC16B*	A	0.80	-0.042 (0.006)	7.0E-11	-0.042 (0.006)	6.9E-11	-0.042 (0.006)	6.9E-11
rs5017305	2	630,902	*TMEM18*	A	0.18	-0.062 (0.007)	6.0E-20	-0.062 (0.007)	5.9E-20	-0.062 (0.007)	5.9E-20
rs6749422	2	25,150,011	*RBJ-ADCY3-POMC*	C	0.55	-0.030 (0.005)	4.0E-09	-0.030 (0.005)	3.9E-09	-0.030 (0.005)	3.9E-09
rs9816226	3	185,834,499	*ETV5*	A	0.19	-0.040 (0.007)	1.1E-09	-0.040 (0.007)	1.1E-09	-0.040 (0.007)	1.1E-09
rs12507026	4	45,181,334	*GNPDA2*	A	0.58	-0.041 (0.005)	6.2E-14	-0.041 (0.005)	6.0E-14	-0.041 (0.005)	6.0E-14
rs11958496	5	153,546,602	*GALNT10*	G	0.42	0.034 (0.005)	3.9E-10	0.034 (0.005)	3.8E-10	0.034 (0.005)	3.8E-10
rs3798519	6	50,788,778	*TFAP2B*	A	0.80	-0.045 (0.006)	2.0E-12	-0.045 (0.006)	1.9E-12	-0.045 (0.006)	1.9E-12
rs7804790	7	76,568,075	*DTX2P1-UPK3BP1-PMS2P11*	C	0.20	0.045 (0.008)	2.8E-08	0.045 (0.008)	2.7E-08	0.045 (0.008)	2.7E-08
rs7903554	10	87,355,751	*GRID1*	C	0.93	-0.059 (0.011)	1.7E-08	-0.059 (0.011)	1.6E-08	-0.059 (0.011)	1.6E-08
rs4517468	11	27,688,286	*BDNF*	A	0.34	0.036 (0.005)	2.2E-11	0.036 (0.005)	2.1E-11	0.036 (0.005)	2.1E-11
rs7132908	12	50,263,148	*FAIM2*	G	0.62	-0.037 (0.005)	9.6E-13	-0.037 (0.005)	9.4E-13	-0.037 (0.005)	9.4E-13
rs1966714	12	90,671,038	*EPYC*	A	0.46	0.036 (0.006)	1.9E-08	0.036 (0.006)	1.9E-08	0.036 (0.007)	1.9E-08
rs12885467	14	33,303,788	*AKAP6*	T	0.51	-0.029 (0.005)	4.5E-08	-0.029 (0.005)	4.4E-08	-0.030 (0.005)	1.7E-08
rs7141420	14	79,899,454	*NRXN3*	C	0.48	-0.035 (0.005)	8.3E-12	-0.035 (0.005)	8.1E-12	-0.035 (0.005)	8.1E-12
rs4776972	15	68,083,436	*MAP2K5*	A	0.81	0.044 (0.007)	4.6E-11	0.044 (0.007)	4.4E-11	0.044 (0.007)	4.4E-11
rs7204864	16	19,942,927	*GPRC5B*	C	0.87	0.055 (0.008)	4.7E-13	0.056 (0.008)	1.5E-13	0.056 (0.008)	2.8E-13
rs2008514	16	28,825,605	*SH2B1*	G	0.57	-0.036 (0.005)	1.4E-11	-0.037 (0.005)	4.4E-12	-0.037 (0.005)	8.1E-12
rs55872725	16	53,809,123	*FTO*	C	0.58	-0.079 (0.005)	1.0E-50	-0.079 (0.005)	1.2E-50	-0.079 (0.005)	1.2E-50
rs663129	18	57,838,401	*MC4R*	G	0.76	-0.057 (0.006)	3.2E-21	-0.057 (0.006)	3.1E-21	-0.057 (0.006)	3.1E-21

b. WHR_{adjBMI}

Index SNP	Chr	Position	Locus	Effect allele	Meta-analysis			Joint analysis with LD estimated from 58BC-WTCCC		Joint analysis with LD estimated from NFBC1966	
					EAF	β (SE)	*P*	B (SE)	*P*	B (SE)	*P*
rs1294437	6	6,749,789	*LY86*	C	0.66	0.036 (0.007)	3.0E-08	0.036 (0.007)	3.0E-08	0.036 (0.007)	3.0E-08
rs6905288	6	43,758,873	*VEGFA*	G	0.44	-0.043 (0.007)	4.9E-11	-0.043 (0.007)	4.8E-11	-0.043 (0.007)	4.8E-11
rs72959041	6	127,454,893	*RSPO3*	G	0.92	-0.110 (0.015)	1.7E-13	-0.096 (0.015)	9.8E-11	-0.092 (0.015)	6.6E-10
rs4509142	6	127,489,001	*RSPO3*	C	0.49	-0.043 (0.006)	2.9E-12	-0.038 (0.006)	1.7E-09	-0.036 (0.006)	1.2E-08

c. FG

Index SNP	Chr	Position	Locus	Effect allele	Meta-analysis			Joint analysis with LD estimated from 58BC-WTCCC		Joint analysis with LD estimated from NFBC1966	
					EAF	β (SE)	*P*	B (SE)	*P*	B (SE)	*P*
rs340876	1	214,158,132	*PROX1*	C	0.45	-0.028 (0.004)	1.3E-11	-0.028 (0.004)	1.3E-11	-0.028 (0.004)	1.3E-11
rs1260326	2	27,730,940	*GCKR*	T	0.36	-0.033 (0.004)	2.2E-15	-0.033 (0.004)	2.2E-15	-0.033 (0.004)	2.2E-15
rs150171632	2	169,748,691	*G6PC2*	C	0.99	0.182 (0.020)	3.0E-19	0.192 (0.020)	4.1E-21	0.259 (0.023)	2.0E-29
rs11679727	2	169,754,098	*G6PC2*	A	0.77	0.089 (0.006)	3.2E-54	0.041 (0.007)	9.1E-09	0.051 (0.007)	6.2E-15
2-169754143	2	169,754,143	*G6PC2*	C	0.92	0.100 (0.010)	5.2E-25			0.067 (0.010)	8.3E-12
rs34177044	2	169,754,485	*G6PC2*	G	0.78	-0.069 (0.005)	2.4E-43	-0.042 (0.005)	1.1E-15	-0.053 (0.005)	1.9E-24
rs16856159	2	169,759,903	*G6PC2*	G	0.91	-0.020 (0.007)	5.4E-03			-0.072 (0.008)	5.0E-18
rs560887	2	169,763,148	*G6PC2*	T	0.31	-0.087 (0.005)	1.5E-72	-0.055 (0.006)	8.3E-19		
rs1101532	2	169,810,416	*G6PC2*	T	0.46	-0.060 (0.004)	4.3E-51			-0.034 (0.005)	2.3E-13
rs7356034	3	170,732,599	*SLC2A2*	G	0.73	0.025 (0.004)	1.5E-08	0.025 (0.004)	1.5E-08	0.025 (0.005)	1.5E-08
rs144489757	5	95,694,609	*PCSK1*	C	0.70	0.025 (0.004)	7.3E-09	0.025 (0.004)	7.2E-09	0.025 (0.004)	7.2E-09
rs7747724	6	20,751,315	*CDKAL1*	T	0.58	0.027 (0.005)	3.5E-09	0.027 (0.005)	3.4E-09	0.027 (0.005)	3.4E-09
rs13220985	7	15,062,694	*DGKB-TMEM195*	A	0.51	0.033 (0.004)	1.4E-16	0.033 (0.004)	1.3E-16	0.033 (0.004)	1.3E-16
rs2268577	7	44,189,010	*GCK*	C	0.82	-0.045 (0.005)	8.6E-18			-0.039 (0.005)	2.3E-13
rs10259649	7	44,219,705	*GCK*	T	0.78	-0.053 (0.005)	8.6E-29	-0.038 (0.005)	2.3E-14		
rs878521	7	44,255,643	*GCK*	G	0.77	-0.062 (0.005)	1.0E-36	-0.050 (0.005)	2.8E-22	-0.058 (0.005)	3.1E-32
rs11558471	8	118,185,733	*SLC30A8*	A	0.65	0.029 (0.005)	3.2E-10	0.029 (0.005)	3.2E-10	0.029 (0.005)	3.2E-10
rs35964103	10	113,002,526	*ADRA2A*	C	0.91	0.041 (0.007)	8.5E-09	0.041 (0.007)	1.3E-08	0.046 (0.007)	2.1E-10
rs17747324	10	114,752,503	*TCF7L2*	T	0.79	-0.027 (0.005)	7.6E-08			-0.031 (0.005)	1.9E-09
rs34872471	10	114,754,071	*TCF7L2*	T	0.74	-0.027 (0.005)	2.1E-08	-0.026 (0.005)	3.1E-08		
rs7113297	11	92,671,744	*MTNR1B*	C	0.82	-0.064 (0.007)	2.6E-19	-0.041 (0.007)	2.1E-08	-0.041 (0.007)	2.2E-08
rs11020121	11	92,683,830	*MTNR1B*	T	0.77	-0.073 (0.006)	1.5E-36			-0.036 (0.007)	3.3E-08
rs10830963	11	92708710	*MTNR1B*	C	0.73	-0.084 (0.005)	1.0E-61	-0.078 (0.005)	1.3E-50	-0.063 (0.006)	1.1E-26
rs17331697	12	97,868,906	*RMST*	T	0.90	0.046 (0.007)	1.3E-11	0.05 (0.007)	1.3E-11	0.046 (0.007)	1.3E-11
rs1881415	15	62,388,530	*VPS13C-C2CD4AB-FAM148B*	T	0.52	0.023 (0.004)	1.7E-08	0.02 (0.004)	1.7E-08	0.023 (0.004)	1.7E-08

d. FI_{adjBMI}

Index SNP	Chr	Position	Locus	Effect allele	Meta-analysis			Joint analysis with LD estimated from 58BC-WTCCC		Joint analysis with LD estimated from NFBC1966	
					EAF	β (SE)	*P*	B (SE)	*P*	B (SE)	*P*
rs1260326	2	27,730,940	*GCKR*	T	0.36	-0.030 (0.005)	5.8E-11	-0.030 (0.005)	5.8E-11	-0.030 (0.005)	5.8E-11

Summary of SNPs achieving $p<5x10^{-8}$ in the joint analysis using BC58 and NFBC66 cohorts as reference samples, respectively. SNPs on different chromosomes or more than 10Mb distant are assumed to be in linkage equilibrium. Chr, chromosome; Position in NCBI build 37; EAF, effect allele frequency in meta-analysis result; β, marginal effect; B, joint effect.
