# Supplementary material for: Discovery and Fine-Mapping of Glycaemic and Obesity-Related Trait Loci Using High-Density Imputation
Source: PLoS Genet. 2015 Jul 1;11(7):e1005230. doi: 10.1371/journal.pgen.1005230 (PMC4488845; doi:10.1371/journal.pgen.1005230)
Supplement: S9 Table — (PDF) [file pgen.1005230.s019.pdf]

S9 Table. Summary of 99% credible sets at 42 distinct association signals.

Trait	Locus	Index variant	Chr	Number of variants	Distance	Interval start (b37)	Interval stop (b37)	Number (%) of variants not in HapMap	Posterior probablity of variants not in HapMap
BMI	*NEGR1*	rs11209943	1	159	567,218	72,387,666	72,954,883	87 (55%)	0.61
BMI	*SEC16B*	rs539515	1	18	33,235	177,861,357	177,894,591	9 (50%)	0.45
BMI	*TMEM18*	rs66553418	2	175	39,707	614,168	653,874	100 (57%)	0.52
BMI	*RBJ-ADCY3-POMC*	rs6749422	2	158	216,259	25,075,281	25,291,539	77 (49%)	0.54
BMI	*ETV5*	rs9816226	3	27	76,173	185,769,425	185,845,597	24 (89%)	0.83
BMI	*GNPDA2*	rs12507026	4	5	10,449	45,175,691	45,186,139	2 (40%)	0.49
BMI	*GALNT10*	rs11958496	5	43	67,279	153,497,400	153,564,678	12 (28%)	0.49
BMI	*TFAP2B*	rs3798519	6	37	132,825	50,788,778	50,921,602	27 (73%)	0.82
BMI	*DTX2P1-UPK3BP1-PMS2P11*	rs7804790	7	122	226,786	76,500,308	76,727,093	114 (93%)	0.91
BMI	*GRID1*	rs7903554	10	165	288,937	87,339,257	87,628,193	52 (32%)	0.92
BMI	*BDNF*	rs4517468	11	59	101,835	27,634,373	27,736,207	32 (54%)	0.63
BMI	*FAIM2*	rs7132908	12	17	64,526	50,215,905	50,280,430	12 (71%)	0.55
BMI	*ATP2B1*	rs1966714	12	79	120,498	90,594,389	90,714,886	32 (41%)	0.45
BMI	*AKAP6*	rs12885467	14	38	27,264	33,282,232	33,309,495	19 (50%)	0.43
BMI	*NRXN3*	rs7141420	14	17	54,707	79,890,456	79,945,162	5 (29%)	0.13
BMI	*MAP2K5*	rs4776972	15	94	319,694	67,808,076	68,127,769	49 (52%)	0.47
BMI	*GPRC5B*	rs7190603	16	44	144,936	19,832,798	19,977,733	36 (82%)	0.89
BMI	*SH2B1*	rs2008514	16	127	511,888	28,383,243	28,895,130	99 (78%)	0.76
BMI	*FTO*	rs55872725	16	40	24,030	53,798,622	53,822,651	28 (70%)	0.76
BMI	*MC4R*	rs663129	18	21	114,083	57,740,612	57,854,694	12 (57%)	0.44
WHR_{adjBMI}	*LY86*	rs1294437	6	24	21,552	6,733,540	6,755,091	13 (54%)	0.82
WHR_{adjBMI}	*VEGFA*	rs6905288	6	3	2,432	43,757,896	43,760,327	1 (33%)	0.12
WHR_{adjBMI}	*RSPO3*	rs72959041	6	4	140,680	127,389,101	127,529,780	4 (100%)	0.99
WHR_{adjBMI}	*RSPO3*	rs4509142	6	403	517,946	127,014,862	127,532,807	171 (42%)	0.34
FG	*PROX1*	rs340876	1	5	7,162	214,156,514	214,163,675	2 (40%)	0.83
FG	*GCKR*	rs1260326	2	3	21,524	27,730,940	27,752,463	1 (33%)	0.03
FG	*G6PC2*	rs560887	2	2	9,734	169,753,415	169,763,148	0 (0%)	0
FG	*G6PC2*	rs138726309	2	2	14,572	169,748,691	169,763,262	2 (100%)	0.99
FG	*SLC2A2*	rs7356034	3	102	117,012	170,627,909	170,744,920	67 (66%)	0.53
FG	*PCSK1*	rs144489757	5	35	106,333	95,630,225	95,736,557	21 (60%)	0.65
FG	*CDKAL1*	rs7747724	6	35	177,554	20,721,721	20,899,274	13 (37%)	0.32
FG	*DGKB-TMEM195*	rs13220985	7	52	49,936	15,016,110	15,066,045	27 (52%)	0.57
FG	*GCK*	rs878521	7	2	23,866	44,231,778	44,255,643	1 (50%)	0.18
FG	*GCK*	rs10259649	7	14	70,710	44,183,433	44,254,142	8 (57%)	0.40
FG	*SLC30A8*	rs11558471	8	7	33,133	118,184,783	118,217,915	4 (57%)	0.42
FG	*ADRA2A*	rs35964103	10	66	80,013	112,970,951	113,050,963	45 (68%)	0.73
FG	*TCF7L2*	rs34872471	10	27	67,276	114,749,734	114,817,009	22 (82%)	0.79
FG	*MTNR1B*	rs10830963	11	1	1	92,708,710	92,708,710	0 (0%)	0
FG	*RMST*	rs17331697	12	14	22,286	97,846,621	97,868,906	11 (79%)	0.14
FG	*VPS13C-C2CD4AB-FAM148B*	rs1881415	15	25	189,268	62,210,274	62,399,541	13 (52%)	0.49
FG (female)	*EMID2*	rs6947345	7	12	97,460	100,995,671	101,093,130	12 (100%)	0.99
FI_{adjBMI}	*GCKR*	rs1260326	2	3	21,524	27,730,940	27,752,463	1 (33%)	0.06
