# Supplementary material for: Discovery and Fine-Mapping of Glycaemic and Obesity-Related Trait Loci Using High-Density Imputation
Source: PLoS Genet. 2015 Jul 1;11(7):e1005230. doi: 10.1371/journal.pgen.1005230 (PMC4488845; doi:10.1371/journal.pgen.1005230)
Supplement: S10 Table — (PDF) [file pgen.1005230.s020.pdf]

S10 Table. Variants of 99% credible sets containing less than 20 variants driving distinct association signals for BMI, WHR_{adjBMI}, FG and FI_{adjBMI}.

Trait	Locus	Index variant	99% credible set variants							
			Variant	Chr	Position (b37)	Alleles Effect/Other	EAF	Effect (SE)	p-value	Posterior probability of driving association signal
BMI	*SEC16B*	rs539515	rs539515	1	177,889,025	C/A	0.20	0.042 (0.006)	7.0E-11	11.2%
			rs543874	1	177,889,480	G/A	0.20	0.041 (0.006)	1.1E-10	7.0%
			rs670323	1	177,868,990	A/G	0.20	0.041 (0.006)	1.2E-10	6.9%
			rs490689	1	177,873,841	A/G	0.20	0.041 (0.006)	1.2E-10	6.6%
			rs527248	1	177,875,514	G/A	0.20	0.041 (0.006)	1.2E-10	6.6%
			rs571567	1	177,872,905	A/G	0.20	0.041 (0.006)	1.2E-10	6.5%
			rs574367	1	177,873,210	T/G	0.20	0.041 (0.006)	1.3E-10	6.3%
			rs693232	1	177,881,651	G/C	0.20	0.041 (0.006)	1.4E-10	5.6%
			rs661691	1	177,861,357	C/G	0.21	0.041 (0.006)	1.6E-10	5.0%
			rs532504	1	177,878,933	A/G	0.20	0.041 (0.006)	1.6E-10	5.0%
			rs506589	1	177,894,287	C/T	0.20	0.041 (0.006)	1.7E-10	4.8%
			rs1266155	1	177,881,424	A/G	0.20	0.041 (0.006)	1.7E-10	4.8%
			rs589500	1	177,876,946	T/C	0.20	0.041 (0.006)	1.7E-10	4.8%
			rs633754	1	177,879,935	C/T	0.20	0.041 (0.006)	2.0E-10	4.1%
			rs509325	1	177,894,591	G/T	0.20	0.041 (0.006)	2.0E-10	4.1%
			rs630372	1	177,885,762	A/G	0.23	0.039 (0.006)	2.2E-10	3.6%
			rs478788	1	177,887,018	C/T	0.23	0.039 (0.006)	2.5E-10	3.1%
			rs585944	1	177,882,786	T/C	0.23	0.039 (0.006)	3.1E-10	2.5%
BMI	*GNPDA2*	rs12507026	rs12507026	4	45,181,334	T/A	0.42	0.041 (0.005)	6.2E-14	27.5%
			rs10938397	4	45,182,527	G/A	0.42	0.041 (0.005)	6.8E-14	25.1%
			rs12641981	4	45,179,883	T/C	0.42	0.041 (0.005)	7.8E-14	22.0%
			rs10938398	4	45,186,139	A/G	0.42	0.040 (0.005)	7.9E-14	21.5%
			rs13130484	4	45,175,691	T/C	0.42	0.041 (0.006)	6.7E-13	2.8%
BMI	*FAIM2*	rs7132908	rs7132908	12	50,263,148	A/G	0.38	0.037 (0.005)	9.6E-13	23.0%
			rs12146733	12	50,270,364	C/G	0.36	0.038 (0.005)	1.7E-12	13.7%
			rs1893492	12	50,270,855	G/T	0.37	0.037 (0.005)	1.9E-12	12.0%
			rs7953539	12	50,227,988	A/G	0.48	0.036 (0.005)	3.5E-12	6.5%
			rs4898534	12	50,231,025	G/A	0.48	0.036 (0.005)	3.6E-12	6.2%
			rs73116325	12	50,275,385	T/C	0.36	0.037 (0.005)	3.8E-12	6.2%
			rs7973894	12	50,233,256	A/G	0.48	0.036 (0.005)	3.8E-12	5.9%
			rs10875976	12	50,226,467	A/G	0.48	0.036 (0.005)	4.3E-12	5.2%
			rs4898535	12	50,231,057	G/T	0.48	0.036 (0.005)	5.0E-12	4.5%
			rs112502508	12	50,246,252	G/A	0.38	0.036 (0.005)	6.4E-12	3.6%
			rs11169170	12	50,223,013	T/C	0.48	0.036 (0.005)	8.3E-12	2.8%
			rs12367809	12	50,256,063	T/C	0.38	0.036 (0.005)	9.3E-12	2.5%
			rs11169199	12	50,280,430	C/T	0.38	0.036 (0.005)	9.3E-12	2.5%
			rs55677077	12	50,219,508	C/T	0.50	0.035 (0.005)	1.6E-11	1.4%
			rs11169166	12	50,215,905	T/C	0.55	0.034 (0.005)	2.3E-11	1.0%
			rs3205718	12	50,261,809	T/C	0.38	0.036 (0.005)	2.7E-11	0.9%
			rs141070800	12	50,272,835	G/A	0.43	0.037 (0.006)	4.5E-11	0.6%
BMI	*NRXN3*	rs7141420	rs7141420	14	79,899,454	T/C	0.52	0.035 (0.005)	8.3E-12	62.2%
			rs10146997	14	79,945,162	G/A	0.23	0.039 (0.006)	1.9E-10	3.5%

			rs10136360	14	79,894,046	A/G	0.22	0.039 (0.006)	1.9E-10	3.4%
			rs10150482	14	79,891,882	A/G	0.22	0.039 (0.006)	2.0E-10	3.3%
			rs7144011	14	79,940,383	T/G	0.22	0.039 (0.006)	2.2E-10	3.0%
			rs8020365	14	79,937,216	A/T	0.22	0.039 (0.006)	2.3E-10	2.9%
			rs10145154	14	79,939,525	T/C	0.22	0.039 (0.006)	2.6E-10	2.6%
			rs10150332	14	79,936,964	C/T	0.22	0.039 (0.006)	2.6E-10	2.5%
			rs17836088	14	79,932,041	C/G	0.22	0.039 (0.006)	2.8E-10	2.4%
			rs2370982	14	79,890,677	T/C	0.22	0.039 (0.006)	2.8E-10	2.3%
			rs72690737	14	79,930,644	C/T	0.22	0.039 (0.006)	2.9E-10	2.3%
			rs28479795	14	79,943,606	T/C	0.22	0.039 (0.006)	2.9E-10	2.3%
			rs8008910	14	79,944,099	A/G	0.22	0.039 (0.006)	3.1E-10	2.2%
			rs17109221	14	79,910,119	T/C	0.22	0.039 (0.006)	5.8E-10	1.2%
			rs10146690	14	79,890,456	A/G	0.22	0.039 (0.006)	6.6E-10	1.0%
			rs7156625	14	79,942,647	A/G	0.22	0.039 (0.006)	7.5E-10	0.9%
			rs17109256	14	79,939,993	A/G	0.22	0.039 (0.006)	7.6E-10	0.9%
WHR_{adjBMI}	*VEGFA*	rs6905288	rs6905288	6	43,758,873	A/G	0.56	0.043 (0.007)	4.9E-11	79.3%
			rs11967262	6	43,760,327	G/C	0.49	0.041 (0.007)	3.3E-10	12.2%
			rs998584	6	43,757,896	A/C	0.49	0.040 (0.007)	7.6E-10	5.4%
WHR_{adjBMI}	*RSPO3*	rs72959041	rs72959041	6	127,454,893	A/G	0.08	0.100 (0.016)	2.5E-10	78.6%
			rs145881926	6	127,389,101	A/G	0.06	0.110 (0.018)	2.8E-09	8.3%
			rs72961013	6	127,529,780	A/G	0.09	0.087 (0.015)	3.2E-09	6.3%
			rs72961007	6	127,524,398	A/C	0.09	0.086 (0.015)	3.6E-09	5.7%
FG	*PROX1*	rs340876	rs340876	1	214,158,132	T/C	0.55	0.028 (0.004)	1.3E-11	76.9%
			rs340874	1	214,159,256	C/T	0.53	0.029 (0.004)	8.8E-11	13.2%
			rs340877	1	214,157,246	A/G	0.56	0.026 (0.004)	1.7E-10	6.4%
			rs340835	1	214,163,675	A/G	0.46	0.025 (0.004)	6.7E-10	1.7%
			rs340879	1	214,156,514	C/T	0.62	0.025 (0.004)	1.6E-09	0.7%
FG	*GCKR*	rs1260326	rs1260326	2	27,730,940	C/T	0.64	0.033 (0.004)	2.2E-15	93.5%
			rs780094	2	27,741,237	C/T	0.65	0.031 (0.004)	8.5E-14	2.6%
			rs11127048	2	27,752,463	A/G	0.64	0.031 (0.004)	8.8E-14	2.6%
FG	*G6PC2*	rs560887	rs560887	2	169,763,148	C/T	0.69	0.088 (0.005)	2.2E-66	79.5%
			rs13431652	2	169,753,415	T/C	0.69	0.079 (0.005)	8.8E-66	20.5%
FG	*G6PC2*	rs138726309	rs150171632	2	169,748,691	C/T	0.99	0.210 (0.021)	7.6E-24	88.1%
			rs138726309	2	169,763,262	C/T	0.99	0.214 (0.022)	5.7E-23	11.2%
FG	*GCK*	rs878521	rs2971669	7	44,231,778	T/C	0.18	0.052 (0.006)	3.0E-19	81.2%
			rs878521	7	44,255,643	A/G	0.23	0.046 (0.005)	1.3E-18	18.1%
FG	*GCK*	rs10259649	rs2268577	7	44,189,010	T/C	0.18	0.035 (0.006)	2.2E-10	17.1%
			rs2908293	7	44,209,322	A/G	0.22	0.032 (0.005)	2.1E-10	16.2%
			rs2908292	7	44,210,710	T/C	0.22	0.032 (0.005)	2.4E-10	14.2%
			rs2300584	7	44,219,338	G/A	0.22	0.032 (0.005)	2.8E-10	12.6%
			rs2971671	7	44,211,337	C/T	0.22	0.032 (0.005)	3.3E-10	10.4%
			rs76323047	7	44,185,955	G/A	0.14	0.037 (0.006)	5.2E-10	7.8%
			rs10259649	7	44,219,705	C/T	0.22	0.032 (0.005)	4.6E-10	7.7%
			rs2908294	7	44,204,426	T/C	0.22	0.031 (0.005)	6.2E-10	5.7%
			rs6971410	7	44,191,190	A/T	0.20	0.033 (0.005)	1.5E-09	2.6%
			rs2268575	7	44,189,274	C/T	0.18	0.033 (0.006)	1.9E-09	2.1%
			rs2908277	7	44,183,433	A/G	0.14	0.036 (0.006)	2.0E-09	2.1%

			rs3857752	7	44,240,138	C/G	0.30	0.028 (0.005)	1.7E-08	0.2%
			rs77888691	7	44,231,570	G/T	0.07	0.046 (0.008)	3.2E-08	0.2%
			rs74489265	7	44,254,142	G/T	0.07	0.046 (0.008)	4.1E-08	0.1%
FG	*SLC30A8*	rs11558471	rs11558471	8	118,185,733	A/G	0.65	0.029 (0.005)	3.2E-10	38.0%
			rs35859536	8	118,191,475	C/T	0.66	0.026 (0.004)	8.5E-10	13.6%
			rs4300038	8	118,217,915	G/A	0.67	0.027 (0.004)	8.8E-10	13.3%
			rs3802177	8	118,185,025	G/A	0.66	0.028 (0.005)	1.3E-09	9.9%
			rs13266634	8	118,184,783	C/T	0.66	0.026 (0.004)	1.3E-09	8.8%
			rs9650069	8	118,204,020	C/T	0.66	0.026 (0.004)	1.4E-09	8.7%
			rs185166635	8	118,209,284	C/A	0.65	0.026 (0.004)	1.9E-09	6.2%
FG	*MTNR1B*	rs10830963	rs10830963	11	92,708,710	G/C	0.27	0.084 (0.005)	1.0E-61	100.0%
FG	*RMST*	rs17331697	rs17331697	12	97,868,906	T/C	0.90	0.046 (0.007)	1.3E-11	49.1%
			rs17390909	12	97,868,654	C/G	0.90	0.046 (0.007)	1.8E-11	35.8%
			rs79333258	12	97,851,096	C/G	0.94	0.050 (0.008)	4.9E-10	1.6%
			rs74628648	12	97,851,002	C/T	0.94	0.050 (0.008)	4.9E-10	1.6%
			rs78551082	12	97,850,964	C/T	0.94	0.050 (0.008)	4.9E-10	1.6%
			rs76369685	12	97,850,590	G/A	0.94	0.050 (0.008)	4.9E-10	1.6%
			rs79896666	12	97,850,366	A/C	0.94	0.050 (0.008)	5.1E-10	1.6%
			rs78556151	12	97,854,967	T/A	0.94	0.050 (0.008)	5.5E-10	1.5%
			rs721156	12	97,864,143	C/T	0.94	0.049 (0.008)	6.4E-10	1.3%
			rs79621919	12	97,860,592	G/A	0.94	0.049 (0.008)	6.4E-10	1.3%
			rs77530740	12	97,867,705	A/T	0.94	0.049 (0.008)	9.3E-10	0.9%
			rs113036477	12	97,848,227	C/T	0.94	0.049 (0.008)	1.9E-09	0.4%
			rs76729185	12	97,846,621	C/A	0.94	0.048 (0.008)	2.7E-09	0.3%
			rs1592499	12	97,852,758	A/C	0.94	0.052 (0.009)	3.2E-09	0.3%
FG (female)	*EMID2*	rs6947345	rs6947345	7	101,071,933	C/T	0.98	0.162 (0.029)	3.8E-08	96.9%
			rs114665599	7	101,053,697	G/A	0.99	0.152 (0.033)	3.5E-06	1.5%
			rs10225895	7	101,080,888	C/T	0.98	0.139 (0.033)	2.1E-05	0.4%
			rs10215646	7	101,093,130	C/T	0.98	0.127 (0.034)	2.0E-04	0.05%
			rs28623299	7	101,032,556	G/A	0.98	0.095 (0.028)	7.1E-04	0.02%
			rs28681238	7	101,032,555	T/C	0.98	0.095 (0.028)	7.1E-04	0.02%
			rs111557658	7	100,999,090	C/T	0.98	0.118 (0.035)	7.1E-04	0.02%
			rs76243984	7	101,011,933	C/T	0.98	0.118 (0.035)	7.1E-04	0.02%
			rs28538298	7	100,995,671	C/G	0.99	0.120 (0.035)	7.1E-04	0.02%
			rs111363172	7	101,011,632	G/A	0.98	0.092 (0.028)	1.1E-03	0.01%
			rs28588772	7	100,998,658	C/T	0.98	0.092 (0.028)	1.1E-03	0.01%
			rs145509296	7	101,003,801	T/G	0.98	0.092 (0.028)	1.1E-03	0.01%
Fi_{adjBMI}	*GCKR*	rs1260326	rs1260326	2	27,730,940	C/T	0.64	0.030 (0.005)	5.8E-11	89.6%
			rs11127048	2	27,752,463	A/G	0.64	0.028 (0.005)	8.7E-10	6.5%
			rs780093	2	27,742,603	A/T	0.64	0.027 (0.005)	2.9E-09	2.0%
