# Supplementary material for: Discovery and Fine-Mapping of Glycaemic and Obesity-Related Trait Loci Using High-Density Imputation
Source: PLoS Genet. 2015 Jul 1;11(7):e1005230. doi: 10.1371/journal.pgen.1005230 (PMC4488845; doi:10.1371/journal.pgen.1005230)
Supplement: S11 Table — (PDF) [file pgen.1005230.s021.pdf]

S11 Table. Allele frequency distribution of 99% credible sets with less than 20 variants.

Minor allele frequency (MAF)[a]	Number (%) of HapMap variants	Number (%) of variants not in HapMap	Number (%) of all 1000G variants
MAF<0.005	0	0	0
0.005≤MAF<0.01	0	0	0
0.01≤MAF<0.02	0	13 (18%)	13 (10%)
0.02≤MAF<0.03	0	1 (1.4%)	1 (0.8%)
0.03≤MAF<0.04	0	1 (1.4%)	1 (0.8%)
0.04≤MAF<0.05	0	0	0
0.05≤MAF<0.1	3 (6%)	16 (22%)	19 (15%)
0.1≤MAF<0.2	12 (23%)	8 (11%)	20 (16%)
0.2≤MAF<0.3	16 (30%)	13 (18%)	29 (23%)
0.3≤MAF<0.4	10 (19%)	12 (16%)	22 (17%)
0.4≤MAF<0.5	12 (23%)	10 (14%)	22 (17%)
Total	53	74	127

[a] MAF is based on MAF reported in the meta-analysis result.
