# Supplementary material for: Discovery and Fine-Mapping of Glycaemic and Obesity-Related Trait Loci Using High-Density Imputation
Source: PLoS Genet. 2015 Jul 1;11(7):e1005230. doi: 10.1371/journal.pgen.1005230 (PMC4488845; doi:10.1371/journal.pgen.1005230)
Supplement: S12 Table — (PDF) [file pgen.1005230.s022.pdf]

S12 Table. Power to detect association, at genome-wide significance (p<5x10^{-8}), with a variant of MAF 1% in the current study.

Trait	Max *N*	Variant explaining 0.05% of the trait variance	Variant explaining 0.1% of the trait variance	Variant explaining 0.2% of the trait variance
BMI	87,084	0.880	0.999	0.999
WHR_{adjBMI}	54,572	0.421	0.975	0.999
FG	46,694	0.277	0.921	0.999
FI_{adjBMI}	24,245	0.026	0.309	0.939
